# Overcoming the Reversibility in the Diels–Alder Reaction of Bio-Based Electron-Poor Furans with Maleimides Through Liquid-to-Solid Phase Transition

**DOI:** 10.3390/ijms26146550

**Published:** 2025-07-08

**Authors:** Konstantin I. Galkin, Daria V. Zakharova, Rinat R. Aysin, Anastasia A. Danshina, Alexandra M. Pak, Irina V. Sandulenko, Roman A. Novikov, Ksenia S. Egorova

**Affiliations:** 1 N.D. Zelinsky Institute of Organic Chemistry, Russian Academy of Sciences, Leninsky Prospekt, 47, 119991 Moscow, Russia; roman@ioc.ac.ru; 2 Center for Project Activities, Moscow Polytechnic University (Moscow Polytech), Bolshaya Semyonovskaya Str., 38, 107023 Moscow, Russia; 3 NTI Center “Digital Materials Science: New Materials and Substances”, Bauman Moscow State Technical University, 2nd Baumanskaya Str., 5, 105005 Moscow, Russia; dzakharova@bmstu.ru; 4 A.N. Nesmeyanov Institute of Organoelement Compounds, Russian Academy of Sciences, Vavilova Str., 28, bld. 1., 119991 Moscow, Russia; aysin@ineos.ac.ru (R.R.A.); danshina.aa@phystech.edu (A.A.D.); alexunnipark@gmail.com (A.M.P.); ira17.rock@mail.ru (I.V.S.)

**Keywords:** plant biomass, furfural, maleimides, Diels–Alder reaction, differential scanning calorimetry

## Abstract

In the chemistry of bio-based furans, the Diels–Alder reaction plays an important role as a renewable route for the synthesis of fuels, fine chemicals, and monomers. Nonetheless, the unfavorable kinetic and thermodynamic parameters inherent to the Diels–Alder reaction involving furans as dienes often lead to the reversibility of cycloaddition, resulting in decreased equilibrium conversion and diastereoselectivity. In this study, we present a new strategy for overcoming the problem of reversibility in chemical reactions. We demonstrate that conducting the reaction under solvent-free conditions can facilitate the transition from a molten state formed by the initial reactants to a solid phase containing the reaction product along with an excess of the initial substrate. According to our results, such a liquid-to-solid transition of the reaction mixture can lead to exceptionally high conversion and diastereoselectivity in the furan–maleimide Diels–Alder reaction, particularly for challenging electron-poor furanic substrates. Our approach enables the reversible furan–maleimide Diels–Alder reaction to be performed in a cleaner and more environmentally friendly manner, free from the complexities associated with the use of solvents and the need for purification from side products.

## 1. Introduction

The reversibility problem arising from kinetic and/or thermodynamic limitations significantly reduces the synthetic potential of many chemical reactions. One such crucial reaction is the [4 + 2]-cycloaddition between diene and dienophile, known as the Diels–Alder (DA) reaction, which has been extensively employed for the synthesis of functional or dynamic molecular and macromolecular systems [1,2,3,4,5]. In recent decades, bio-based furanic platform chemicals, such as furfural [6,7,8], 5-(hydroxymethyl)furfural [9,10,11,12,13], 2,5-furandicarboxylic acid [14,15,16], and their derivatives, which can be obtained by catalytic or biocatalytic conversion of plant biomass [9,17,18,19,20,21], have been attracting considerable attention as renewable building blocks. The use of these furans in the DA reaction with alkene dienophiles provides renewable access to bio-based fine chemicals, monomers, and polymers [22,23,24,25]. Furan–alkene DA adducts (7-oxanorbornenes) can be further transformed into functional aromatics through catalytic dehydration or tandem catalytic hydrogenation/oxidative aromatization [26,27,28,29,30,31,32,33,34,35]. Additionally, the reduction of the double bond in tricyclic 7-oxanorbornenes yields biologically active norcantharidin derivatives [36,37,38,39]. Some furan–maleimide Diels–Alder (fmDA) adducts demonstrated reactivity in ring-opening metathesis polymerization (ROMP), resulting in unsaturated stereoregular polymers [40,41,42]. Functional fmDA adducts have been widely used as monomers for dynamic polymers, known as dynamers, which have garnered significant interest due to their unique properties, such as self-healing and shape memory effects, as well as enhanced re-processing and recycling capabilities [1,43,44,45,46]. Recent research focuses on designing self-healing polymer materials with 1,2,4-triazoline-3,5-dione derivatives emerging as efficient DA “click” chemistry agents for polymer functionalization at room temperature, unlike traditional fmDA reactions that require heat [47].

The mechanism of the DA reaction involves the interaction between the highest occupied molecular orbital (HOMO) of the diene and the lowest unoccupied molecular orbital (LUMO) of the dienophile, leading to the formation of a new six-membered ring. The use of furans as dienes in the DA reaction poses inherent kinetic and thermodynamic limitations due to their aromatic nature [48,49,50,51]. The insufficient stability of furanic DA adducts can lead to decreased equilibrium conversion and/or low diastereoselectivity [48,49,50,51,52]. Furthermore, the issue of reversibility is particularly relevant for DA reactions with acceptor-substituted furans, which demonstrate appreciable reactivity only with highly electron-deficient dienophiles, such as dimethyl acetylenedicarboxylate [53,54,55], maleimides [56,57], or arynes [58,59,60,61].

Several strategies have been employed to address the reversibility issues in the fmDA reaction involving low-reactive furans with electron-withdrawing substituents. One approach involves modifying functional groups of the furanic ring to decrease its electron-accepting ability [62]. For example, the functionalization of furanic aldehydes, which do not enter the Diels–Alder reaction, into dimethylhydrazone allows for the formation of fmDA adducts that can undergo spontaneous dehydration into benzene derivatives [63,64]. Recently, Bruijnincx and colleagues reported an elegant method to overcome the thermodynamic limitations in fmDA reactions with furfurals by in situ water-mediated conversion of the 2-formyl group into a geminal diol [65]. This research group also noted a significant increase in the efficiency of the fmDA reaction with furoic acid and derivatives by employing water as a solvent. According to the authors, the beneficial impact of water is attributed to its ability to stabilize the fmDA adducts and transition states through hydrogen bonding for water-soluble furans, as well as through hydrophobic effects and hydrogen bonding at the interface for water-insoluble furans [57].

**Scheme 1:**
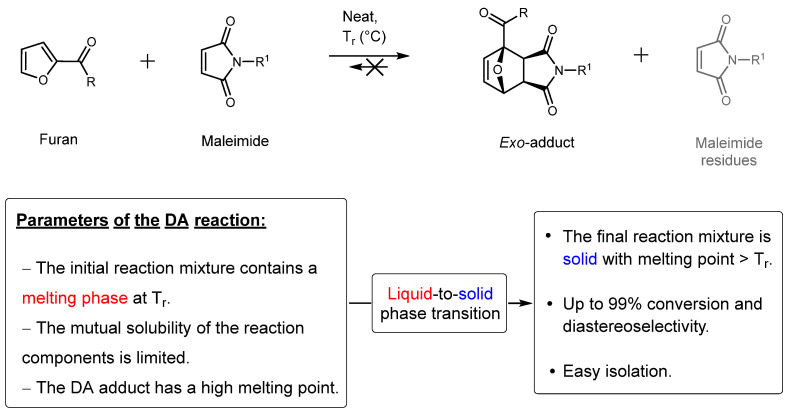
Parameters of the DA reaction required for a liquid-to-solid phase transition of the reaction mixture. In this work, we describe the application of this phenomenon to shift the equilibrium towards the *exo*-adduct in the fmDA reaction with electron-poor furans. T_r_ = reaction temperature.

In this study, we report our observation that the DA reaction between challenging electron-poor furans and maleimides can achieve remarkably high conversion and diastereoselectivity under neat conditions (Scheme 1). We consider this phenomenon to be caused by a spontaneous phase transition of the reaction mixture, which is a melt formed by the starting substrates (furan and maleimide) at the beginning of the reaction, to a solid phase at the end of the reaction. This resulting solid phase, formed by the precipitation or crystallization of an *exo*-adduct, has a melting temperature that is higher than that of the reaction, and we assume that this factor hinders the retro-DA reaction because of the absence of significant amounts of a molten phase in the reaction medium. In contrast to conventional solution-phase approaches for fmDA reactions with acceptor-substituted furans, our solvent-free methodology reduces the need for solvents while often achieving superior conversions. The combination of melt-phase conditions and spontaneous product crystallization/precipitation provides a more sustainable approach that avoids purification challenges associated with solution-phase systems, thus aligning with green chemistry principles. We propose the application of a liquid-to-solid phase transition under solvent-free conditions as a novel approach to overcoming the reversibility problem in chemical reactions.

## 2. Results and Discussion

The reversibility of the Diels–Alder reaction significantly limits the synthetic potential of bio-based furans in fine organic synthesis and material development. This challenge has prompted us to explore new strategies addressing the reversibility issue, particularly for the fmDA reaction with acceptor-substituted substrates. In this study, we focused on electron-deficient furfural-derived furans and solvent-free conditions, which were proven to be highly efficient for the DA reactions involving electron-rich furans [39,49,66,67,68].

Initially, we investigated the reactions of methyl 2-furoate with various alkene dienophiles, including acrylamide, acrylonitrile, dimethyl maleate, maleimide, and *N*-ethylmaleimide, in acetone, water, and under neat conditions. A 50% excess of dienophile was employed, and the temperature was maintained in the range of 50–80 °C, which was identified as optimal for the fmDA cycloaddition with electron-poor furans [33,56,57,69,70,71,72]. However, acrylamide, acrylonitrile, and dimethyl maleate did not react with methyl 2-furoate under the specified conditions (Table S1, entries 1–3).

Consistent with the previously published data [57], the degrees of conversion of methyl 2-furoate in the reaction with maleimide or *N*-ethylmaleimide in acetone were low (Table 1, entries 1, 2), while the use of water produced the corresponding adducts *exo*-**1a** and *exo*-**1b** in good yields (Table 1, entries 3, 4). The reactions without the solvent exhibited the highest efficiency, with the maximum yields of *exo*-**1a** and *exo*-**1b** achieved using 1.1–1.25 equivalents of the corresponding maleimide at 60 °C (Table 1, entries 6, 11, 13, and Table S2). Notably, decreasing the reaction temperature by 10 degrees or increasing it by 20 degrees significantly reduced the conversion of methyl 2-furoate (Table 1, entries 5, 10, 16). In contrast, extending the reaction time from one to three days led to an increase in the conversion degrees (Table 1, entries 8, 11, 13, 15). Five maleimides with melting points lower than 100 °C were selected for further investigation to facilitate the formation of sufficient amounts of a liquid molten phase under neat conditions at optimal reaction temperatures (Table 1). The use of *N*-(2-hydroxyethyl)maleimide (HEM) and *N*-benzylmaleimide resulted in high yields of the corresponding adducts *exo*-**1c** and *exo*-**1d** (Table 1, entries 17–19). However, with *N*-phenylmaleimide, adduct *exo*-**1e** was obtained in a noticeably lower yield (Table 1, entry 20).

The evaluation of solvent-free conditions was also conducted for other acceptor-substituted furans, including 2-furoic acid, 2-furamide, and 2-acetylfuran. These experiments yielded a series of fmDA adducts **2**(**a**–**e**)–**4**(**a**–**e**) with varying efficiencies (Table 2; see also Tables S3–S5 in the Supporting Information). Similar to methyl 2-furoate, the outcomes of the fmDA reactions with 2-furoic acid were highly dependent on the reaction conditions and the maleimide used. Under optimized conditions, high degrees of conversion were observed for adducts *exo*-**2**(**a**–**c**) and *exo*-**2e**, while the reaction of 2-furoic acid with *N*-benzylmaleimide showed lower efficiency (Table 2, entries 1–8). In contrast, 2-furamide exhibited very high reactivity towards maleimides, resulting in the formation of adducts *exo*-**3**(**a**–**e**) with high yields and selectivity across a wide range of conditions (Table 2, entries 9–15). Conversely, 2-acetylfuran demonstrated significantly lower degrees of conversion with all the maleimides tested (Table 2, entries 16–20).

To investigate the versatility of our method for *N*-substituted maleimides with high melting points, we conducted the fmDA reactions of acceptor-substituted furans with *N*-(4-hydroxyphenyl)maleimide (HPM), the melting point of which was 182–184 °C [78]—that is, significantly higher than the optimal reaction temperature. At temperatures up to 160 °C, this maleimide did not melt during the fmDA reaction and remained solid. No noticeable yield of the corresponding fmDA adduct was observed at 80–140 °C in the reaction of HPM with methyl 2-furoate, with a low yield of 8% achieved at 160 °C (Table S6, entries 1–4, 9). Unfortunately, the attempts to improve the yields of adducts with HPM by varying the furanic substrates and reaction conditions were unsuccessful (Table S6). The maximum yield of the fmDA adduct was about 19%, with a conversion of about 42%, and was obtained in the reaction between 2-furamide and 1.5 equivalents of HPM at 160 °C (Table S6, entry 8). The necessity to select maleimides with appropriate melting points seems to be a limitation of the fmDA reaction under neat conditions.

Some of the results obtained in the fmDA reactions with acceptor-substituted furans under neat conditions did not align with the existing literature data. The high influence of temperature and time on the studied fmDA reactions with acceptor-substituted furans, such as the maximum conversion at a temperature range of 50–80 °C and a long reaction time, corresponded to the previously reported data [57,69]. However, certain findings of the present work remained difficult to explain. For example, 2-furamide exhibited high reactivity with all the tested maleimides across a wide range of conditions (Table 2, entries 9–15, and Table S4). But it was unclear why 2-furoic acid, which has very similar electronic and steric properties to 2-furamide, demonstrated significantly lower degrees of conversion under the same conditions (compare, for example, Table S2, entries 16, 23–27 with Table S3, entries 8–20). Another question arose from the decreased reactivity of *N*-phenylmaleimide and 2-acetylfuran compared to the other tested substrates. Contrary to our results, *N*-phenylmaleimide had similar or greater activity compared to *NH*- and *N*-alkyl maleimides in DA reactions with furans [69,79,80].

To investigate the mechanistic reasons behind the decreased reactivity of *N*-phenylmaleimide and 2-acetylfuran, and to correlate the experimental results with the energetic profiles of the studied fmDA reactions, we conducted DFT calculations for the formation of fmDA adducts *exo*-**1**(**b**, **e**) and *exo*-**4**(**b**, **e**) (see Table 3 and the Supporting Information). The positive ΔG^o^_298K_ values for the formation of these *exo*-adducts and the transition state activation energies (ΔG^≠^) of approximately 25 kcal/mol matched the optimal temperature range observed for the direct fmDA reaction involving acceptor-substituted furans (50–80 °C). At the same time, only slight differences in the thermodynamic parameters and activation energies (± 2 kcal/mol) were noted for the studied systems. These results indicated similar kinetic and thermodynamic stabilities of fmDA adducts *exo*-**1**(**b**, **e**) and *exo*-**4**(**b**, **e**) obtained from both *N*-phenyl- and *N*-ethyl-maleimides, which was in agreement with the previously reported data [51]. Therefore, the decreased degrees of conversion for *N*-phenylmaleimide and 2-acetylfuran could not be fully explained by mechanistic considerations based on these DFT calculations.

**Figure 1:**
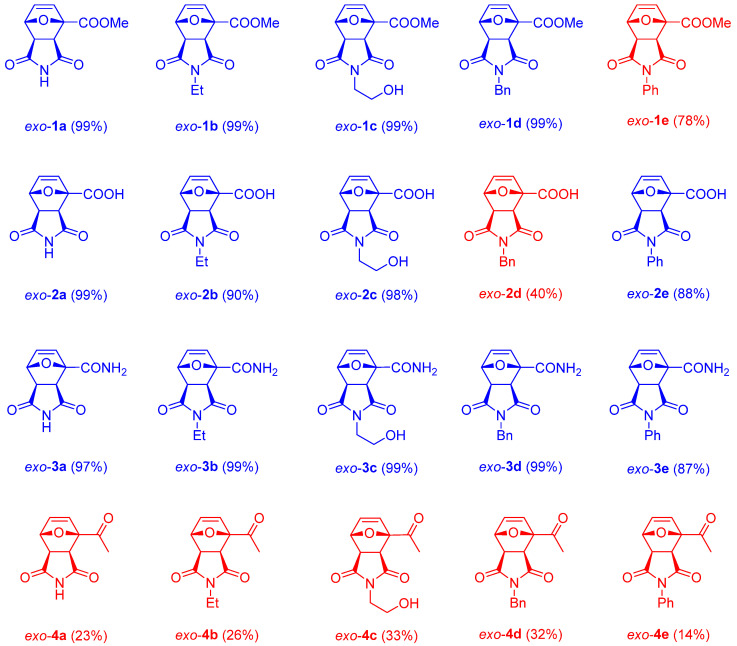
The impact of the phase composition of reaction mixtures on the selectivity of the synthesized fmDA adducts *exo*-(**1**–**4**). The best selectivity values specified in Table 1 and Table 2 are provided in parentheses. For the compounds shown in blue, the resulting reaction mixture was solid with small or nondetectable amounts of a molten phase. For the compounds shown in red, the reaction mixture was a viscous liquid at the end of the reaction.

Based on the obtained experimental and theoretical results, we aimed to investigate further why the combinations of furan and maleimide substrates with similar energetic properties performed differently in the fmDA reactions under solvent-free conditions, in some cases, upon even minor adjustments in the reaction parameters. To explain this phenomenon, we addressed distinct visual differences in the aggregate state of the resulting reaction mixtures, revealing a clear correlation between the phase composition and the degree of conversion of the initial substrates. Initially, the reaction consisted of viscous liquids or two-phase mixtures. By the end of the reaction, mixtures with high conversion precipitated at the reaction temperature, resulting in a solid with a minimal or undetectable liquid phase. This solidification over time was noted for the fmDA adducts depicted in blue in Figure 1 under optimized conditions. In contrast, mixtures with moderate or low conversion remained viscous liquids or exhibited significant amounts of a molten phase at the end of the reaction, as seen for the adducts depicted in red in Figure 1 and some other adducts depicted in blue under non-optimized conditions (see Table 1 and Table 2, and Tables S2–S5). Using a melting point apparatus, we analyzed the thermal phase transition in the high-yielding solid reaction mixtures obtained under optimized conditions, which contained predominantly *exo*-adducts and 10–50 mol. % of the corresponding maleimide. Upon heating to the reaction temperature, no visible changes were observed, or only gradual thermal softening of the reaction mixtures occurred without apparent formation of a significant amount of a molten phase.

In the case of adducts *exo*-**1**(**a**–**d**) and *exo*-**2b** derived at 60 °C from methyl 2-furoate and 2-furoic acid, respectively, the solidification of the reaction mixtures typically took considerable time (three days), producing substantial amounts of crystalline material. An X-ray analysis of samples from the reaction of methyl 2-furoate with 1.5 eq. of *N*-ethylmaleimide at 60 °C (Table 1, entry 15) confirmed the presence of crystals of *exo*-**1b** (Figure S20). The resulting mixtures obtained after crystallization of *exo*-**2b** from the reaction medium contained small visible amounts of a melt at the reaction temperature, while the reactions producing adducts *exo*-**1**(**a**–**d**) were solid without observable amounts of a liquid/molten phase. It should be noted that the reproducibility of crystallization of adduct *exo*-**1c** during the reaction was imperfect; in some cases, the reactions remained viscous liquids and exhibited significantly lower conversion degrees (Table 1, entries 17, 18). The reaction mixtures containing adducts *exo*-**2**(**a**, **c**, **e**) and *exo*-**3**(**a**–**e**) obtained at temperatures of 80–100 °C solidified more rapidly—typically within six hours at 80 °C and one hour at 100 °C—as homogeneous precipitates without a molten phase. After full precipitation of the reaction mixtures, these adducts were obtained in high yields and with high reproducibility.

To further investigate the visually observed thermal changes in the phase composition, we analyzed samples of reaction mixtures obtained under various conditions using differential scanning calorimetry (DSC; for more details, see the Supporting Information). This thermoanalytical method measures the difference in heat absorbance between a sample and a reference upon increasing or decreasing the temperature. Consequently, any endothermic or exothermic processes occurring in an inorganic or organic substance or polymer can be detected by DSC. This technique is capable of measuring temperatures and enthalpies of chemical reactions and various phase changes, such as softening, melting, and crystallization, for both pure components and mixtures [81,82,83].

The aim of these DSC experiments was to assess the molten phase content in the reaction mixtures and to correlate it with the conversion values under different conditions. Solid samples of the reaction mixtures characterized by high conversion and the absence of visible liquid or molten phases at the reaction temperature typically exhibited one prominent endothermic peak in their DSC curves near the melting temperature of the pure adduct, along with or without a smaller endothermic peak near the melting temperature of the maleimide used (see, for example, Figures S1, S4, S9, S13, S15, S16 and S18). The small peak presumably corresponded to the softening or melting of the residual maleimide-enriched phase, while the main peak indicated the melting of the adduct-enriched phase following the retro-DA reaction [67,84]. The absence or presence of only a small endothermic peak in the DSC curves of solid reaction mixtures near or below the reaction temperature confirmed the visually observed absence of a significant amount of a molten phase at this temperature.

**Figure 2:**
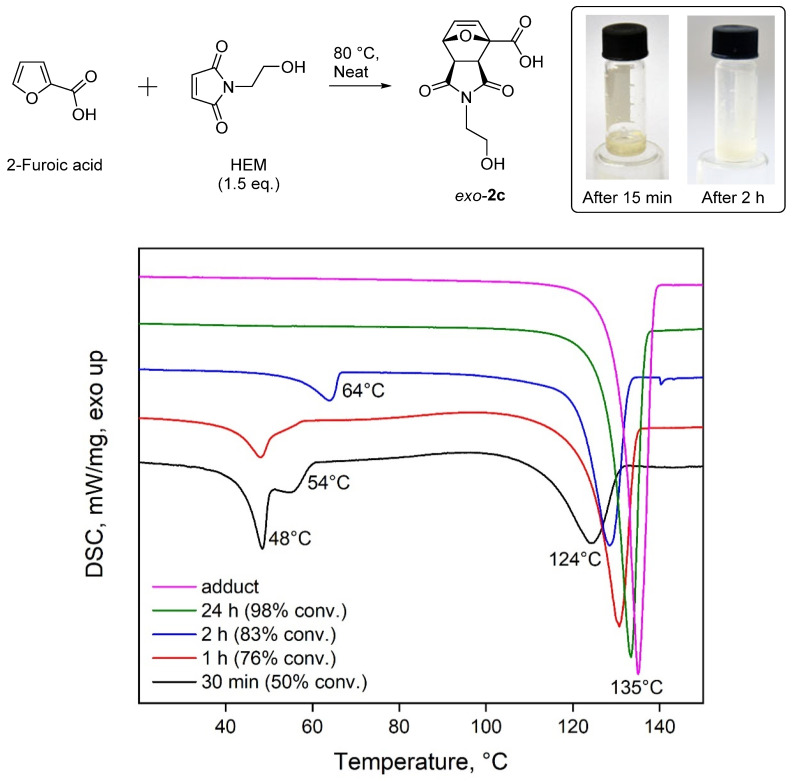
DSC monitoring and visual changes in the aggregate state of the reaction of 2-furoic acid with 1.5 eq. of HEM at 80 °C under neat conditions.

Only one broadened peak near the melting temperature of the pure adduct was observed in the DSC diagrams of solid samples of the reaction mixtures containing adduct *exo*-**2a** or *exo*-**2c** obtained from 2-furoic acid with 1.1–1.5 eq. of maleimide (Figure S8) or HEM, respectively. We conducted the monitoring of these reaction mixtures by NMR and DSC, which clearly demonstrated the liquid-to-solid phase transition process (Figure 2, Figure S7, Table S2). Initially, the reaction mixture of 2-furoic acid with HEM at 80 °C was in a molten state, formed by the starting substrates. A DSC analysis of this reaction mixture after 30 min (black DSC curve in Figure 2) revealed a complex peak with a maximum at 48 °C, likely corresponding to the melting of the HEM-enriched phase containing the starting components (HEM and 2-furoic acid), as well as possibly some amount of the dissolved adduct **2c**. The second prominent peak at 124 °C was attributed to the melting of the *exo*-adduct-enriched phase and the retro-DA process. After one hour, a two-phase system emerged, consisting of both melt and solid, corresponding to a 76% degree of conversion. The DSC diagram for this mixture (red curve in Figure 2) showed two broadened peaks corresponding to the maleimide- and adduct-enriched phases at maximum temperatures near 50 °C and 130 °C, respectively. The ratio of peak intensities shifted towards the peak of the high-melting adduct-enriched phase, which aligned with the visually observed decrease in the content of the molten phase in this reaction mixture. After two hours, a further increase in the conversion to 83% was observed, along with the elevated melting point of the maleimide-enriched phase from 48 °C to 64 °C, as illustrated by the blue DSC diagram in Figure 2. This change in the melting point may be attributed to the increase in the content of the high-melting *exo*-**2c** in this phase. After 24 h, the reaction mixture solidified completely, with no observable melting phase remaining; it was characterized by 98% conversion and 100% *exo*-diastereoselectivity. This final reaction mixture displayed only a single broadened endothermic peak at 132 °C in the DSC curve, corresponding to a high-melting phase containing *exo*-**2c** and HEM residues (green line in Figure 2).

Similar changes in the DSC thermograms were observed during the reaction of 2-furoic acid with 1.5 equivalents of maleimide at 80 °C. These DSC diagrams contained only a single broadened endothermic peak at the end of the reaction (Figure S7). The presence of only one peak in these DSC curves may suggest that the solidified reaction mixtures containing adducts *exo*-**2a**/*exo*-**2c** and 10–50 mol. % of the residual maleimide/HEM formed as a single phase. To determine the phase composition of these reaction mixtures, the reactions of 2-furoic acid with 1.25–1.5 eq. of maleimide were studied by powder X-ray diffraction (PXRD) and solid-state nuclear magnetic resonance (ssNMR). An analysis of the samples showed no new peaks in the PXRD (Figure S21) and ssNMR spectra compared to the pure adduct *exo*-**2a** and the pure unsubstituted maleimide (Figures S63–S65). These results (the presence of the single peak in the DSC curves and the absence of signals of new phases in the ssNMR and PXRD spectra) suggest that the solidified reaction mixtures obtained from 2-furoic acid and 1.1–1.5 eq. maleimide or HEM may be considered single-phase homogeneous mixtures composed of adduct *exo*-**2a**/*exo*-**2c** and the excess of the corresponding maleimide. A DSC analysis of the reaction mixtures that contained a molten phase at the end of the reaction and solidified at ambient conditions typically revealed one or more intense endothermic peaks at temperatures lower than the reaction temperature, which corresponded to the melting of the maleimide-enriched phases (Figures S10 and S12).

Thus, the direct visual observations and DSC analysis revealed a significant correlation between the degrees of conversion and the content of the molten phase in the reaction mixtures. The retro-DA reaction for the furan–maleimide-derived adducts proceeded near their melting point, typically around 100 °C for the *endo*-adducts and above 110 °C for the *exo*-adducts (Figure S19) [66,67,68,84]. However, in solution, this process could take place at significantly lower temperatures [31,36,50,85]. The subsequent experiments were aimed at evaluating the potential of thermal decomposition of the studied *exo*-adducts in solution and in both molten and solid maleimide–adduct mixtures. For this purpose, we investigated the retro-DA reaction of pure adducts *exo*-**2**(**a**,**b**) in a DMSO-*d*_6_ solution, as well as in mechanical mixtures with three equivalents of unsubstituted maleimide or *N*-ethylmaleimide, after heating at 60–80 °C (Section S2.4 in the Supporting Information). Our observations indicated that *exo*-**2**(**a**,**b**) were unstable in DMSO-*d*_6_, undergoing a retro-DA reaction at 60 °C. At 80 °C, more than 95% of *exo*-**2**(**a**,**b**) decomposed into 2-furoic acid in DMSO-*d*_6_. The *exo*-**2b**/*N*-ethylmaleimide mixture, which formed a liquid phase upon heating at 60–80 °C, underwent a retro-DA reaction that resulted in the formation of 9 mol. % or 48 mol. % of 2-furoic acid at 60 °C or 80 °C, respectively. In contrast, the mechanical mixture of *exo*-**2a** with unsubstituted maleimide remained solid at 60–80 °C and did not undergo the retro-DA process.

These results indicate that the *exo*-adduct dissolved in a molten maleimide-enriched phase can undergo the retro-DA process at the reaction temperature (60–80 °C), but this process is inhibited in the solid phase. This also logically suggests that the mutual solubility of the reaction components (primarily maleimide and adduct) may influence the efficiency of the solvent-free fmDA reaction. The high solubility of the adduct in maleimide prevents its removal from the molten maleimide-enriched phase, allowing for the reversibility of the fmDA reaction and resulting in decreased conversion due to equilibrium limitations. Conversely, the limited solubility of the adduct in maleimide facilitates its removal from the molten maleimide-enriched phase through crystallization or precipitation during the fmDA reaction. As the reaction product accumulates, this leads to a significant reduction or even the complete absence of a molten maleimide-enriched phase containing the dissolved adduct, thereby reducing the contribution of the retro-DA process.

To validate this potential correlation between the solubility of the synthesized cycloadducts and their yield under neat conditions, we assessed the solubility of several *exo*-adducts in three organic solvents of medium polarity—in particular, ethyl acetate, tetrahydrofuran, and acetone (Table 4). Amides *exo*-**3**(**d**, **e**), as well as adducts *exo*-**1a** and *exo*-**2a**, obtained in high yields across a broad range of conditions, exhibited low solubility in all the solvents tested (Table 4, entries 1, 4, 7, 8). In contrast, adducts *exo*-**4**(**a**, **b**) and *exo*-**1e**, which did not precipitate from the reaction mixtures, along with *exo*-**1d** and *exo*-**2**(**b**, **e**), which required fine-tuning of the conditions for solidification of the reaction mixtures, demonstrated higher solubility in the solvents used (Table 4, entries 2, 3, 5, 6, 9, 10). This evaluation revealed a good correlation between the yields of fmDA adducts and their solubility. We assume that the low solubility of the *exo*-adducts (as proposed for adducts *exo*-**1a**, *exo*-**2a**,**c**, and *exo*-**3**(**a**–**e**)) ensures their relatively rapid precipitation from the molten maleimide-enriched phase during the fmDA reaction, even at moderate degrees of conversion. This phenomenon most likely explains the short reaction time and very high yield across a broad range of conditions observed for the fmDA reactions leading to these adducts. Unlike these low-soluble adducts, the solidification of more readily soluble adducts possibly occurs through continuous crystallization from the molten maleimide-enriched phase during the fmDA reaction (as proposed for adducts *exo*-**1**(**b**–**d**) and *exo*-**2**(**b**, **e**)). The crystallization is a slower and more intricate process than the precipitation, which explains the need to fine-tune the conditions for achieving high conversion values for these adducts (as in the experiments in Table 1, entries 6, 11, 17) and the reproducibility issues, as observed for adduct *exo*-**1d** (see Table 1, entries 17, 18). For adducts *exo*-**1e**, *exo*-**2b**, and *exo*-**4**(**a**–**d**), the removal from the molten phase via precipitation or crystallization does not occur under the tested conditions, which apparently explains the decreased conversion degrees limited by the equilibrium between the corresponding adduct and the starting substances in the reaction media.

In accordance with the theoretical and experimental results obtained, we conclude that the exceptional conversion and diastereoselectivity in solvent-free fmDA reactions with acceptor-substituted furans is a result of the continuous removal of the low-soluble *exo*-adducts formed from the melting reaction phase by precipitation or crystallization during the reaction. This process results in the solidification of the reaction mixture and shifts the reaction equilibrium towards the *exo*-adduct because of the absence of a molten phase necessary for the retro fmDA reaction. The mechanistic rationale behind such a liquid-to-solid transition of the reaction mixture is the possibility of the accumulation of the *exo*-adduct, which stems from a successful interplay between several factors. These factors highlight the uniqueness of this reaction system and include optimal melting points of the initial reactants, inherent stability and a high melting point of the reaction product (*exo*-adduct), and limited mutual solubility of the reaction components.

Initially, the presence of a maleimide substrate with a melting point lower than the optimal reaction temperature facilitates the formation of a molten phase necessary for the fmDA reaction to proceed. The relative thermodynamic stability of the formed *exo*-adduct allows for its accumulation in the reaction mixture. Subsequently, the final conversion achieved is influenced by the mutual solubility of the reaction components, particularly maleimide and the adduct. High mutual solubility allows the retro-DA reaction of the adduct in the molten maleimide-enriched phase, resulting in decreased conversion limited by the equilibrium. In contrast, the limited mutual solubility of the *exo*-adduct and the corresponding maleimide leads to the continuous removal of the *exo*-adduct from the molten maleimide-enriched phase through precipitation or crystallization.

A slight excess (1.1–1.5 eq., typically 1.25 eq.) of the maleimide dienophile helps drive the equilibrium towards the *exo*-adduct formation by maintaining a sufficient dienophile concentration as the adduct precipitates. This balance prevents both incomplete conversion (as was observed in some cases at lower than 1.1–1.25 eq. excesses of maleimide) and excessive accumulation of maleimide that could remain molten (as was observed in some cases at higher than 1.25–1.5 eq. excesses of maleimide). As a result, 1.1–1.25 eq. of maleimide provided the best compromise for complete solidification. Some furans (e.g., 2-furamide) that formed low-soluble DA adducts tolerated wider ranges (1.1–1.5 eq), while other substrates (e.g., methyl 2-furoate) that led to adducts with higher solubility required the precise 1.1–1.25 eq. for the most efficient crystallization/precipitation. This process leads to the further accumulation of the *exo*-adduct in the reaction medium during the reaction time, along with a decrease in the content of the molten phase. As a result, the reverse DA reaction is inhibited because of the phase transition of the reaction medium from a viscous liquid or melt formed by the starting substrates to a solid phase predominantly composed of the *exo*-adduct. The solidification of the reaction mixture, resulting from the continuous crystallization or precipitation of the formed *exo*-cycloadduct, shifts the reaction equilibrium towards this product. This phenomenon occurs because the melting point of the resulting solid adduct-enriched phase is higher than the temperature of the reaction. The designed solvent-free method is easily scalable, as demonstrated by a gram-scale experiment with the synthesis of adducts *exo*-**1a**, *exo*-**2a**, and *exo*-**2c**, resulting in identical conversion and yield (see Section S2.3 in the Supporting Information).

## 3. Materials and Methods 

### 3.1. General Information

Starting maleimides and furanic substrates were obtained by using published synthetic protocols [86,87] or purchased commercially (Shanghai Macklin Biochemical Technology Co., Ltd., Shanghai, China). Solvents were delivered from local suppliers.

Thermodynamic parameters of the reaction and its activation energy were calculated at the r2-SCAN-3c/Def2-TZVP level of theory [88,89,90] using CPCM nonspecific solvation model [91] with parameters of DMF solvent in ORCA program [92].

NMR spectra were recorded using a Bruker Fourier 300 HD and Bruker Avance II 300 spectrometers (both from Bruker Corporation, Billerica, MA, USA). The processing was carried out using the MestReNova software (version 12.0.0, Mestrelab Research SL, Santiago de Compostela, Spain). Solid-state NMR (ssNMR) experiments were recorded on a Bruker AVANCE III WB 400 MHz spectrometer (Bruker Corporation, Billerica, MA, USA).

HRMS spectra were recorded on a Bruker FT-ICR-MS solariX XR 15T mass-spectrometer or a Bruker maXis Q-TOF mass-spectrometer (both from Bruker Corporation, Billerica, MA, USA) equipped with an electrospray ionization (ESI) ion source.

DSC measurements were carried out on STA JUPITER 443 F3 NETZSCH or NETZH DSC 204 F1 Phoenix calorimeters (both from Netzsch GmbH, Germany).

X-ray diffraction data were collected at 100 K with a Bruker Quest D8 CMOS diffractometer (Bruker Corporation, Billerica, MA, USA), using graphite monochromatized Mo-Kα radiation (l = 0.71073 Å, ω-scans). The structure was solved using Intrinsic Phasing with the ShelXT [93] structure solution program in Olex2 [94] and refined with the XL [95] refinement package using Least-Squares minimization against F2 in the anisotropic approximation for non-hydrogen atoms. Positions of hydrogen atoms were calculated and then refined in the isotropic approximation within the riding model. Crystal data and structure refinement parameters are given in Table S8. CCDC 2393140 contains the supplementary crystallographic information for this paper.

Powder X-ray diffraction (PXRD) data were collected on a PROTO AXRD benchtop instrument (Proto AXRD Benchtop, Los Angeles, CA, USA) equipped with a Dectris Mythen 1K 1D-detector, using nickel-filtered CuKα (λ = 0.154056 Å) radiation, scanning range of approximately 3°-70° 2θ and scanning speed of 0.06° 2θ/s.

Melting process was studied in open glass capillaries on a MEL-TEMP® (Electrothermal, Rochford, UK) melting point apparatus.

### 3.2. General Procedure for the Optimization of the fmDA Reaction Conditions

A furanic substrate (0.1 mmol) and 1.1–3 eq. of the corresponding maleimide, with or without solvent, were placed in a 2 mL glass vial with a screw cap. Then, the vial was heated in an aluminum block at the appropriate temperature for the appropriate time. The resulting reaction mixtures were analyzed by ^1^H NMR, with PhSiMe_3_ as an internal standard, and the product ratios were determined by integration of the appropriate peaks. DSC data (Figures S1–S19) and selected ^1^H NMR spectra of the obtained reaction mixtures (Figures S22–S25) are given in the Supporting Information.

### 3.3. Typical Solvent-Free Protocol for Synthesis of Exo-Adducts

A furanic substrate (1 mmol) and 1.1–1.5 eq. of the appropriate maleimide were added to a 4 mL glass vial with a screw cap. Then, the vial was heated in an aluminum block at the appropriate temperature for the appropriate time. The reaction mixtures were washed with diethyl ether or ethyl acetate. The solid products *exo*-**1**(**a**–**e**), *exo*-**2**(**a**–**c**, **e**), *exo*-**3**(**a**–**c**), and *exo*-**4**(**a**,**b**) were obtained in 18–91% yields. The spectroscopic (NMR and ESI-MS) data of the synthesized compounds are given in the Supporting Information (Figures S31–S62 and Figures S66–S81).

### 3.4. Study of the Retro-DA Reaction

Pure *exo*-**2a**/**2b** (0.1 mmol) and 3 eq. of maleimide**/***N*-ethylmaleimide were placed in a 2 mL glass vial with a screw cap. Then, the vial was heated in an aluminum block at 60 °C or 80 °C for 1–3 days. In other experiments, pure *exo*-**2a**/**2b** (0.1 mmol) was dissolved in 0.6 mL of DMSO-*d*_6_ and heated in an aluminum bath at 60 °C or 80 °C for 1–3 days. The resulting reaction mixtures were analyzed using ^1^H NMR (see Figures S26–S30 in the Supporting Information). The obtained results are shown in Table S7.

## 4. Conclusions

Our study demonstrates that the fmDA reaction with challenging acceptor-substituted furans can be carried out with very high degrees of conversion and diastereoselectivity under neat conditions. We presume this phenomenon to be attributed to the spontaneous transition of the reaction medium from a melt formed by the starting substrates (furan and maleimide) at the beginning of the reaction to a solid phase enriched with the *exo*-adduct at the end of the reaction. The above-described results show that the choice of the reaction components with optimal melting points and limited mutual solubility, along with careful manipulation of the reaction conditions, may facilitate the solidification of the reaction mixtures via continuous crystallization or precipitation of the pure *exo*-adduct or adduct–maleimide mixtures over the course of the reaction, effectively preventing the reverse process. This liquid-to-solid phase transition may prove efficient in overcoming the reversibility problem, as it eliminates the liquid/molten phase necessary for the reverse process. The exceptionally high efficiency of this method in relation to the fmDA reaction of bio-based electron-poor furfural-derived furans provides significant advantages, allowing for a cleaner and more environmentally friendly process that avoids the challenges associated with the removal of solvent and side products. Our future research will focus on exploring the synthetic potential of the liquid-to-solid phase transition approach for other reversible processes, including the fmDA reaction with bio-derived donor-substituted furans.

## Figures and Tables

**Table 1:** Results of the DA reaction of methyl 2-furoate with maleimides in acetone and water and under neat conditions.

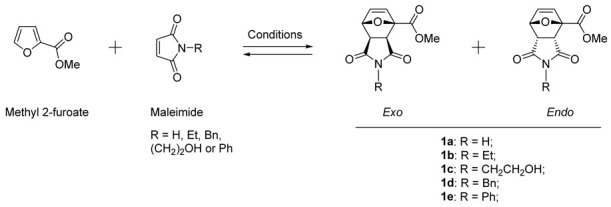							
№	R	Eq. of Maleimide	T, °C	Time, h	Solvent	Product (Initial Furan/*endo*/*exo* Ratio, %) ^1^	Aggregate State ^2^
1	H	1.5	60	24	Acetone	**1a** (92.5/0.5/7)	-
2	Et	1.5	60	24	Acetone	**1b** (98/trace/2)	-
3	H	1.5	60	24	H_2_O	**1a** (65/3/32)	-
4	Et	1.5	60	24	H_2_O	**1b** (61/4/35)	-
5	H	1.5	50	24	-	**1a** (42/1/57)	Solid + melt
6	H	1.1	60	24	-	**1a** (1/0/99)/87 ^3^	Solid
7	H	1.25	60	24	-	**1a** (15/0/85)	Solid + melt
8	H	1.25	60	72	-	**1a** (2/0/98)	Solid
9	H	1.5	60	24	-	**1a** (3/0/97)	Solid
10	H	1.5	80	24	-	**1a** (70/1/29)	Melt
11	Et	1.1	60	72	-	**1b** (1/0/99)/86 ^3^	Solid
12	Et	1.25	60	24	-	**1b** (18.5/0.5/81)	Melt
13	Et	1.25	60	72	-	**1b** (1/0/98)	Solid
14	Et	1.5	60	24	-	**1b** (39/2/59)	Melt
15	Et	1.5	60	72	-	**1b** (2/0/98)	Solid
16	Et	1.5	80	24	-	**1b** (58/2/40)	Melt
17	(CH_2_)_2_OH	1.25	60	72	-	**1c** (1/0/99)/79 ^3^	Solid
18	(CH_2_)_2_OH	1.25	60	72	-	**1c** (31/2/67)	Melt
19	Bn	1.25	60	72	-	**1d** (1/0/99)/91 ^3^	Solid
20	Ph	1.25	60	72	-	**1e** (21/1/78)/67 ^3^	Melt

Conditions: A mixture of 0.2 mmol of methyl 2-furoate and the corresponding amount of maleimide, with or without a solvent (1 mL), was placed in a 2 mL vial with a screw cap and maintained at the appropriate temperature for the appropriate time. ^1^ The resulting reaction mixtures were analyzed by ^1^H NMR with PhSiMe_3_ as an internal standard, and the product ratios were determined by integration of the appropriate peaks. ^2^ The aggregate state of the resulting reaction mixture under neat conditions at the reaction temperature. ^3^ Isolated yield. The maleimides used have the following melting points (m. p.): maleimide, 91–93 °C [73]; *N*-ethylmaleimide, 45 °C [74]; *N*-(2-hydroxyethyl)maleimide, 70–72 °C [75]; *N*-benzylmaleimide, 68–69 °C [76]; and *N*-phenylmaleimide, 89–90 °C [77]. Compounds *exo*-**1**(**a**–**e**) were isolated by washing the reaction mixture with diethyl ether.

**Table 2:** Results of the fmDA reactions of electron-poor furans with maleimides under neat conditions.

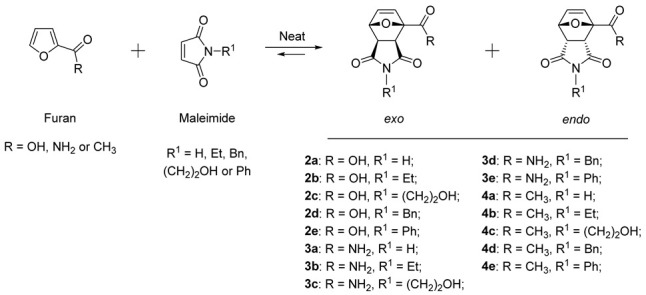						
№	R	R ^1^	Eq. of Maleimide	Conditions	Product (Initial Furan/*endo*/*exo* Ratio, %) ^1^	Aggregate State ^2^
1	OH	H	1.5	80 °C, 24 h	**2a** (1/0/99)/78 ^3^	Solid
2	OH	Et	1.25	60 °C, 24 h	**2b** (46/2/51)	Melt
3	OH	Et	1.25	60 °C, 72 h	**2b** (10/0/90)76 ^3^	Solid +melt
4	OH	Et	1.5	60 °C, 72 h	**2b** (42/2/55)	Melt
5	OH	Et	1.5	80 °C, 24 h	**2b** (65/2/32)	Melt
6	OH	(CH_2_)_2_OH	1.5	80 °C, 24 h	**2c** (2/0/98)/92 ^3^	Solid
7	OH	Bn	1.5	80 °C, 24 h	**2d** (60/0/40) ^4^	Melt
8	OH	Ph	1.25	80 °C, 24 h	**2e** (22/0/88)77 ^3^	Solid
9	NH_2_	H	1.1	80 °C, 24 h	**3a** (3/0/97)/85 ^3^	Solid
10	NH_2_	Et	1.25	80 °C, 24 h	**3b** (3/0/97)	Solid
11	NH_2_	Et	1.5	80 °C, 24 h	**3b** (1/0/99)/88 ^3^	Solid
12	NH_2_	Et	1.5	100 °C, 6 h	**3b** (2/0/98)	Solid
13	NH_2_	(CH_2_)_2_OH	1.25	80 °C, 24 h	**3c** (1/0/99)/87 ^3^	Solid
14	NH_2_	Bn	1.25	80 °C, 24 h	**3d** (1/0/99)/87 ^3^	Solid
15	NH_2_	Ph	1.25	80 °C, 24 h	**3e** (11/0/89)/81 ^3^	Solid
16	CH_3_	H	1.5	60 °C, 72 h	**4a** (76/1/23)/16 ^3^	Melt
17	CH_3_	Et	1.5	60 °C, 72 h	**4b** (72/2/26)/18 ^3^	Melt
18	CH_3_	(CH_2_)_2_OH	1.25	60 °C, 72 h	**4c** (66/1/33) ^4^	Melt
19	CH_3_	Bn	1.25	60 °C, 72 h	**4d** (67/1/32) ^4^	Melt
20	CH_3_	Ph	1.25	60 °C, 72 h	**4e** (85.5/0.5/14) ^4^	Melt

Conditions: A total of 0.2 mmol of furan and the corresponding amount of maleimide were placed in a 2 mL vial with a screw cap and kept at the appropriate temperature for the appropriate time. ^1^ The resulting reaction mixtures were analyzed by ^1^H NMR with PhSiMe_3_ as an internal standard, and the product ratios were determined by integration of the appropriate peaks. ^2^ The aggregate state of the resulting reaction mixture at the reaction temperature. ^3^ Isolated yield (%). ^4^ Not isolated from the reaction mixture. Compounds *exo*-**2**(**a**–**c**), *exo*-**2e**, and *exo*-**4**(**a**,**b**) were isolated by washing the reaction mixture with diethyl ether. Compounds *exo*-**4**(**a**–**e**) were isolated by washing the reaction mixture with ethyl acetate.

**Table 3:** Calculated thermodynamic parameters and activation energies for the fmDA reaction with methyl 2-furoate and 2-acetylfuran ^1^.

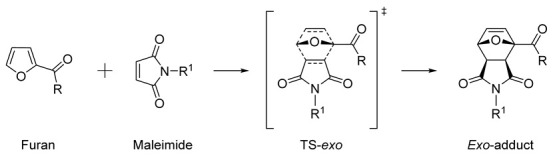				
№	Reaction Participant	ΔE, kcal/mol	ΔH^o^_298K_, kcal/mol	ΔG^o^_298K_, kcal/mol
1	Methyl 2-furoate	0.0	0.0	0.0
2	TS-*exo*-**1b**	10.5	10.0	25.2
3	*Exo*-**1b**	−8.0	−8.9	7.1
4	TS-*exo*-**1e**	10.3	9.9	25.3
5	*Exo*-**1e**	−7.1	−7.9	8.2
6	2-Acetylfuran	0.0	0.0	0.0
7	TS-*exo*-**4b**	11.5	11.0	25.9
8	*Exo*-**4b**	−7.1	−7.9	7.9
9	TS-*exo*-**4e**	10.7	10.3	25.5
10	*Exo*-**4e**	−6.1	−6.9	9.1

^1^ Calculated at the r2-SCAN-3c/Def2-TZVP level of theory using the CPCM nonspecific solvation model with parameters of the DMF solvent. R = OMe or CH_3_. R^1^ = Et or Ph. The symbol “‡” means transition state.

**Table 4:** Results of solubility assessment for the synthesized fmDA adducts in some organic solvents ^1^.

№	Adduct	Solubility in Ethyl Acetate	Solubility in Tetrahydrofuran	Solubility in Acetone
1	*exo*-**1a**	−	−	−
2	*exo*-**1d**	−	+	+
3	*exo*-**1e**	−	−	+
4	*exo*-**2a**	−	−	−
5	*exo*-**2b**	+	+	+
6	*exo*-**2e**	−	+	+
7	*exo*-**3d**	−	−	−
8	*exo*-**3e**	−	−	−
9	*exo*-**4a**	+	+	+
10	*exo*-**4b**	+	+	+

^1^ Solubility was assessed by stirring 5 mg of the corresponding *exo*-adduct in 200 µL of an appropriate solvent at 50 °C for 30 min. “+” means complete dissolution of the sample; “−” means the presence of undissolved residue.

## Data Availability

The data that support the findings of this study are available in the supplementary material of this article.

## Supplementary Materials

The supporting information can be downloaded at: https://www.mdpi.com/article/10.3390/ijms26146550/s1.

## Abbreviations

The following abbreviations are used in this manuscript:

DA	Diels–Alder
DFT	density functional theory
DMSO	dimethyl sulfoxide
DSC	differential scanning calorimetry
fmDA	furan–maleimide Diels–Alder
HEM	*N*-(2-hydroxyethyl)maleimide
HPM	*N*-(4-hydroxyphenyl)maleimide
NMR	nuclear magnetic resonance
PXRD	powder X-ray diffraction
ROMP	ring-opening metathesis polymerization
ssNMR	solid-state nuclear magnetic resonance
