# Supplementary material for: Overcoming the Reversibility in the Diels–Alder Reaction of Bio-Based Electron-Poor Furans with Maleimides Through Liquid-to-Solid Phase Transition
Source: Int J Mol Sci. 2025 Jul 8;26(14):6550. doi: 10.3390/ijms26146550 (PMC12294739; doi:10.3390/ijms26146550)
Supplement: Supplementary file 1 [file ijms-26-06550-s001.zip › ijms-3654121-supplementary_corrected.pdf]

Supplementary Information

Table of contents

1. General Information 2
2. Experimental Section..4
2.1. Optimization of the reaction conditions..4
2.2. Study of fmDA reaction with *N*-(4-hydroxyphenyl)maleimide under neat conditions........9
2.3. Synthesis of the *exo*-adducts of electron-poor furans with maleimides 11
2.4. Study of the retro-DA reaction 18
3. DSC experiments...19
4. X-ray diffraction data 38
5. NMR spectra...41
5.1. Selected NMR spectra of the reaction mixtures 41
5.2. NMR spectra of the retro-DA reactions...45
5.3. NMR spectra of the isolated *exo*-adducts 50
5.4. Solid-state NMR spectra...82
6. LCMS spectra...85
7. Optimized xyz-Cartesian coordinates...93
8. References 106

1. General Information

Starting maleimides and furanic substrates were obtained by using published synthetic protocols [1, 2] or purchased commercially (Shanghai Macklin Biochemical Technology Co., Ltd., Shanghai, China). Solvents were delivered from local suppliers. Reagents and solvents were of analytical grade or were purified prior to use by standard methods.

Thermodynamic parameters of the reaction and its activation energy were calculated at the r2-SCAN-3c/Def2-TZVP level of theory [3-5] using CPCM nonspecific solvation model [6] with parameters of DMF solvent in ORCA program [7].

NMR spectra were recorded using a Bruker Fourier 300 HD and Bruker Avance II 300 spectrometers (both from Bruker Corporation, Billerica, MA, USA) at the following frequencies: 300 MHz (^{1}H) and 75 MHz (^{13}C). The processing was carried out using the MestReNova software (version 12.0.0, Mestrelab Research SL, Santiago de Compostela, Spain). NMR chemical shifts were measured relative to residual protio solvent peaks. The following abbreviations are used: s = singlet, d = doublet, t = triplet, q = quartet, m = multiplet, br = broad. Solid-state NMR (ssNMR) experiments were recorded on a Bruker AVANCE III WB 400 MHz spectrometer (Bruker Corporation, Billerica, MA, USA) equipped with 4.0 mm DVT MAS BB/HF probe (15 kHz) (^{1}H – 400.1 MHz, ^{13}C – 100.6 MHz, ^{15}N – 40.6 MHz). Samples were spun at 9–14 kHz at the magic angle (MAS) using ZrO_2 rotors. ^{1}H MAS spectra were recorded using single-pulse sequence with 30° pulse at 14 kHz with a recycle delay of 8 sec. ^{13}C-CP/MAS spectra were recorded with a recycle delay of 8 sec and contact time of 4 msec at 12 kHz. ^{15}N-CP/MAS spectra were recorded with a recycle delay of 10 sec and contact time of 9 msec at 9 kHz. The ^{13}C and ^{15}N spectra were recorded under high-power proton decoupling conditions using "spinal64". Chemical shifts for ^{1}H and ^{13}C are relative to external adamantane sample; ^{15}N chemical shifts were calculated to this scale and were checked using ^{15}N-labelled-glycine sample. Magic angle was calibrated precisely to the spinning side bands in ^{81}Br spectra of the KBr sample.

HRMS spectra were recorded on a Bruker FT-ICR-MS solariX XR 15T mass-spectrometer or a Bruker maXis Q-TOF mass-spectrometer (both from Bruker Corporation, Billerica, MA, USA) equipped with an electrospray ionization (ESI) ion source.

DSC measurements were carried out on STA JUPITER 443 F3 NETZSCH calorimeter within a temperature range of 30 to 180 °C with heating rate of 10K min-1 or NETZH DSC 204 F1 Phoenix calorimeter (both from Netzsch GmbH, Germany) within a temperature range of 0 to 200 °C with heating rate of 2K min-1 in argon atmosphere.

X-ray diffraction data for *exo*-**1b** (the crystals of *exo*-**1b** obtained from the reaction mixture (conditions from Table S2, entry 12) were collected at 100 K with a Bruker Quest D8 CMOS

diffractometer (Bruker Corporation, Billerica, MA, USA), using graphite monochromatized Mo-Kα radiation (λ = 0.71073 Å, ω-scans). The structure was solved using Intrinsic Phasing with the ShelXT [8] structure solution program in Olex2 [9] and refined with the XL [10] refinement package using Least-Squares minimization against F2 in the anisotropic approximation for non-hydrogen atoms. Positions of hydrogen atoms were calculated and then refined in the isotropic approximation within the riding model. Crystal data and structure refinement parameters are given in Table S8. CCDC **2393140** contains the supplementary crystallographic information for this paper.

Powder X-ray diffraction (PXRD) data were collected on a PROTO AXRD benchtop instrument (Proto AXRD Benchtop, Los Angeles, CA, USA) equipped with a Dectris Mythen 1K 1D-detector, using nickel-filtered CuKα (λ = 0.154056 Å) radiation, scanning range of approximately 3°-70° 2θ and scanning speed of 0.06° 2θ/s.

Melting process was studied in open glass capillaries on a MEL-TEMP® (Electrothermal, Rochford, UK) melting point apparatus.

2. Experimental Section

2.1. Optimization of the reaction conditions

General procedure:

A furanic substrate (0.1 mmol) and 1.1-3 eq. of the corresponding maleimide or other dienophile, with or without solvent, were placed in a 2 mL glass vial with a screw cap. Then the vial was heated in an aluminum block at the appropriate temperature for the appropriate time. The resulting reaction mixtures were analyzed by ^{1}H NMR with $PhSiMe_3$ as an internal standard, and the product ratios were determined by integration of appropriate peaks. Selected NMR spectra of the reaction mixtures are given in Section 5.1.

Table S1. Results of the DA reactions of methyl 2-furoate with various alkenes in organic solvents.

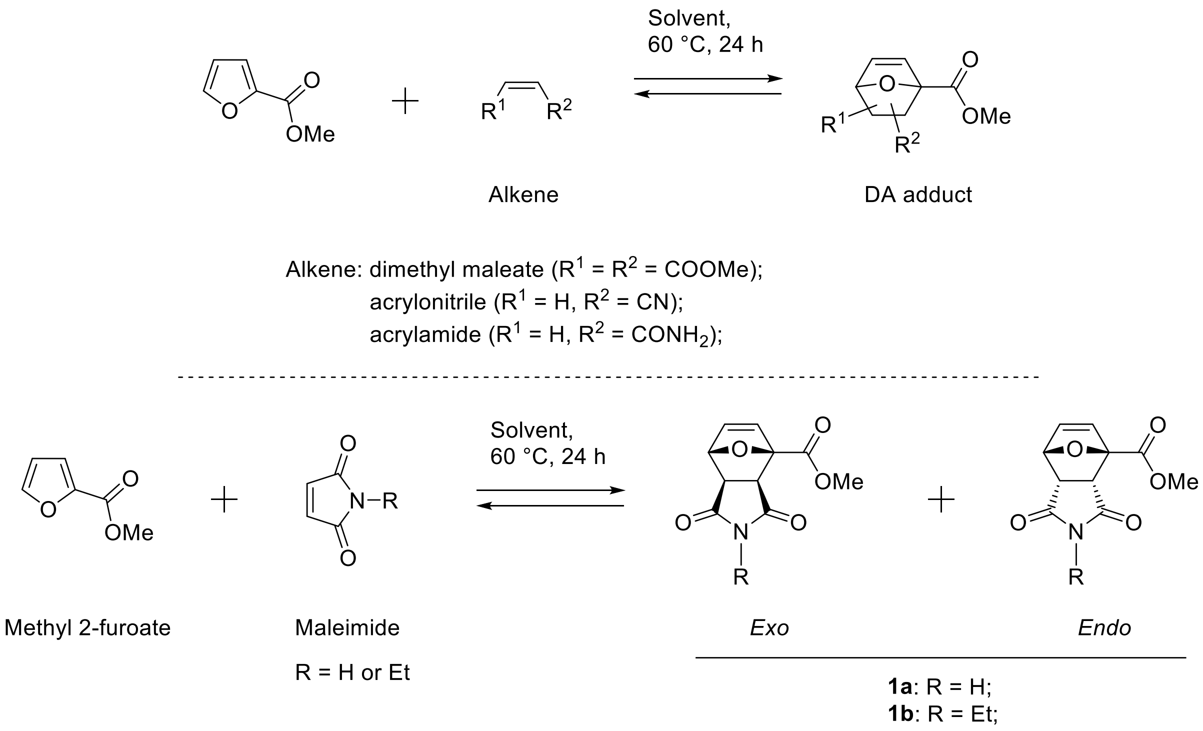

№	Alkene	Solvent	Products (initial furan/*endo*/*exo* ratio, %)
1	Acrylamide	Acetone or H_2O or neat conditions	No reaction
2	Acrylonitrile	Acetone or H_2O or neat conditions	No reaction

3	Dimethyl maleate	Acetone or H_2O or neat conditions	No reaction
4	Maleimide	Acetone	**1a** (92.5/0.5/7)
5	*N*-ethylmaleimide	Acetone	**1b** (98/trace/2)
6	Maleimide	H_2O	**1a** (65/3/32)
7	*N*-ethylmaleimide	H_2O	**1b** (61/4/35)

Table S2. Results of the *fm*DA reactions of methyl 2-furoate under neat conditions.

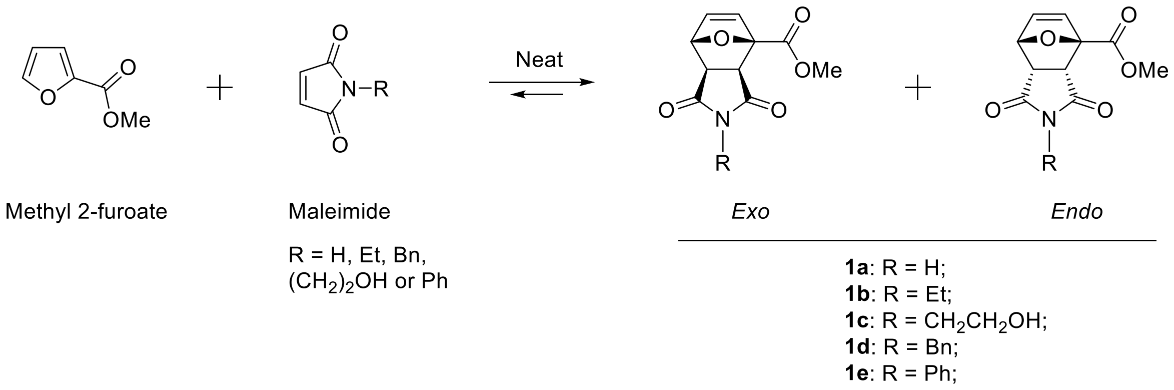

№	R[1]	Eq. of maleimide	T, °C	Time, h	Product (initial furan/*endo*/ *exo* ratio, %)	Aggregate state [1]
1	H	1.5	50	24	**1a** (42/1/57)	Solid + melt
2	H	1.1	60	24	**1a** (1/0/99)	Solid
3	H	1.25	60	24	**1a** (11/0/89)	Solid + melt
4	H	1.25	60	72	**1a** (2/0/98)	Solid
5	H	1.5	60	24	**1a** (3/0/97)	Solid
6	H	1.5	80	24	**1a** (70/1/29)	Melt
7	Et	1.5	40	72	**1b** (10/1/79)	Solid + melt
8	Et	1.1	60	72	**1b** (1/0/99)	Solid
9	Et	1.25	60	24	**1b** (18.5/0.5/81)	Melt
10	Et	1.25	60	72	**1b** (2/0/98)	Solid
11	Et	1.5	60	24	**1b** (39/2/59)	Melt

12	Et	1.5	60	72	**1b** (2/0/98)	Solid
13	Et	1.5	80	24	**1b** (58/2/40)	Melt
14	$(CH_2)_2OH$	1.25	60	72	**1c** (2/0/98)	Solid
15	$(CH_2)_2OH$	1.25	60	72	**1c** (31/2/67)	Melt
16	$(CH_2)_2OH$	1.5	60	24	**1c** (22/2/76)	Melt
17	$(CH_2)_2OH$	1.5	60	72	**1c** (1/0/99)	Solid
18	Bn	1.25	60	72	**1d** (1/0/99)	Solid
19	Bn	1.5	60	72	**1d** (1/0/99)	Solid
20	Ph	1.25	60	72	**1e** (21/1/78)	Melt
21	Ph	1.5	60	24	**1e** (57/2/41)	Melt
22	Ph	1.5	60	72	**1e** (26/1/73)	Melt

[1] The aggregate state of the resulting reaction mixture at the reaction temperature.

Table S3. Results of the *fm*DA reactions of 2-furoic acid under neat conditions.

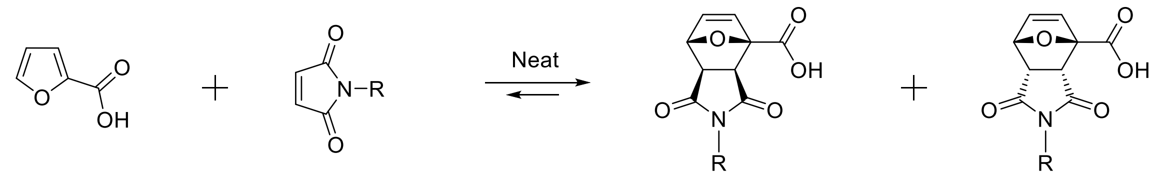

2-Furoic acid | Maleimide | *Exo* | *Endo*

R = H, Et, Bn, $(CH_2)_2OH$ or Ph

2a: R = H;
2b: R = Et;
2c: R = CH_2CH_2OH;
2d: R = Bn;
2e: R = Ph;

№	R[1]	Eq. of maleimide	Conditions	Product (initial furan/*endo*/ *exo* ratio, %)	Aggregate state [1]
1	H	1.1	80 °C, 24 h	**2a** (2/0/98)	Solid
2	H	1.25	80 °C, 24 h	**2a** (2/0/98)	Solid
3	H	1.5	80 °C, 30 min	**2a** (66/0/34)	Solid +melt
4	H	1.5	80 °C, 1 h	**2a** (36/0/64)	Melt
5	H	1.5	80 °C, 2 h	**2a** (13/0/87)	Solid

6	H	1.5	80 °C, 4 h	**2a** (4/0/96)	Solid
7	H	1.5	80 °C, 24 h	**2a** (1/0/99)	Solid
8	H	1.75	80 °C, 6 h	**2a** (5/0/95)	Solid
9	H	2	80 °C, 6 h	**2a** (7/0/93)	Solid
10	H	3	80 °C, 24 h	**2a** (6/0/94)	Solid
11	H	1.5	120 °C, 6 h	**2a** (4/0/96)	Solid
12	Et	1.25	60 °C, 24 h	**2b** (46/2/51)	Melt
13	Et	1.25	60 °C, 72 h	**2b** (10/0/90)	Solid +melt
14	Et	1.5	60 °C, 72 h	**2b** (42/2/55)	Melt
15	Et	1.5	60 °C, 48 h, then 40 °C, 72 h	**2b** (10/0/90)	Solid +melt
16	Et	1.5	80 °C, 24 h	**2b** (65/2/32)	Melt
17	$(CH_2)_2OH$	1.25	80 °C, 72 h	**2c** (4/0/96)	Solid
18	$(CH_2)_2OH$	1.5	80 °C, 30 min	**2c** (50/2/48)	Melt
19	$(CH_2)_2OH$	1.5	80 °C, 1 h	**2c** (24/2/74)	Solid +melt
20	$(CH_2)_2OH$	1.5	80 °C, 2 h	**2c** (17/1/82)	Solid
21	$(CH_2)_2OH$	1.5	80 °C, 24 h	**2c** (2/0/98)	Solid
22	$(CH_2)_2OH$	3	80 °C, 24 h	**2c** (7/0/93)	Solid
23	Bn	1.25	80 °C, 24 h	**2d** (62/0/38)	Melt
24	Bn	1.5	80 °C, 24 h	**2d** (60/0/40)	Melt
25	Ph	1.25	80 °C, 24 h	**2e** (12/0/88)	Solid
26	Ph	1.5	80 °C, 24 h	**2e** (79/0/21)	Solid + melt
27	Ph	1.5	100 °C, 24 h	**2e** (31/0/69)	Solid + melt

[1] The aggregate state of the resulting reaction mixture at the reaction temperature.

Table S4. Results of the *fm*DA reactions of 2-furamide under neat conditions.

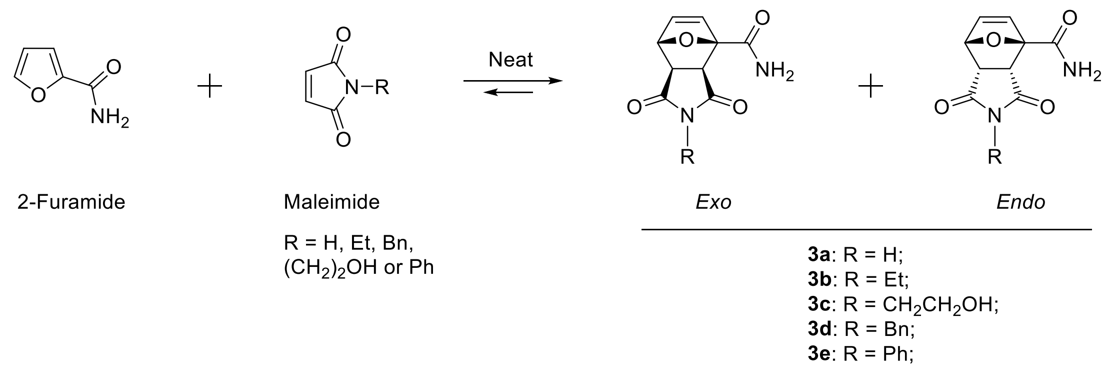

№	R[1]	Eq. of maleimide	Conditions	Product (initial furan/*endo*/*exo* ratio, %)	Aggregate state [1]
1	H	1.1	80 °C, 24 h	**3a** (3/0/97)	Solid
2	H	1.25	80 °C, 24 h	**3a** (3/0/97)	Solid
3	H	1.5	80 °C, 2 h	**3a** (17/0/83)	Solid
4	H	1.5	80 °C, 24 h	**3a** (4/0/96)	Solid
5	H	1.5	100 °C, 6 h	**3a** (3/0/97)	Solid
6	Et	1.25	80 °C, 24 h	**3b** (3/0/97)	Solid
7	Et	1.5	60 °C, 24 h	**3b** (10/6/84)	Solid +melt
8	Et	1.5	80 °C, 24 h	**3b** (1/0/99)	Solid
9	Et	1.5	80 °C, 2 h	**3b** (64/0/36)	Solid +melt
10	Et	1.5	100 °C, 6 h	**3b** (2/0/98)	Solid
11	$(CH_2)_2OH$	1.1	80 °C, 24 h	**3c** (15/0/85)	Solid
12	$(CH_2)_2OH$	1.25	80 °C, 24 h	**3c** (1/0/99)	Solid
13	$(CH_2)_2OH$	1.5	80 °C, 24 h	**3c** (1/0/99)	Solid
14	$(CH_2)_2OH$	1.5	100 °C, 6 h	**3c** (1/0/99)	Solid
15	Bn	1.1	80 °C, 24 h	**3d** (24/0/76)	Solid
16	Bn	1.25	80 °C, 24 h	**3d** (1/0/99)	Solid
17	Bn	1.5	80 °C, 24 h	**3d** (1/0/99)	Solid
18	Bn	1.5	100 °C, 6 h	**3d** (1/0/99)	Solid

19	Ph	1.25	80 °C, 24 h	**3e** (11/0/89)	Solid
20	Ph	1.5	100 °C, 6 h	**3d** (13/0/87)	Solid

[1] The aggregate state of the resulting reaction mixture at the reaction temperature.

Table S5. Results of the *fm*DA reactions of 2-acetylfuran under neat conditions.

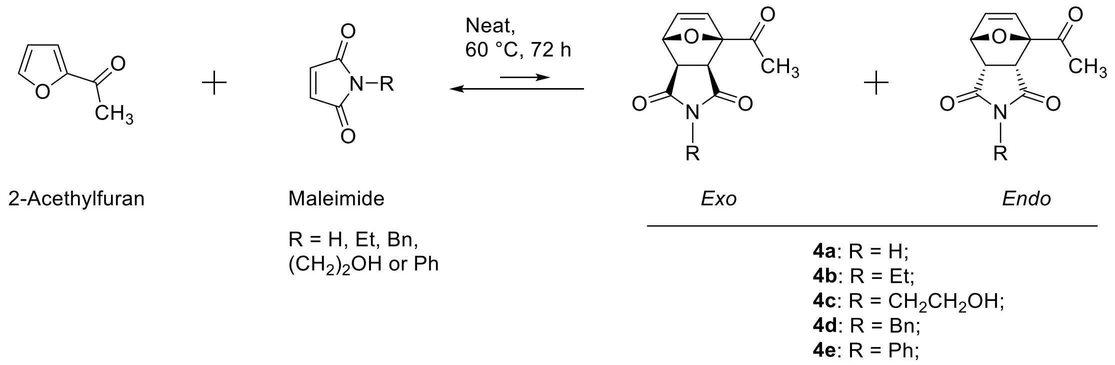

№	R[1]	Eq. of maleimide	Product (initial furan/ *endo*/*exo* ratio, %)	Aggregate state [1]
1	H	1.5	**4a** (76/1/23)	Melt
2	Et	1.5	**4b** (72/2/26)	Melt
3	$(CH_2)_2OH$	1.25	**4c** (66/1/33)	Melt
4	Bn	1.25	**4d** (67/1/32)	Melt
5	Ph	1.25	**4e** (85.5/0.5/14)	Melt

[1] The aggregate state of the resulting reaction mixture at the reaction temperature.

2.2. Study of fmDA reaction with *N*-(4-hydroxyphenyl)maleimide under neat conditions

A furanic substrate (0.1 mmol) and the corresponding amount of *N*-(4-hydroxyphenyl)maleimide (HPM) were placed in a 2 mL glass vial with a screw cap. Then the vial was heated in an aluminum block at the appropriate temperature for the appropriate time. The resulting reaction mixtures were analyzed by ^{1}H NMR with $PhSiMe_3$ as an internal standard, and the product ratios were determined by integration of appropriate peaks.

Table S6. Results of the *fm*DA reactions with HPM under neat conditions.

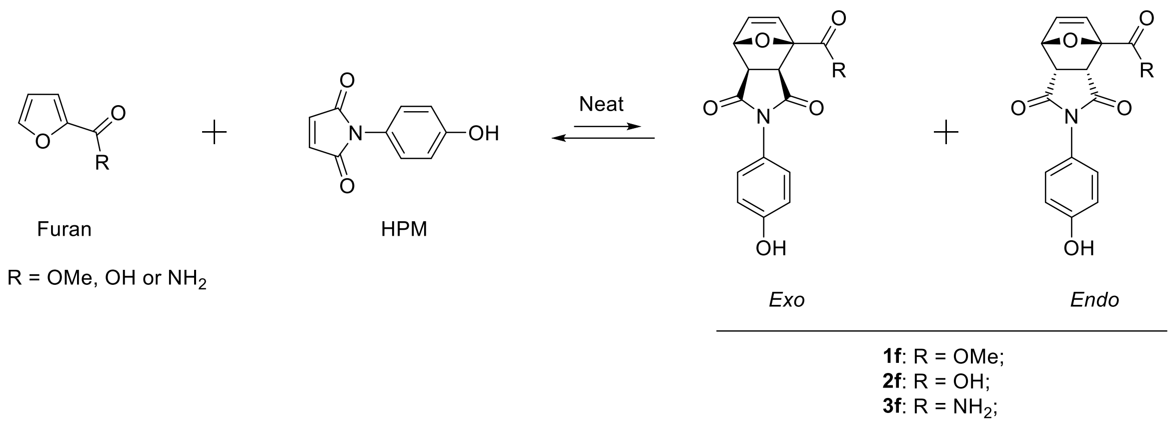

№	R	Eq. of HPM	Conditions	Conversion [1]	Product yield (*endo*/*exo*, %)	Aggregate state [1]
1	OMe	1.25	80 °C, 24 h	1	**1f** (0/1)	Solid [2]
2	OMe	1.25	120 °C, 24 h	2	**1f** (0/1.5)	Solid [2]
3	OMe	1.25	140 °C, 24 h	3	**1f** (0/2)	Solid [2]
4	OMe	1.25	160 °C, 24 h	10	**1f** (0/5)	Melt
5	OH	1.25	160 °C, 24 h	15	**2f** (0/10)	Melt
6	NH_2	1.1	160 °C, 1 h	24	**3f** (1/11)	Melt
7	NH_2	1.25	160 °C, 1 h	30	**3f** (2/17)	Melt
8	NH_2	1.5	160 °C, 1 h	42	**3f** (2/19)	Melt
9	OMe	0.75	160 °C, 1 h	22 [3]	**1f** (1.5/8)	Melt
10	OH	0.75	160 °C, 1 h	24 [3]	**2f** (3/12)	Melt
11	NH_2	0.75	160 °C, 1 h	30 [3]	**3f** (8/14)	Melt

[1] The aggregate state of the resulting reaction mixture at the reaction temperature. [2] HPM did not melt during the reaction and remained in a solid state. [3] Estimated relative to HPM.

2.3. Synthesis of the *exo*-adducts of electron-poor furans with maleimides

Synthesis of *exo*-1a.

The mixture of methyl 2-furoate (107 µL, 1 mmol) and maleimide (107 mg, 1.1 mmol) was heated in a 4 mL glass vial with a screw cap in an aluminum block at 60 °C for 24 hours. The solid residue was washed with diethyl ether (Et_2O), yielding 194 mg (87% yield) of product ***exo*-1a** as a white precipitate. The same procedure was carried out using 10 mmol of methyl 2-furoate in a 10 mL round-bottom flask, resulting in identical conversion and yield of *exo*-**1a**.

Methyl *exo*-1,3-dioxo-1,2,3,3a,7,7a-hexahydro-4H-4,7-epoxyisoindole-4-carboxylate (*exo*-1a) [11].

^{1}H NMR (300 MHz, DMSO-d_6) δ 11.30 (br. s, 1H), 6.70 (dd, *J* = 5.6, 1.8 Hz, 1H), 6.58 (d, *J* = 5.6 Hz, 1H), 5.22 (d, *J* = 1.8 Hz, 1H), 3.78 (s, 3H), 3.16 (d, *J* = 6.5 Hz, 1H), 3.03 (d, *J* = 6.5 Hz, 1H). ^{13}C{^{1}H} NMR (75 MHz, DMSO-d_6) δ 177.3, 175.8, 167.5, 138.6, 136.2, 89.1, 81.2, 52.8, 52.0, 50.2. m/z HRMS (ESI) Calcd. for $C_{10}H_9NO_5$ [M + Na]: 246.0373. Found 246.0374.

Synthesis of *exo*-1b.

The mixture of methyl 2-furoate (107 µL, 1 mmol) and *N*-ethyl maleimide (138 mg, 1.1 mmol) was heated in a 4 mL glass vial with a screw cap in an aluminum block at 60 °C for 3 days hours. The solid residue was washed with Et_2O, yielding 216 mg (86% yield) of product *exo*-**1b** as a white precipitate.

Methyl *exo*-2-ethyl-1,3-dioxo-1,2,3,3a,7,7a-hexahydro-4H-4,7-epoxyisoindole-4-carboxylate (*exo*-1b) [11].

^{1}H NMR (300 MHz, DMSO-d_6) δ 6.72 (dd, *J* = 5.6, 1.8 Hz, 1H), 6.61 (d, *J* = 5.6 Hz, 1H), 5.24 (d, *J* = 1.8 Hz, 1H), 3.79 (s, 3H), 3.36 (q, *J* = 7.2 Hz, 2H, partially overlapped with residual water signal), 3.21 (d, *J* = 6.5 Hz, 1H), 3.09 (d, *J* = 6.5 Hz, 1H), 1.01 (t, *J* = 7.2 Hz, 3H). ^{13}C{^{1}H} NMR (75 MHz, DMSO-d_6) δ 175.8, 174.3, 167.4, 138.6, 136.2, 89.0, 81.2, 52.8, 50.7, 48.9, 33.5, 13.1. m/z HRMS (ESI) Calcd. for $C_{12}H_{13}NO_5$ [M + H]: 252.0866. Found 252.0875.

Synthesis of *exo*-1c.

The mixture of methyl 2-furoate (107 µL, 1 mmol) and *N*-hydroxyethyl maleimide (212 mg, 1.5 mmol) was heated in a 4 mL glass vial with a screw cap in an aluminum bath at 60 °C for 3

days hours. The solid residue was washed with Et_2O, yielding 216 mg (79% yield) of product *exo*-**1c** as a white precipitate.

Methyl *exo*-2-(2-hydroxyethyl)-1,3-dioxo-1,2,3,3a,7,7a-hexahydro-4H-4,7-epoxyisoindole-4-carboxylate (*exo*-1c).

^{1}H NMR (400 MHz, DMSO-d_6) δ 6.72 (br. d, *J* = 5.6 Hz, 1H), 6.61 (d, *J* = 5.6 Hz, 1H), 5.24 (br. s, 1H), 4.81 (m, 1H), 3.79 (s, 3H), 3.40 (br. s, 4H), 3.24 (d, *J* = 6.5 Hz, 1H), 3.12 (d, *J* = 6.5 Hz, 1H). ^{13}C{^{1}H} NMR (75 MHz, DMSO-d_6) δ 176.2, 174.7, 167.5, 138.5, 136.1, 89.0, 81.1, 57.5, 52.9, 50.6, 48.8, 41.1. m/z HRMS (ESI) Calcd. for $C_{12}H_{13}NO_6$ [M + H]: 268.0816. Found 268.0820.

Synthesis of *exo*-1d.

The mixture of methyl 2-furoate (107 µL, 1 mmol) and *N*-benzyl maleimide (234 mg, 1.25 mmol) was heated in a 4 mL glass vial with a screw cap in an aluminum block at 60 °C for 3 days hours. The solid residue was washed with Et_2O, yielding 284 mg (91% yield) of product *exo*-**1d** as a white precipitate.

Methyl *exo*-2-benzyl-1,3-dioxo-1,2,3,3a,7,7a-hexahydro-4H-4,7-epoxyisoindole-4-carboxylate (*exo*-1d).

^{1}H NMR (300 MHz, DMSO-d_6) δ 7.38 – 7.17 (m, 5H), 6.74 (m, 1H), 6.65 (d, *J* = 5.5 Hz, 1H), 5.29 (br. s, 1H), 4.60 (d, *J* = 15.3 Hz, 1H), 4.53 (d, *J* = 15.3 Hz, 1H), 3.77 (s, 3H), 3.32 (d, *J* = 6.5 Hz, 1H, partially overlapped with residual water signal), 3.19 (d, *J* = 6.5 Hz, 1H). ^{13}C{^{1}H} NMR (75 MHz, DMSO-d_6) δ 175.8, 174.4, 167.3, 138.6, 136.2, 136.1, 128.9, 127.8, 127.4, 89.2, 81.2, 52.8, 50.9, 49.0, 41.9. m/z HRMS (ESI) Calcd. for $C_{17}H_{15}NO_5$ [M + Na]: 336.08424. Found 336.08414.

Synthesis of *exo*-1e.

The mixture of methyl 2-furoate (107 µL, 1 mmol) and *N*-phenyl maleimide (217 mg, 1.25 mmol) was heated in a 4 mL glass vial with a screw cap in an aluminum block at 60 °C for 3 days hours. The resulting viscous liquid was washed with Et_2O, yielding 201 mg (67% yield) of product *exo*-**1e** as a white precipitate.

Methyl *exo*-1,3-dioxo-2-phenyl-1,2,3,3a,7,7a-hexahydro-4H-4,7-epoxyisoindole-4-carboxylate (*exo*-1e).

^{1}H NMR (300 MHz, DMSO-d_6) δ 7.55 – 7.40 (m, 3H), 7.20 (m, 2H), 6.78 (dd, *J* = 5.6, 1.7 Hz, 1H), 6.68 (d, *J* = 5.6 Hz, 1H), 5.37 (d, *J* = 1.7 Hz, 1H), 3.80 (s, 3H), 3.38 (d, *J* = 6.5 Hz, 1H), 3.28 (d, *J* = 6.5 Hz, 1H). ^{13}C{^{1}H} NMR (75 MHz, DMSO-d_6) δ 175.2, 173.7, 167.4, 138.7, 136.3, 132.4, 129.5, 129.0, 127.1, 89.5, 81.6, 52.9, 51.0, 49.2. m/z HRMS (ESI) Calcd. for $C_{16}H_{13}NO_5$ [M + H]: 300.0866. Found 300.0864.

Synthesis of *exo*-2a.

The mixture of 2-furoic acid (112 mg, 1 mmol) and maleimide (146 mg, 1.5 mmol) was heated in a 4 mL glass vial with a screw cap in an aluminum block at 60 °C for 24 hours. The solid residue was washed with Et_2O, yielding 164 mg (78% yield) of product *exo*-**2a** as a white precipitate. The same procedure was carried out using 20 mmol of 2-furoic acid in a 25 mL round-bottom flask, resulting in identical conversion and yield of *exo*-**2a**.

***Exo*-1,3-dioxo-1,2,3,3a,7,7a-hexahydro-4H-4,7-epoxyisoindole-4-carboxylic acid (*exo*-2a) [11].**

^{1}H NMR (300 MHz, DMSO-d_6) δ 13.48 (br. s, 1H), 11.24 (br. s, 1H), 6.66 (dd, *J* = 5.5, 1.7 Hz, 1H), 6.55 (d, *J* = 5.5 Hz, 1H), 5.17 (d, *J* = 1.7 Hz, 1H), 3.10 (d, *J* = 6.5 Hz, 1H), 3.01 (d, *J* = 6.5 Hz, 1H). ^{13}C{^{1}H} NMR (75 MHz, DMSO-d_6) δ 177.5, 175.8, 168.4, 138.3, 136.8, 89.2, 81.0, 51.5, 50.3. m/z HRMS (ESI) Calcd. for $C_9H_7NO_5$ [M + Na]: 232.0216. Found 232.0214.

Synthesis of *exo*-2b.

The mixture of 2-furoic acid (112 mg, 1 mmol) and *N*-ethyl maleimide (156 mg, 1.25 mmol) was heated in a 4 mL glass vial with a screw cap in an aluminum block at 60 °C for 3 days hours. The residue was washed with Et_2O, yielding 180 mg (76% yield) of product *exo*-**2b** as a white precipitate.

***Exo*-2-ethyl-1,3-dioxo-1,2,3,3a,7,7a-hexahydro-4H-4,7-epoxyisoindole-4-carboxylic acid (*exo*-2b).**

^{1}H NMR (300 MHz, DMSO-d_6) δ 13.52 (br. s, 1H), 6.69 (dd, *J* = 5.6, 1.8 Hz, 1H), 6.58 (d, *J* = 5.6 Hz, 1H), 5.19 (d, *J* = 1.8 Hz, 1H), 3.37 (q, *J* = 7.2 Hz, 2H, overlapped with residual water signal), 3.16 (d, *J* = 6.5 Hz, 1H), 3.07 (d, *J* = 6.5 Hz, 1H), 1.02 (t, *J* = 7.2 Hz, 3H). ^{13}C{^{1}H} NMR (75 MHz, DMSO-d_6) δ 175.9, 174.3, 168.2, 138.3, 136.8, 89.1, 81.0, 50.3, 49.0, 33.5, 13.2. m/z HRMS (ESI) Calcd. for $C_{11}H_{11}NO_5$ [M + Na]: 260.0529. Found 260.0527.

Synthesis of *exo*-2c.

The mixture of 2-furoic acid (112 mg, 1 mmol) and *N*-hydroxyethyl maleimide (212 mg, 1.5 mmol) was heated in a 4 mL glass vial with a screw cap in an aluminum block at 60 °C for 3 days hours. The solid residue was washed with Et_2O, yielding 233 mg (92% yield) of product *exo*-**2c** as a white precipitate. The same procedure was carried out using 10 mmol of 2-furoic acid in a 10 mL round-bottom flask, resulting in identical conversion and yield of *exo*-**2c**.

***Exo*-2-(2-hydroxyethyl)-1,3-dioxo-1,2,3,3a,7,7a-hexahydro-4H-4,7-epoxyisoindole-4-carboxylic acid (*exo*-2c).**

^{1}H NMR (300 MHz, DMSO-d_6) δ 13.52 (br. s, 1H), 6.68 (dd, *J* = 5.5, 1.6 Hz, 1H), 6.58 (d, *J* = 5.5 Hz, 1H), 5.19 (d, *J* = 1.6 Hz, 1H), 4.77 (br. s, 1H), 3.41 (br. s, 4H), 3.18 (d, *J* = 6.5 Hz, 1H), 3.08 (d, *J* = 6.5 Hz, 1H). ^{13}C{^{1}H} NMR (75 MHz, DMSO-d_6) δ 176.1, 174.5, 168.3, 138.3, 136.8, 89.1, 81.0, 57.69, 50.2, 49.0, 41.2. m/z HRMS (ESI) Calcd. for $C_{11}H_{11}NO_6$ [M + Na]: 276.04786. Found 276.04795.

Synthesis of *exo*-2e.

The mixture of 2-furoic acid (112 mg, 1 mmol) and *N*-phenyl maleimide (217 mg, 1.25 mmol) was heated in a 4 mL glass vial with a screw cap in an aluminum block at 60 °C for 3 days hours. The solid residue was washed with Et_2O, yielding 201 mg (77% yield) of product *exo*-**2e** as a white precipitate.

***Exo*-1,3-dioxo-2-phenyl-1,2,3,3a,7,7a-hexahydro-4H-4,7-epoxyisoindole-4-carboxylic acid (*exo*-2e).**

^{1}H NMR (300 MHz, DMSO-d_6) δ 13.60 (br. s, 1H), 7.60 – 7.39 (m, 3H), 7.20 (m, 2H), 6.74 (dd, *J* = 5.6, 1.7 Hz, 1H), 6.66 (d, *J* = 5.6 Hz, 1H), 5.32 (d, *J* = 1.7 Hz, 1H), 3.32 (d, *J* = 6.5 Hz, 1H, overlapped with residual water signal), 3.25 (d, *J* = 6.5 Hz, 1H). ^{13}C{^{1}H} NMR (75 MHz, DMSO-d_6) δ 175.4, 173.7, 168.3, 138.5, 137.0, 132.5, 129.5, 129.0, 127.2, 89.6, 81.5, 50.6, 49.3. m/z HRMS (ESI) Calcd. for $C_{15}H_{11}NO_5$ [M + Na]: 308.05294. Found 308.05285.

Synthesis of *exo*-3a.

The mixture of 2-furamide (111 mg, 1 mmol) and maleimide (107 mg, 1.1 mmol) was heated in a 4 mL glass vial with a screw cap in an aluminum block at 60 °C for 24 hours. The solid residue was washed with ethyl acetate (EtOAc), yielding 176 mg (85% yield) of product *exo*-**3a** as a white precipitate.

***Exo*-1,3-dioxo-1,2,3,3a,7,7a-hexahydro-4H-4,7-epoxyisoindole-4-carboxamide (*exo*-3a).**

^{1}H NMR (300 MHz, DMSO-d_6) δ 11.19 (br. s, 1H), 7.42 (br. s, 1H), 7.28 (br. s, 1H), 6.63 (dd, *J* = 5.6, 1.7 Hz, 1H), 6.53 (d, *J* = 5.6 Hz, 1H), 5.19 (d, *J* = 1.7 Hz, 1H), 3.07 (d, *J* = 6.5 Hz, 1H), 3.01 (d, *J* = 6.5 Hz, 1H). ^{13}C{^{1}H} NMR (75 MHz, DMSO-d_6) δ 177.5, 175.4, 168.0, 137.6, 90.3, 81.1, 50.8, 50.6. m/z HRMS (ESI) Calcd. for $C_9H_8N_2O_4$ [M + Na]: 231.0376. Found 231.0376.

Synthesis of *exo*-3b.

The mixture of 2-furamide (111 mg, 1 mmol) and *N*-ethyl maleimide (188 mg, 1.5 mmol) was heated in a 4 mL glass vial with a screw cap in an aluminum block at 60 °C for 3 days. The solid residue was washed with EtOAc, yielding 207 mg (88% yield) of product *exo*-**3b** as a white precipitate.

***Exo*-2-ethyl-1,3-dioxo-1,2,3,3a,7,7a-hexahydro-4H-4,7-epoxyisoindole-4-carboxamide (*exo*-3b).**

^{1}H NMR (300 MHz, DMSO-d_6) δ 7.43 (br. s, 1H), 7.23 (br. s, 1H), 6.64 (dd, *J* = 5.6, 1.7 Hz, 1H), 6.56 (d, *J* = 5.6 Hz, 1H), 5.21 (d, *J* = 1.7 Hz, 1H), 3.36 (q, *J* = 7.2 Hz, 2H, overlapped with residual water signal), 3.12 (d, *J* = 6.5 Hz, 1H), 3.06 (d, *J* = 6.5 Hz, 1H), 1.03 (t, *J* = 7.2 Hz, 3H). ^{13}C{^{1}H} NMR (75 MHz, DMSO-d_6) δ 176.1, 173.9, 168.0, 137.63, 137.57, 90.3, 81.1, 49.6, 49.3, 33.5, 13.2. m/z HRMS (ESI) Calcd. for $C_{11}H_{12}N_2O_4$ [M + H]: 237.0870. Found 237.0867.

Synthesis of *exo*-3c.

The mixture of 2-furamide (111 mg, 1 mmol) and *N*-hydroxyethyl maleimide (176 mg, 1.25 mmol) was heated in a 4 mL glass vial with a screw cap in an aluminum block at 60 °C for 3 days hours. The solid residue was washed with EtOAc, yielding 220 mg (87% yield) of product *exo*-**3c** as a white precipitate.

***Exo*-2-(2-hydroxyethyl)-1,3-dioxo-1,2,3,3a,7,7a-hexahydro-4H-4,7-epoxyisoindole-4-carboxamide (*exo*-3c).**

^{1}H NMR (300 MHz, DMSO-d_6) δ 7.44 (br. s, 1H), 7.17 (br. s, 1H), 6.63 (dd, *J* = 5.5, 1.4 Hz, 1H), 6.57 (d, *J* = 5.5 Hz, 1H), 5.21 (d, *J* = 1.4 Hz, 1H), 4.78 (br. t, *J* = 5.5 Hz, 1H), 3.42 (m, 4H), 3.12 (d, *J* = 6.5 Hz, 1H), 3.08 (d, *J* = 6.5 Hz, 1H). ^{13}C{^{1}H} NMR (75 MHz, DMSO-d_6) δ 176.3, 174.1, 168.0, 137.54, 137.51, 90.3, 81.2, 57.7, 49.7, 49.3, 41.2. m/z HRMS (ESI) Calcd. for $C_{11}H_{12}N_2O_5$ [M + H]: 275.0638. Found 275.0641.

Synthesis of *exo*-3d.

The mixture of 2-furamide (111 mg, 1 mmol) and *N*-benzyl maleimide (234 mg, 1.25 mmol) was heated in a 4 mL glass vial with a screw cap in an aluminum block at 60 °C for 3 days hours. The solid residue was washed with EtOAc, yielding 260 mg (87% yield) of product *exo*-**3d** as a white precipitate.

***Exo*-2-benzyl-1,3-dioxo-1,2,3,3a,7,7a-hexahydro-4H-4,7-epoxyisoindole-4-carboxamide (*exo*-3d).**

^{1}H NMR (300 MHz, DMSO-d_6) δ 7.47 (br. s, 1H), 7.41 – 7.15 (m, 6H), 6.67 (dd, *J* = 5.5, 1.6 Hz, 1H), 6.59 (d, *J* = 5.5 Hz, 1H), 5.26 (d, *J* = 1.6 Hz, 1H), 4.58 (d, *J* = 3.4 Hz, 1H), 4.52 (d, *J* = 3.4 Hz, 1H), 3.23 (d, *J* = 6.4 Hz, 1H), 3.17 (d, *J* = 6.4 Hz, 1H). ^{13}C{^{1}H} NMR (75 MHz, DMSO-d_6) δ 176.1, 174.0, 167.9, 137.7, 136.3, 129.0, 127.7, 127.5, 90.4, 81.2, 49.7, 49.5, 41.9. m/z HRMS (ESI) Calcd. for $C_{16}H_{14}N_2O_4$ [M + Na]: 307.0689. Found 307.0689.

Synthesis of *exo*-3e.

The mixture of 2-furamide (111 mg, 1 mmol) and *N*-phenyl maleimide (217 mg, 1.25 mmol) was heated in a 4 mL glass vial with a screw cap in an aluminum block at 60 °C for 24 hours. The solid residue was washed with EtOAc, yielding 231 mg (81% yield) of product *exo*-**3e** as a white precipitate.

***Exo*-1,3-dioxo-2-phenyl-1,2,3,3a,7,7a-hexahydro-4H-4,7-epoxyisoindole-4-carboxamide (*exo*-3e).**

^{1}H NMR (300 MHz, DMSO-d_6) δ 7.55 – 7.39 (m, 5H), 7.27 (m, 2H), 6.70 (dd, *J* = 5.6, 1.6 Hz, 1H), 6.65 (d, *J* = 5.6 Hz, 1H), 5.33 (d, *J* = 1.6 Hz, 1H), 3.27 (d, *J* = 6.7 Hz, 1H), 3.25 (d, *J* = 6.7 Hz, 1H). ^{13}C{^{1}H} NMR (75 MHz, DMSO-d_6) δ 175.5, 173.4, 168.0, 137.74, 137.67, 132.6, 129.3, 129.0, 127.5, 90.7, 81.6, 50.1, 49.6. Calcd. for $C_{15}H_{12}N_2O_4$ [M + Na]: 307.0689. Found 307.0689.

Synthesis of *exo*-4a.

The mixture of 2-acetylfuran (110 mg, 1 mmol) and maleimide (146 mg, 1.5 mmol) was heated in a 4 mL glass vial with a screw cap in an aluminum block at 60 °C for 24 hours. The resulting viscous liquid was washed with Et_2O, yielding 33 mg (16% yield) of product *exo*-**4a** as a white precipitate.

***Exo*-4-acetyl-3a,4,7,7a-tetrahydro-1H-4,7-epoxyisoindole-1,3(2H)-dione (*exo*-4a).**

^1H NMR (300 MHz, DMSO-d_6) δ 11.33 (br. s, 1H), 6.68 (dd, *J* = 5.6, 1.7 Hz, 1H), 6.63 (d, *J* = 5.6 Hz, 1H), 5.22 (d, *J* = 1.7 Hz, 1H), 3.29 (d, *J* = 6.5 Hz, 1H), 3.03 (d, *J* = 6.5 Hz, 1H), 2.28 (s, 3H). $^{13}C\{^1H\}$ NMR (75 MHz, DMSO-d_6) δ 202.7, 177.4, 176.3, 138.3, 136.1, 94.6, 81.1, 51.9, 50.6, 27.6. m/z HRMS (ESI) Calcd. for $C_{10}H_9NO_4$ [M + Na]: 230.0424. Found 230.0429.

Synthesis of *exo*-4b.

The mixture of 2-acetylfuran (110 mg, 1 mmol) and *N*-ethyl maleimide (146 mg, 1.5 mmol) was heated in a 4 mL glass vial with a screw cap in an aluminum block at 60 °C for 24 hours. The resulting viscous liquid was washed with Et_2O, yielding 42 mg (18% yield) of product *exo*-**4b** as a white precipitate.

***Exo*-4-acetyl-2-ethyl-3a,4,7,7a-tetrahydro-1H-4,7-epoxyisoindole-1,3(2H)-dione (*exo*-4b).**

^1H NMR (300 MHz, DMSO-d_6) δ 6.69 (dd, *J* = 5.6, 1.7 Hz, 1H), 6.64 (d, *J* = 5.6 Hz, 1H), 5.24 (d, *J* = 1.7 Hz, 1H), 3.37 (q, *J* = 7.2 Hz, 2H, overlapped with residual water signal), 3.32 (d, *J* = 6.4 Hz, 1H, , overlapped with residual water signal), 3.09 (d, *J* = 6.4 Hz, 1H), 2.26 (s, 3H), 1.02 (t, *J* = 7.2 Hz, 3H). $^{13}C\{^1H\}$ NMR (75 MHz, DMSO-d_6) δ 202.8, 175.9, 174.7, 138.2, 136.1, 94.5, 81.1, 50.6, 49.2, 33.5, 27.5, 13.2. m/z HRMS (ESI) Calcd. for $C_{12}H_{13}NO_4$ [M + Na]: 258.0737. Found 258.0744.

2.4. Study of the retro-DA reaction

Pure *exo*-**2a/2b** (0.1 mmol) and 3 eq. of maleimide/*N*-ethyl maleimide were placed in a 2 mL glass vial with a screw cap. Then the vial was heated in an aluminum block at 60 °C or 80 °C for 1-3 days. In other experiments, pure *exo*-**2a/2b** (0.1 mmol) were dissolved in 0.6 mL of DMSO-d_6 and were heated in an aluminum bath at 60 °C or 80 °C for 1-3 days. The resulting reaction mixtures were analyzed using ^{1}H NMR (Section 5.2). The obtained results are presented in Table S7.

Table S7. Results of the retro-DA for the adducts *exo*-**2a** and *exo*-**2b**.

№	Adduct	Conditions	2-Furoic acid/*endo*/ *exo* ratio, %)	Aggregate state[1]
1	*exo*-**2a**	3 eq. of maleimide, 60 °C, 3 days	0/0/100	Solid → solid[2]
2	*exo*-**2a**	3 eq. of maleimide, 80 °C, 1 day	0/0/100	Solid → solid[2]
3	*exo*-**2a**	DMSO-d_6, 60 °C, 3 days	89/0/12	-
4	*exo*-**2a**	DMSO-d_6, 80 °C, 1 day	98/0/2	-
5	*exo*-**2b**	3 eq. of *N*-ethyl maleimide, 60 °C, 3 days	8/2/90	Solid → solid + melt
6	*exo*-**2b**	3 eq. of *N*-ethyl maleimide, 80 °C, 1 day	48/6/46	Solid → melt
7	*exo*-**2b**	DMSO-d_6, 60 °C, 3 days	84/trace/16	-
8	*exo*-**2b**	DMSO-d_6, 80 °C, 1 day	96/0/4	-

[1]Changes in the aggregate state in the beginning and in the end of the reaction at the reaction temperature.
[2]Initial substrates did not melt during the reaction and remained in a solid state.

3. DSC experiments.

The monitoring of the reactions of 2-furoic acid with maleimide or HEM (see Figures S7, S11) was carried out by taking samples directly from the reaction mixture, followed by NMR and DSC analysis. The numbers in brackets on the DSC diagrams indicate the degree of conversion. "Mld" means "maleimide".

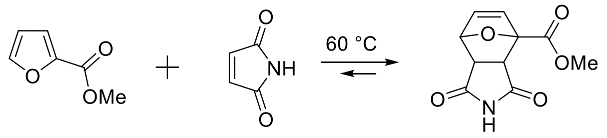

1a

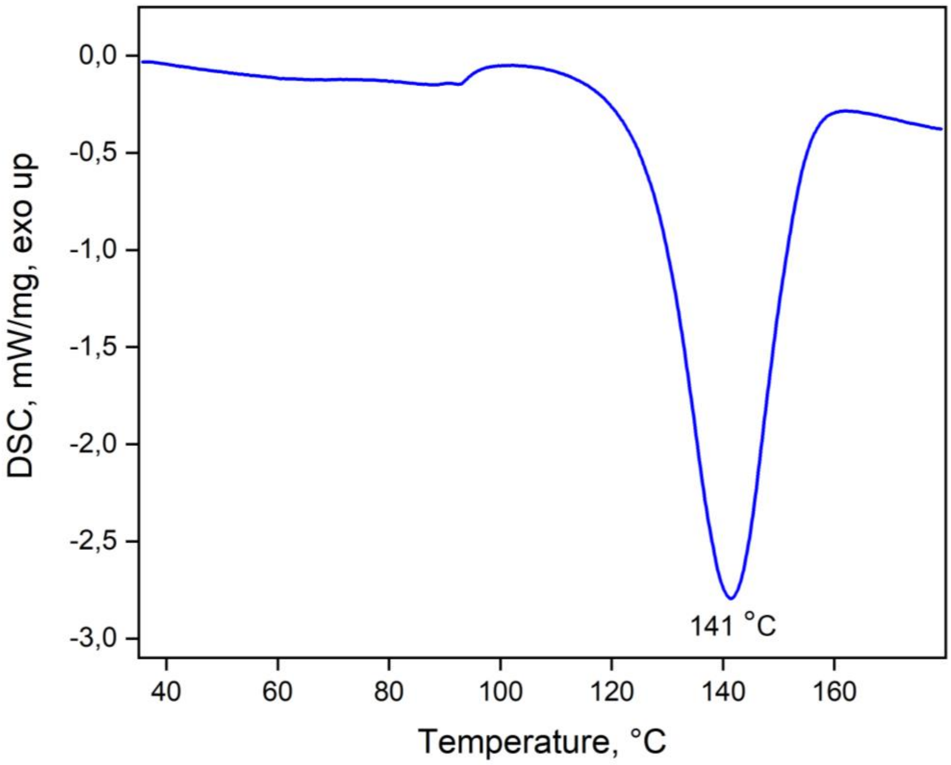

Figure S1. DSC thermogram of the reaction mixture obtained from the reaction of 2-methyl furoate with 1.1 eq. of maleimide (Table S2, entry 2).

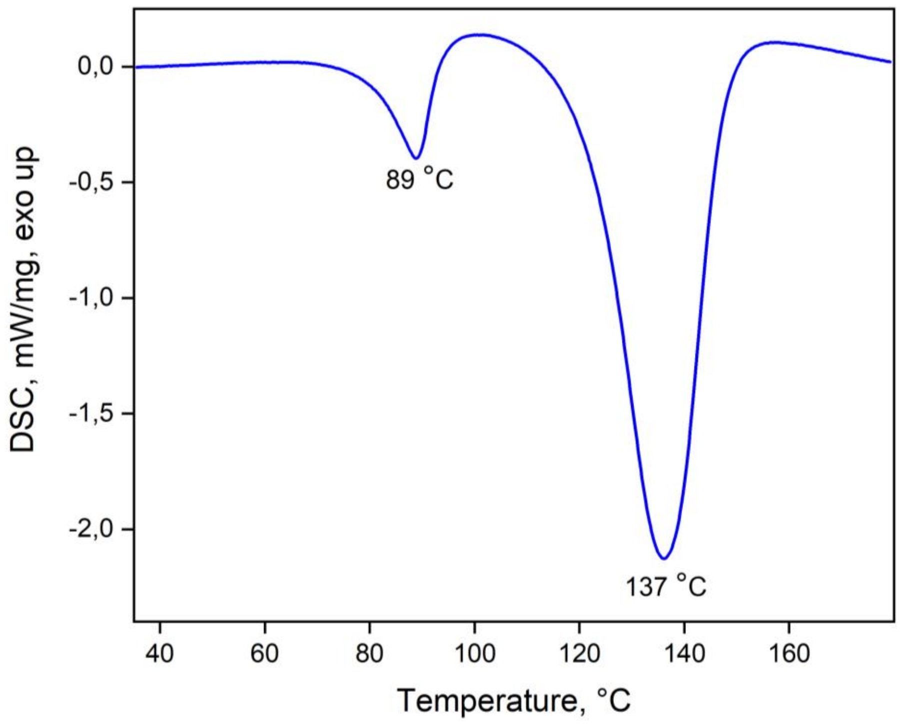

Figure S2. DSC thermogram of the reaction mixture obtained from the reaction of 2-methyl furoate with 1.5 eq. of maleimide (Table S2, entry 5).

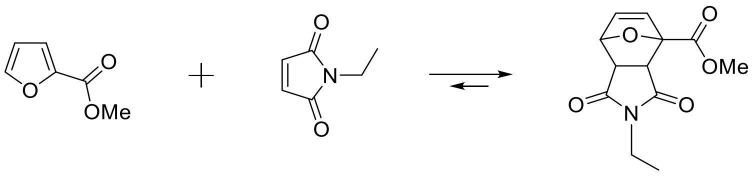

1b

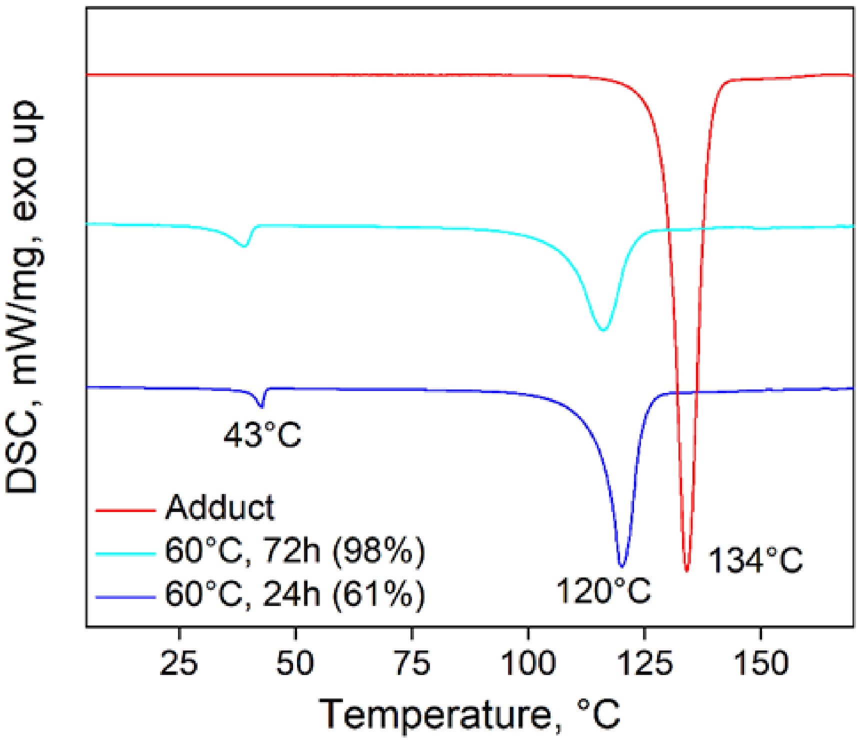

Figure S3. Comparison of DSC thermograms for the reaction mixtures obtained from the reactions of 2-methyl furoate with 1.5 eq. of *N*-ethyl maleimide (Table S2, entries 11, 12).

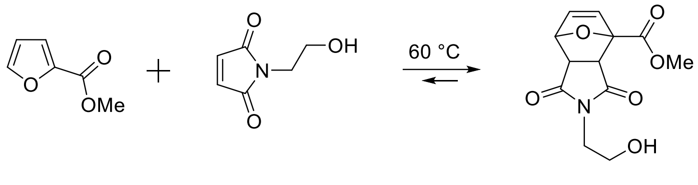

1c

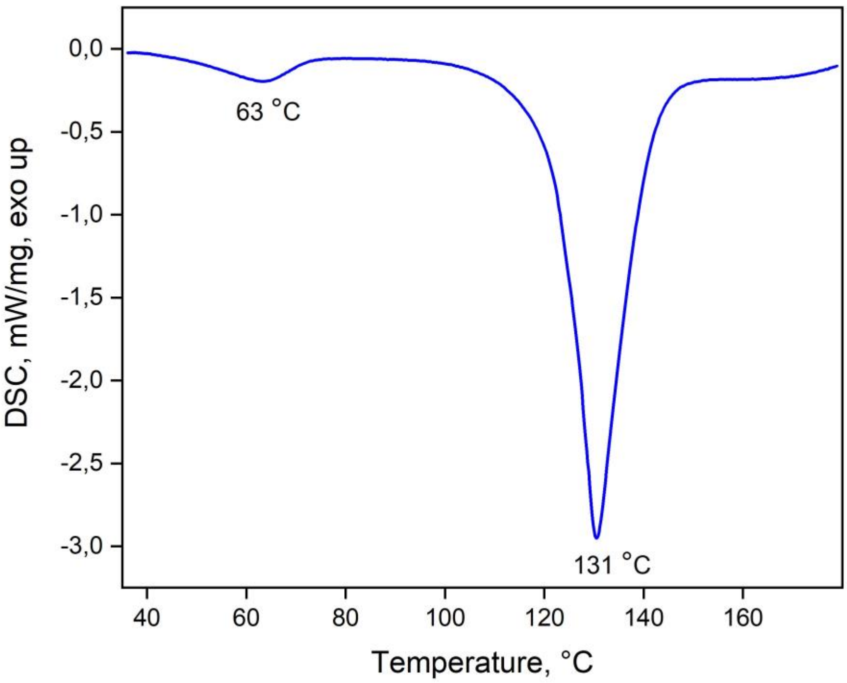

Figure S4. DSC thermogram of the reaction mixture obtained from the reaction of 2-methyl furoate with 1.25 eq. of HEM (Table S2, entry 14).

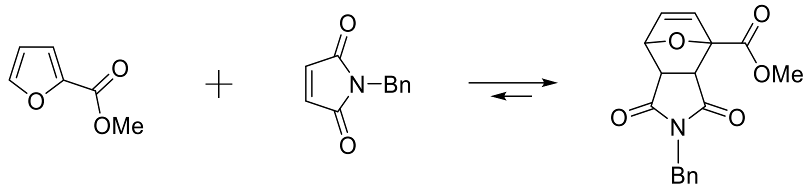

1d

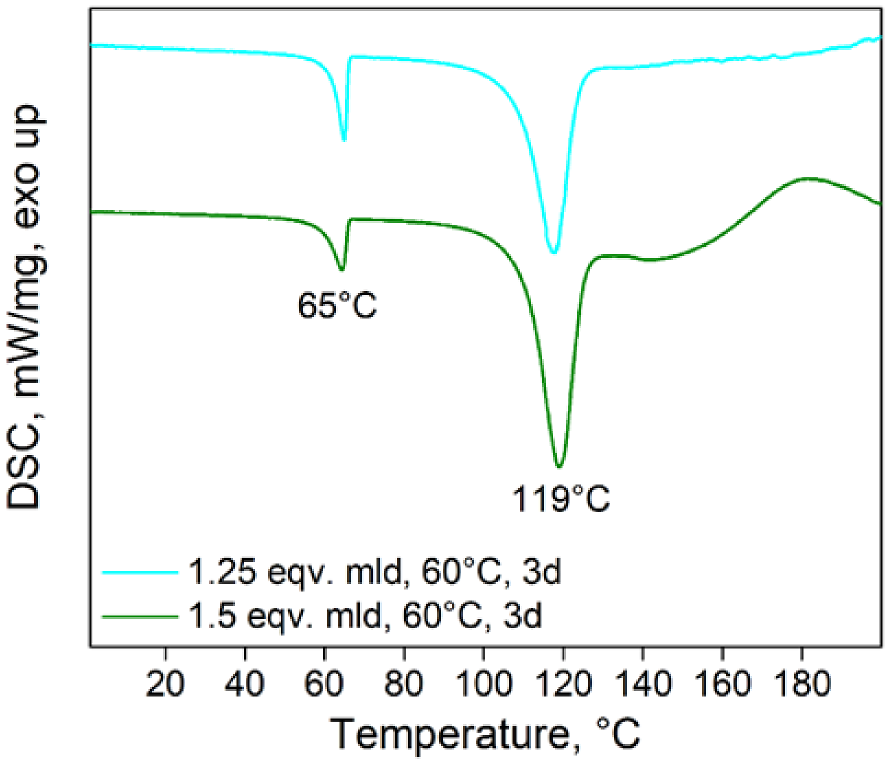

Figure S5. DSC thermograms of the reaction mixtures obtained from the reaction of 2-methyl furoate with various amounts of *N*-benzyl maleimide (Table S2, entries 18, 19).

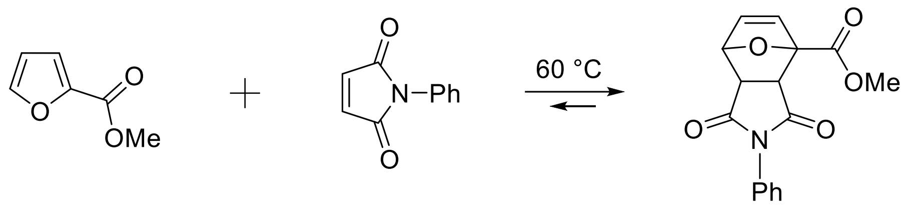

1e

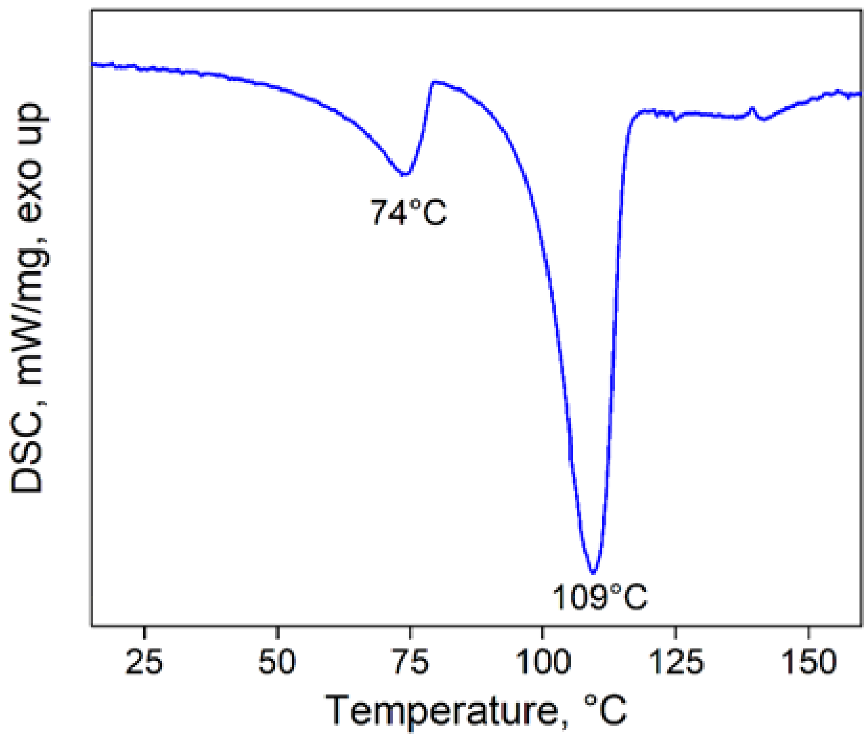

Figure S6. DSC thermogram of the reaction mixture obtained from the reaction of 2-methyl furoate with 1.25 eq. of *N*-phenyl maleimide (Table S2, entry 20).

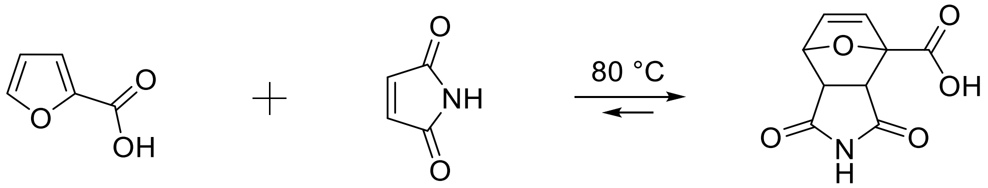

2a

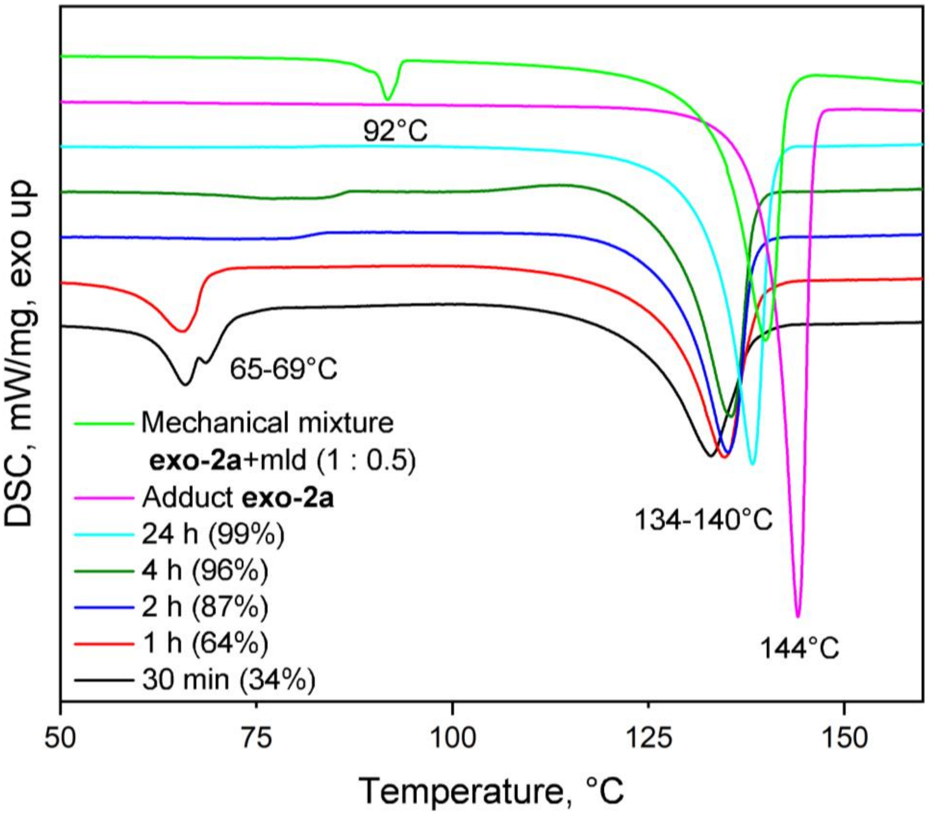

Figure S7. DSC monitoring of the reaction of 2-furoic acid with 1.5 eq. of maleimide (Table S3, entries 3-7).

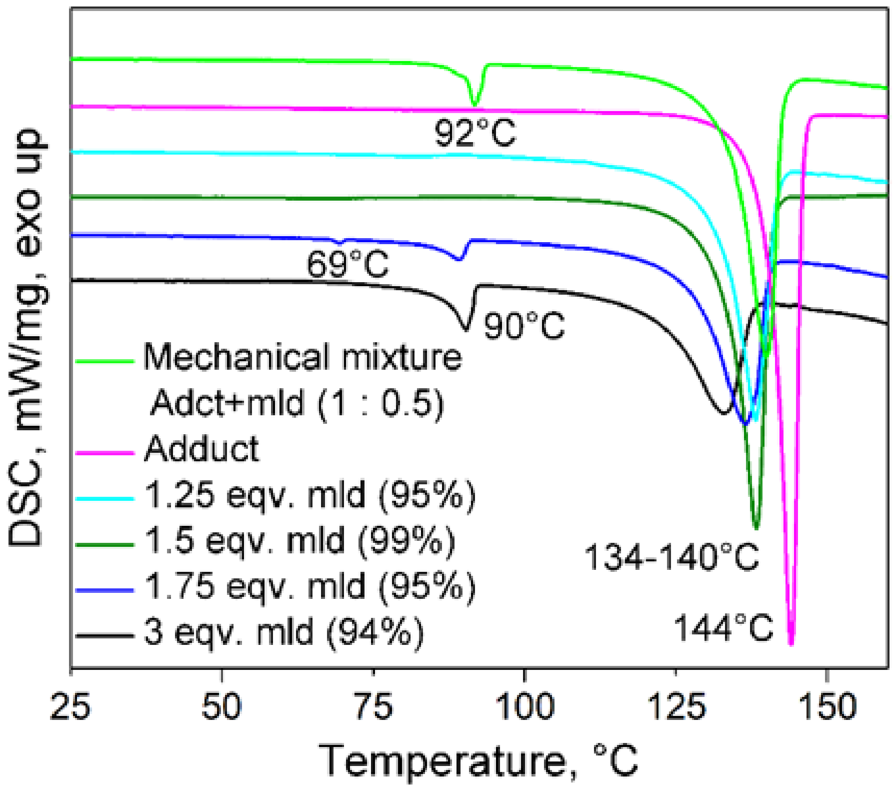

Figure S8. DSC thermograms of the reaction mixtures obtained from the reactions of 2-furoic acid with various amounts of maleimide (Table S3, entries 2, 7, 8, 10).

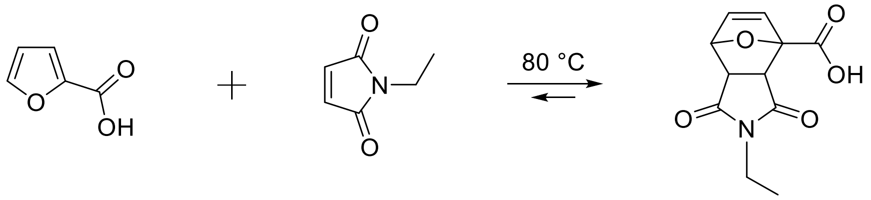

2b

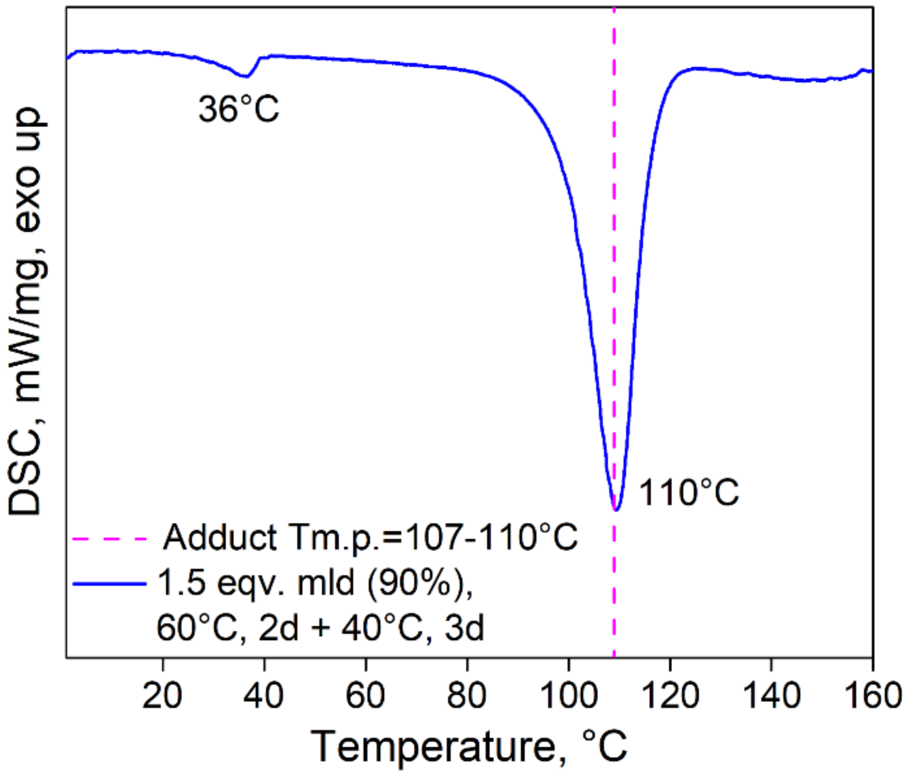

Figure S9. DSC thermogram of the reaction mixture obtained after the reactions of 2-furoic acid with 1.5 eq. of *N*-ethyl maleimide (Table S3, entry 15).

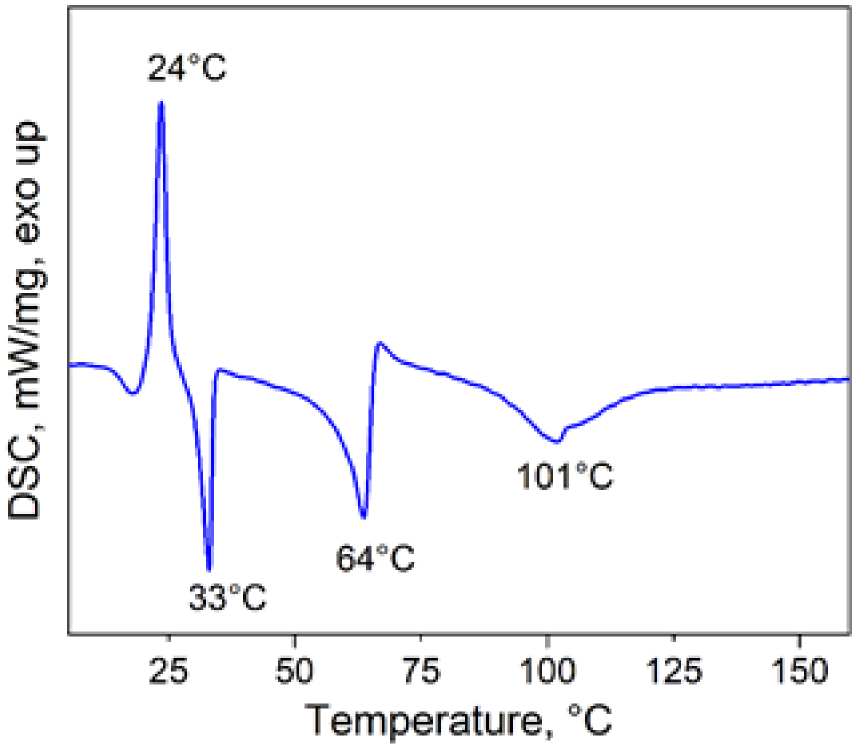

Figure S10. DSC thermogram of the reaction mixture obtained after the reactions of 2-furoic acid with 1.5 eq. of *N*-ethyl maleimide (Table S3, entry 14).

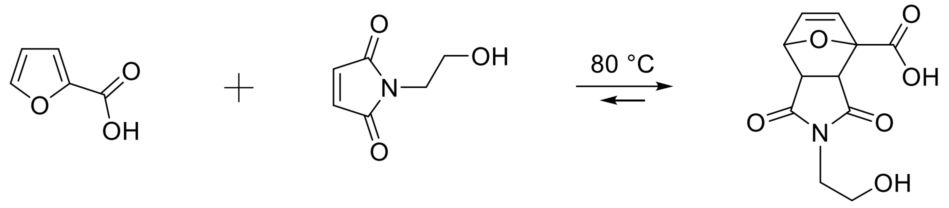

2c

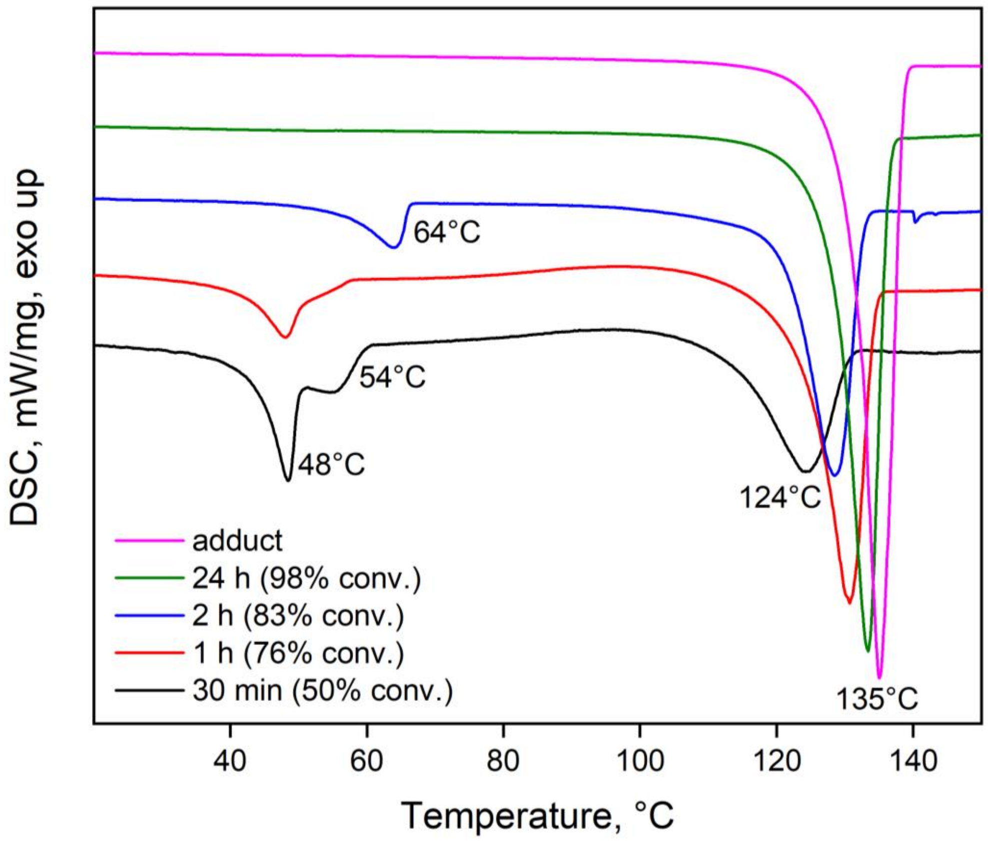

Figure S11. DSC monitoring of the reaction of 2-furoic acid with 1.5 eq. of HEM (Table S3, entries 18-21).

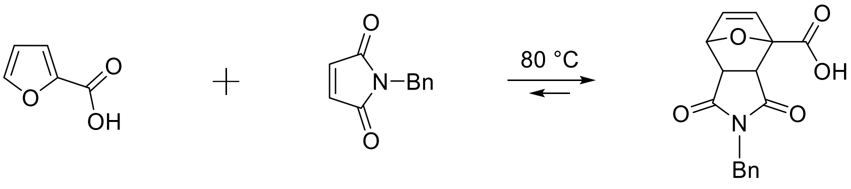

2d

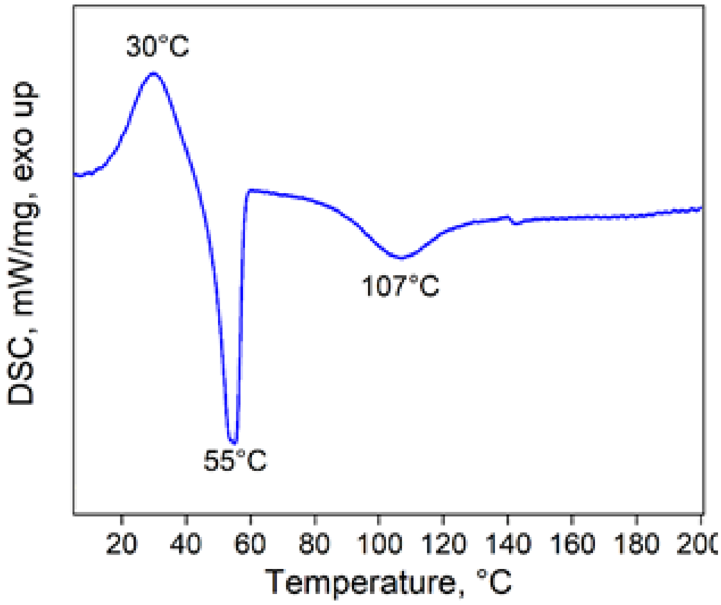

Figure S12. DSC thermogram of the reaction mixture obtained after the reactions of 2-furoic acid with 1.5 eq. of *N*-benzyl maleimide (Table S3, entry 24).

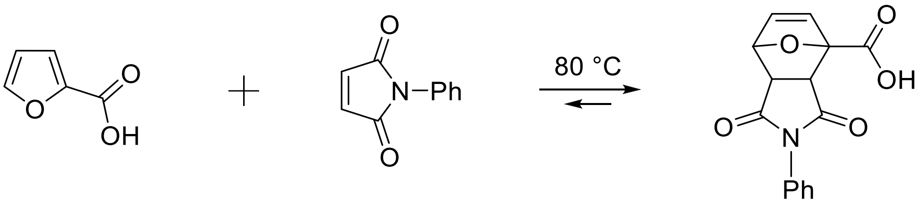

2e

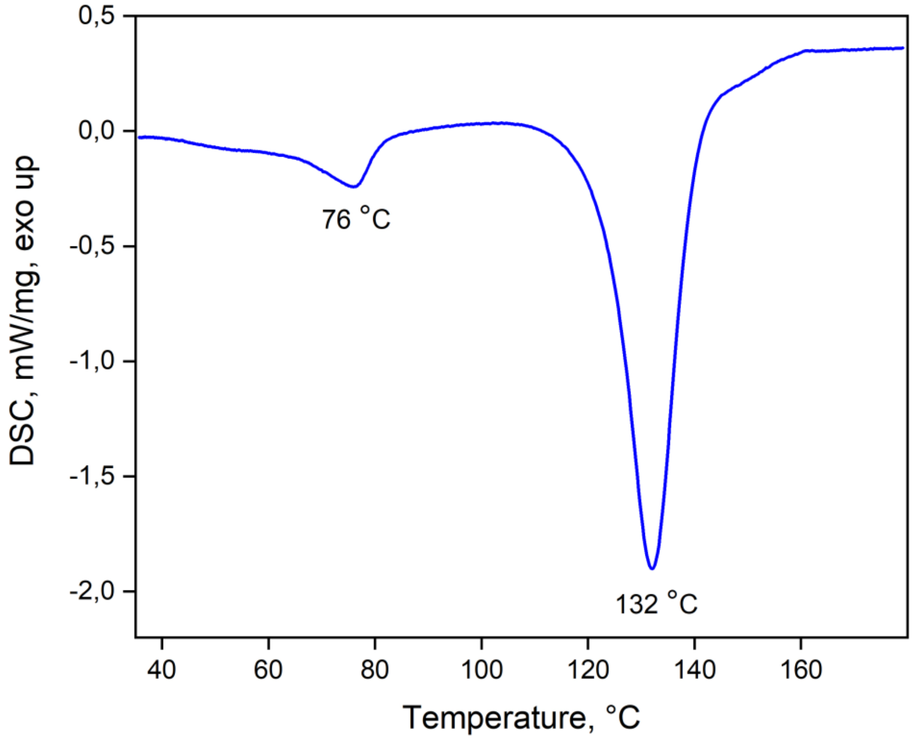

Figure S13. DSC thermogram of the reaction mixture obtained from the reactions of 2-furoic acid with 1.25 eq. of *N*-phenyl maleimide (Table S3, entry 25).

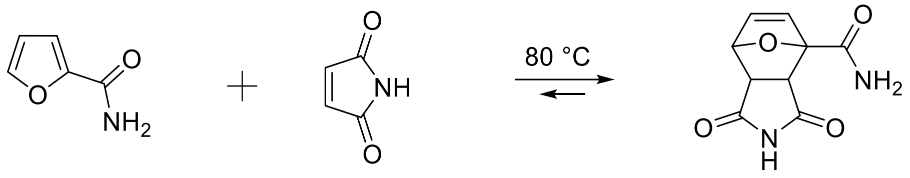

3a

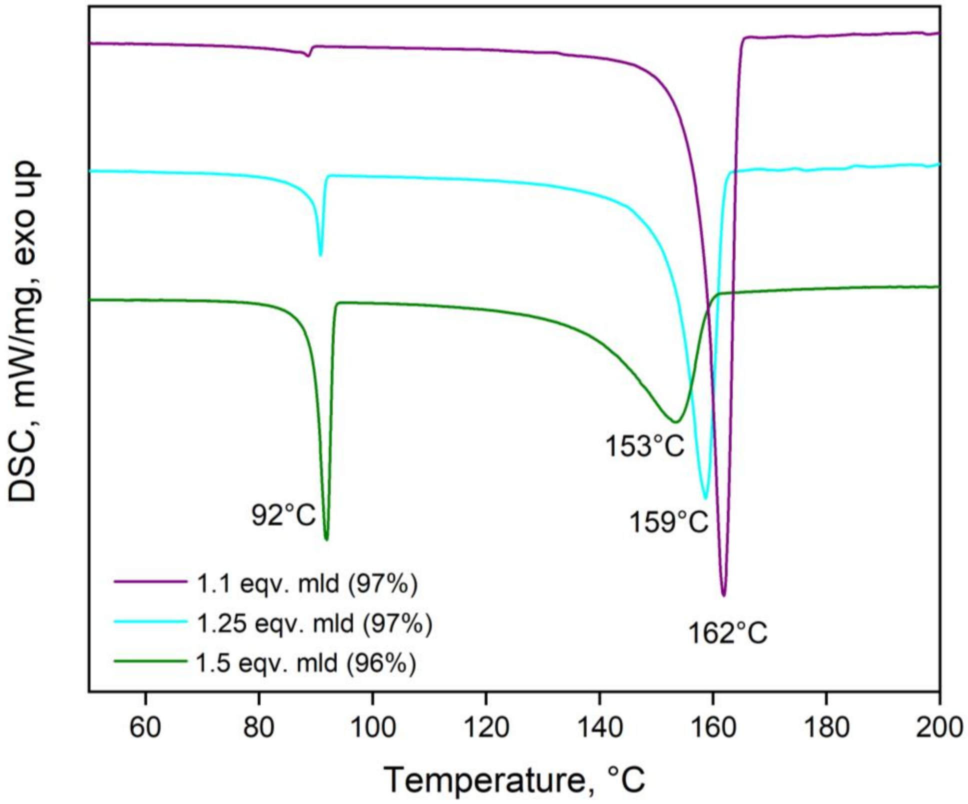

Figure S14. DSC thermograms of the reaction mixtures obtained from the reactions of 2-furamide with various amounts of maleimide (Table S4, entries 1, 2, 4).

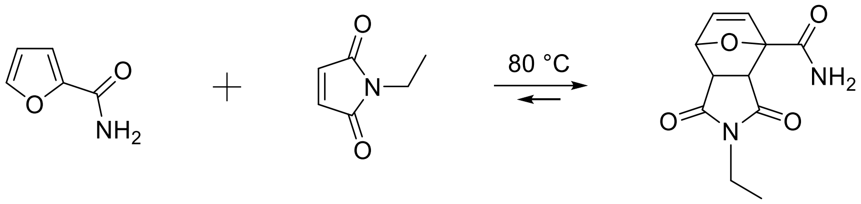

3b

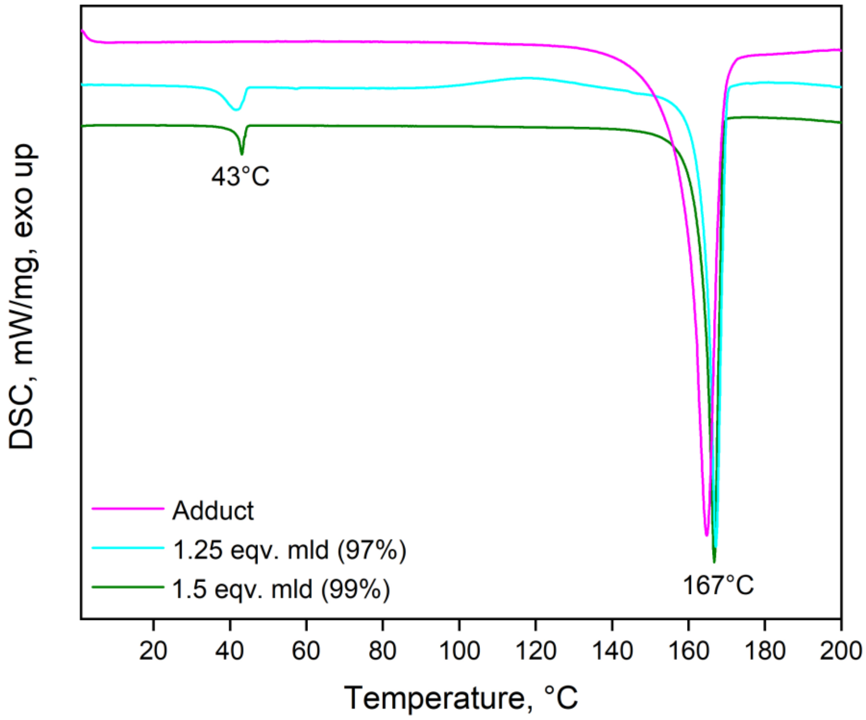

Figure S15. DSC thermograms of the reaction mixtures obtained from the reactions of 2-furoic acid with various amounts of *N*-ethyl maleimide (Table S4, entries 6, 8).

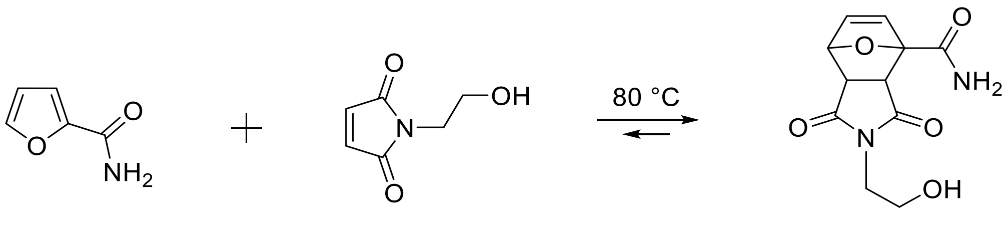

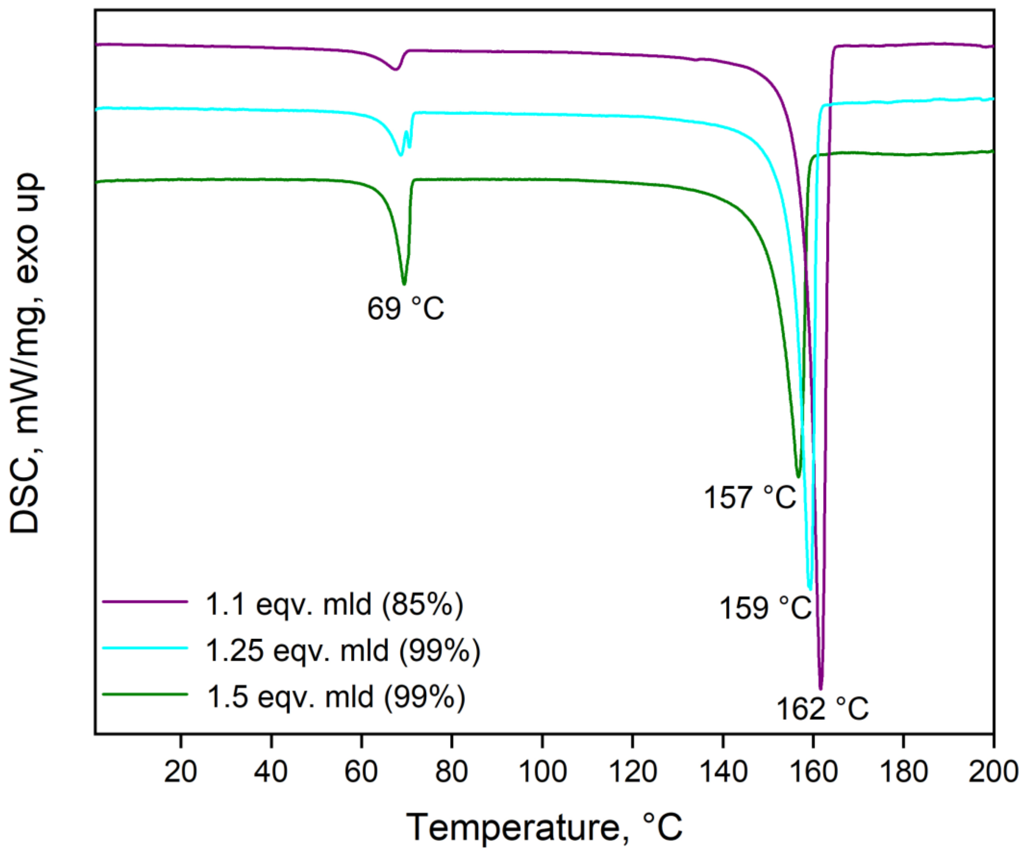

Figure S16. DSC thermograms of the reaction mixtures obtained from the reactions of 2-furoic acid with various amounts of HEM (Table S4, entries 11-13).

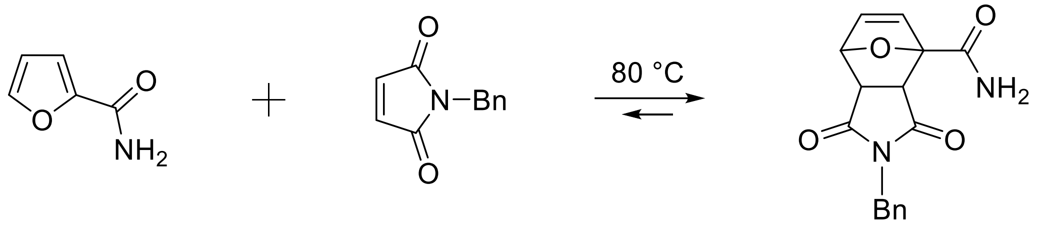

3d

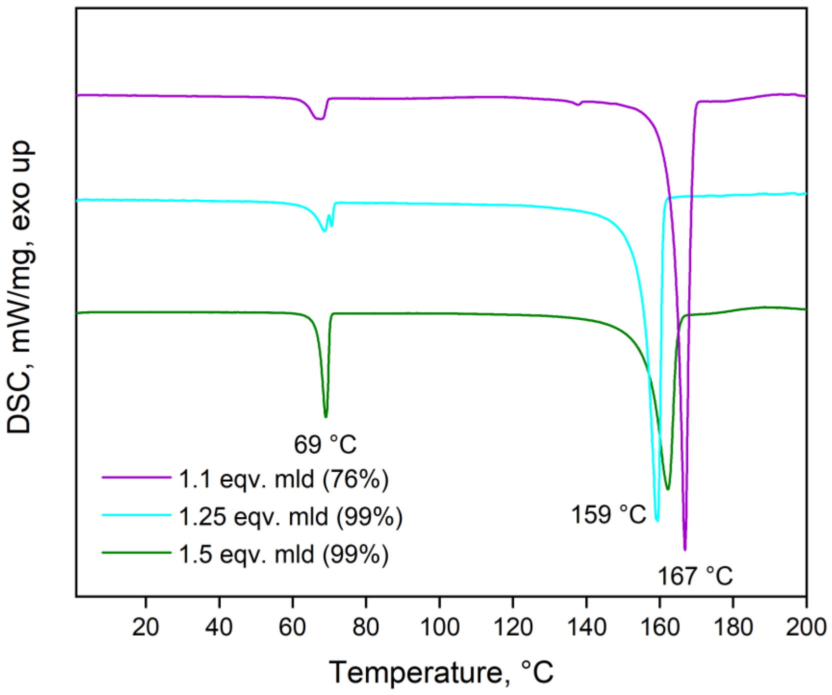

Figure S17. DSC thermograms of the reaction mixtures obtained from the reactions of 2-furoic acid with various amounts of *N*-benzyl maleimide (Table S4, entries 15-17).

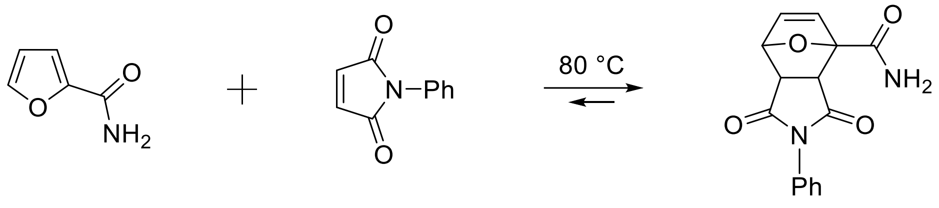

3e

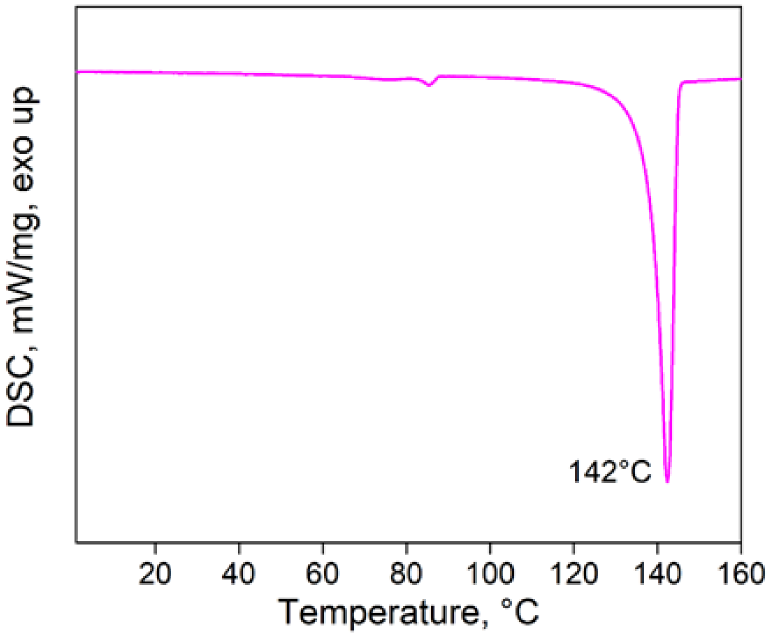

Figure S18. DSC thermograms of the reaction mixture obtained after the reaction of 2-furamide with *N*-phenyl maleimide (Table S4, entry 19).

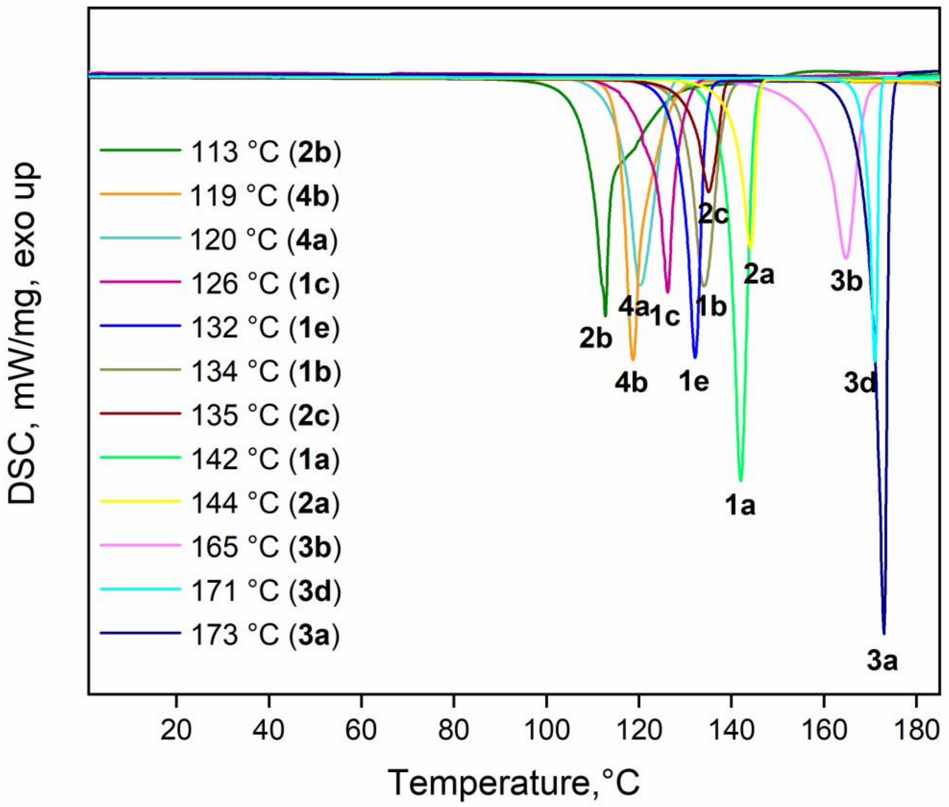

Figure S19. DSC thermograms for the melting of the selected pure fmDA adducts.

4. X-ray diffraction data

X-ray data for compound *exo*-1b.

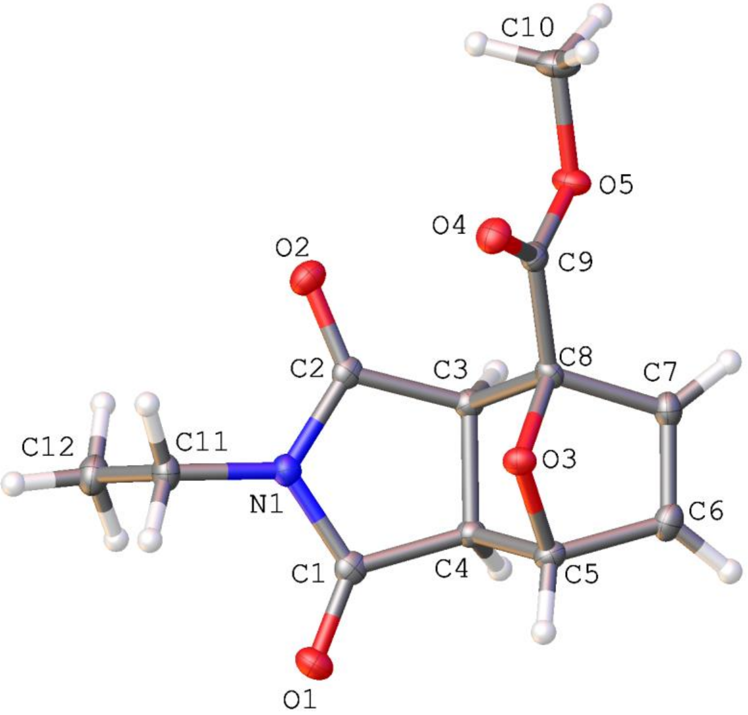

Figure S20. General view of the *exo*-**1b** molecule according to X-Ray analysis. Atoms are represented by thermal ellipsoids (p = 50%).

Table S8. Crystal data and structure refinement parameters for *exo*-**1b**.

Compound	*exo*-**1b**
Empirical formula	$C_{12}H_{13}NO_5$
Formula weight	251.23
T, K	100
Crystal system	Orthorhombic
Space group	$P2_12_12_1$
Z	4
a, Å	6.3726(4)
b, Å	11.0301(7)
c, Å	16.0981(10)
α, °	90
β, °	90
γ, °	90
V, Å^3	1131.54(12)
D_{calc} (g cm^{-1})	1.475
μ, cm^{-1}	1.16
F(000)	528
$2\theta_{max}$, °	50
Reflections measured	12128
Independent reflections	3001
Observed reflections [$I > 2\sigma(I)$]	2591
Parameters	166
R1	0.0392
wR2	0.0861
GOF	1.022
$\Delta\rho_{max}$/ $\Delta\rho_{min}$ (e Å^{-3})	0.199/-0.179

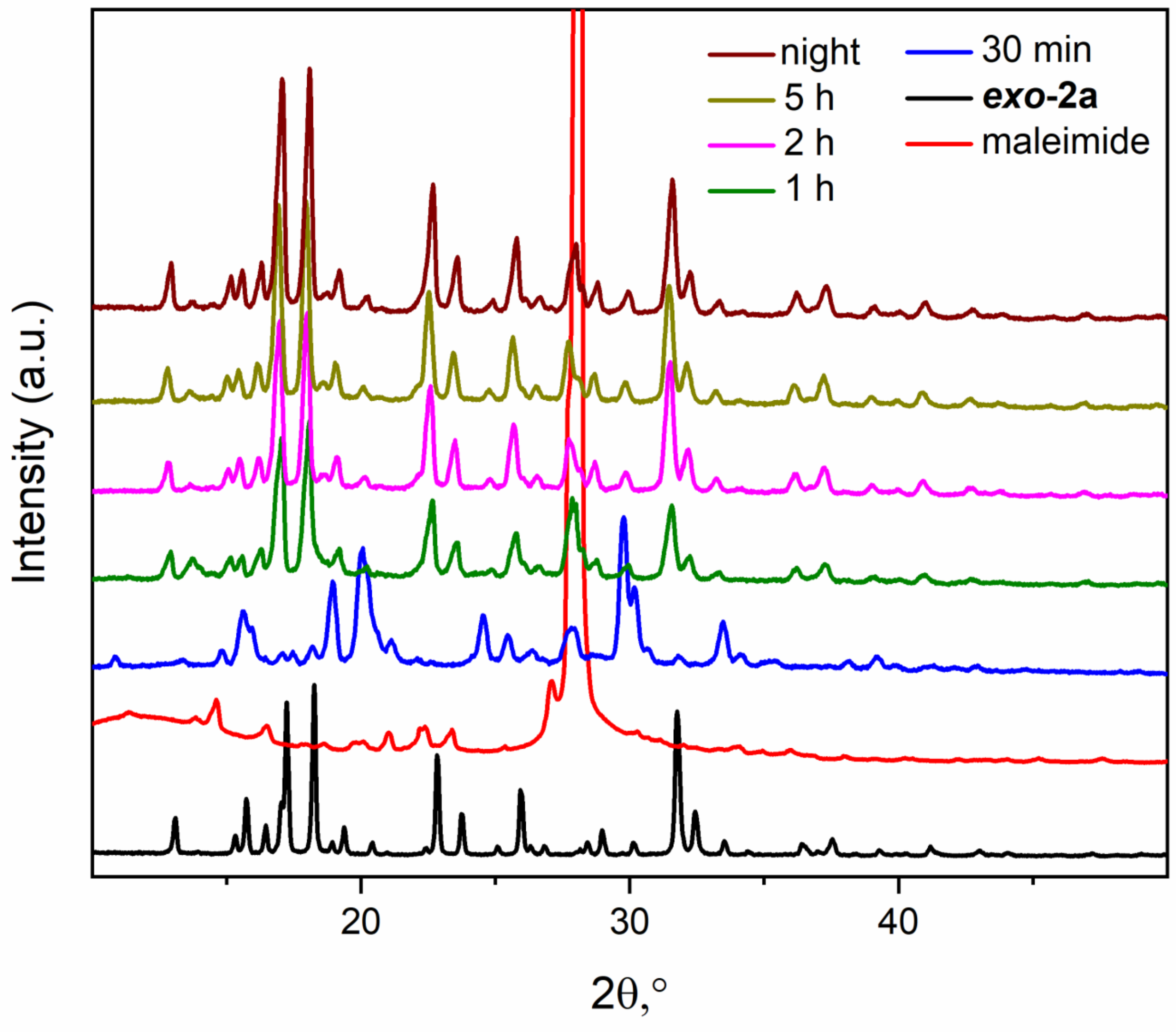

Figure S21. PXRD monitoring of the reaction mixture (conditions from Table S2, entries 3-7) in comparison with PXRD spectra of pure *exo*-**2a** and maleimide.

5. NMR spectra

5.1. Selected NMR spectra of the reaction mixtures

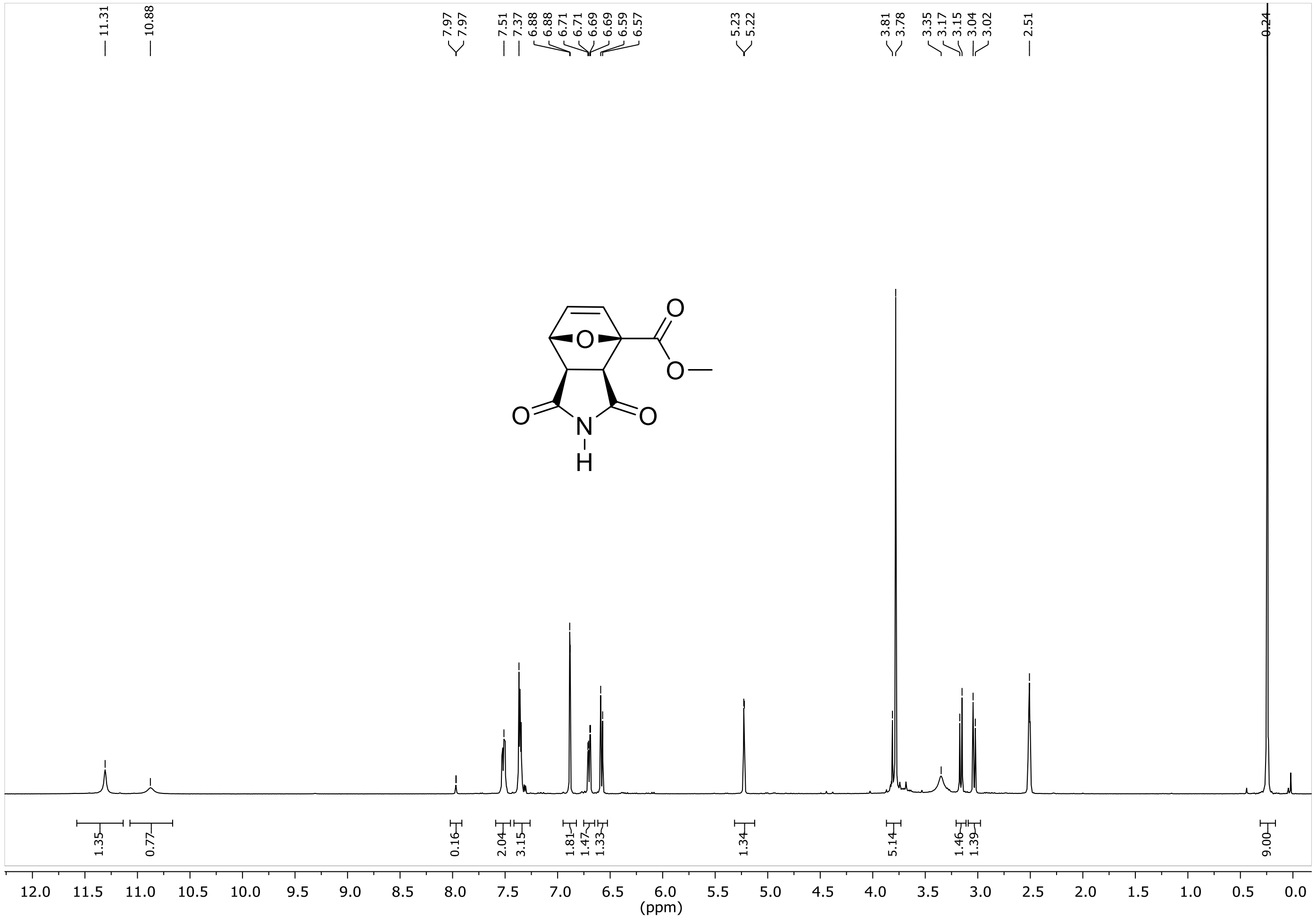

Figure S22. ^{1}H NMR spectrum (300 MHz, DMSO-d_6) of the reaction mixture for the conditions from Table S2, entry 3.

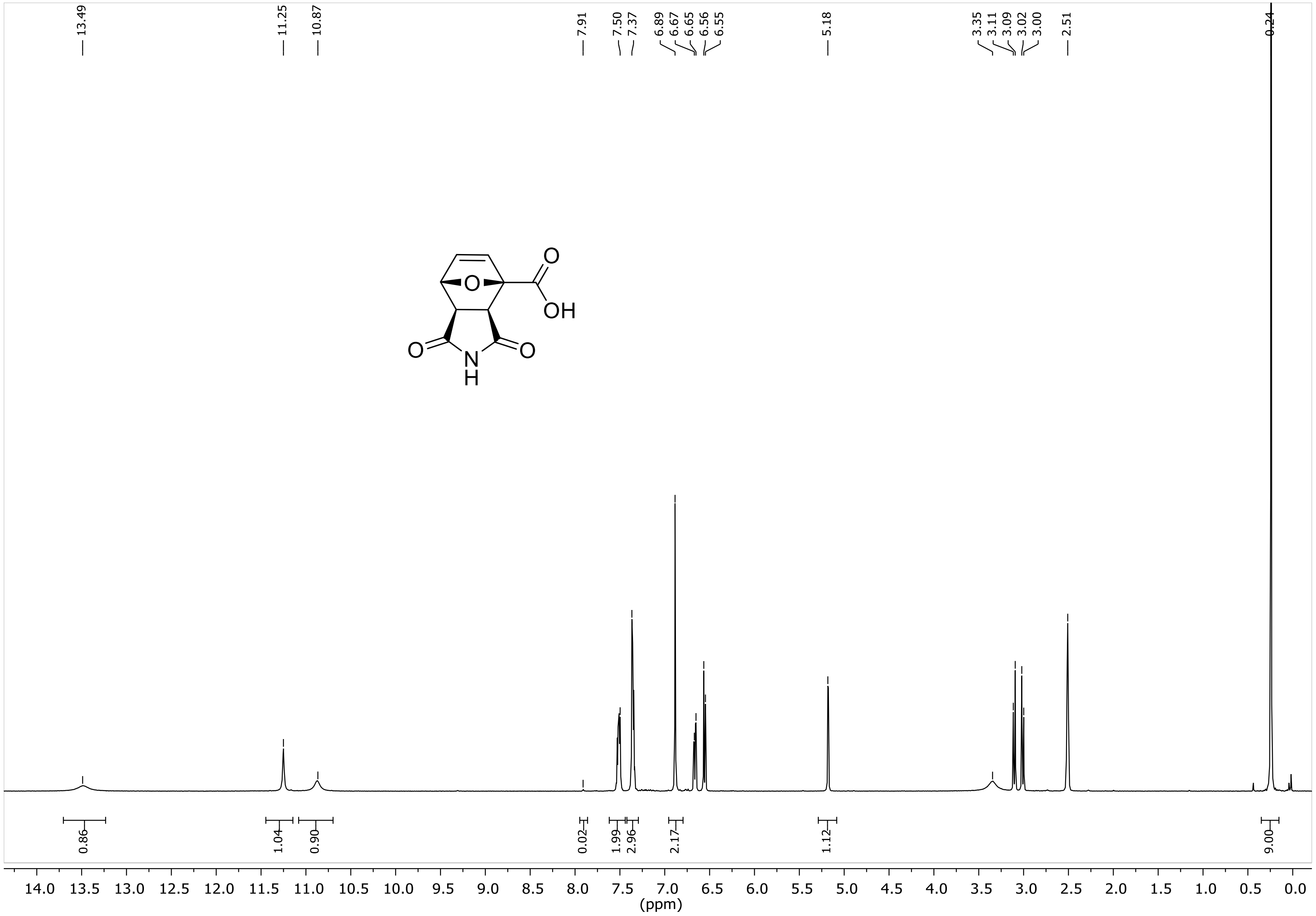

Figure S23. ^{1}H NMR spectrum (300 MHz, DMSO-d_6) of the reaction mixture for the conditions from Table S3, entry 2.

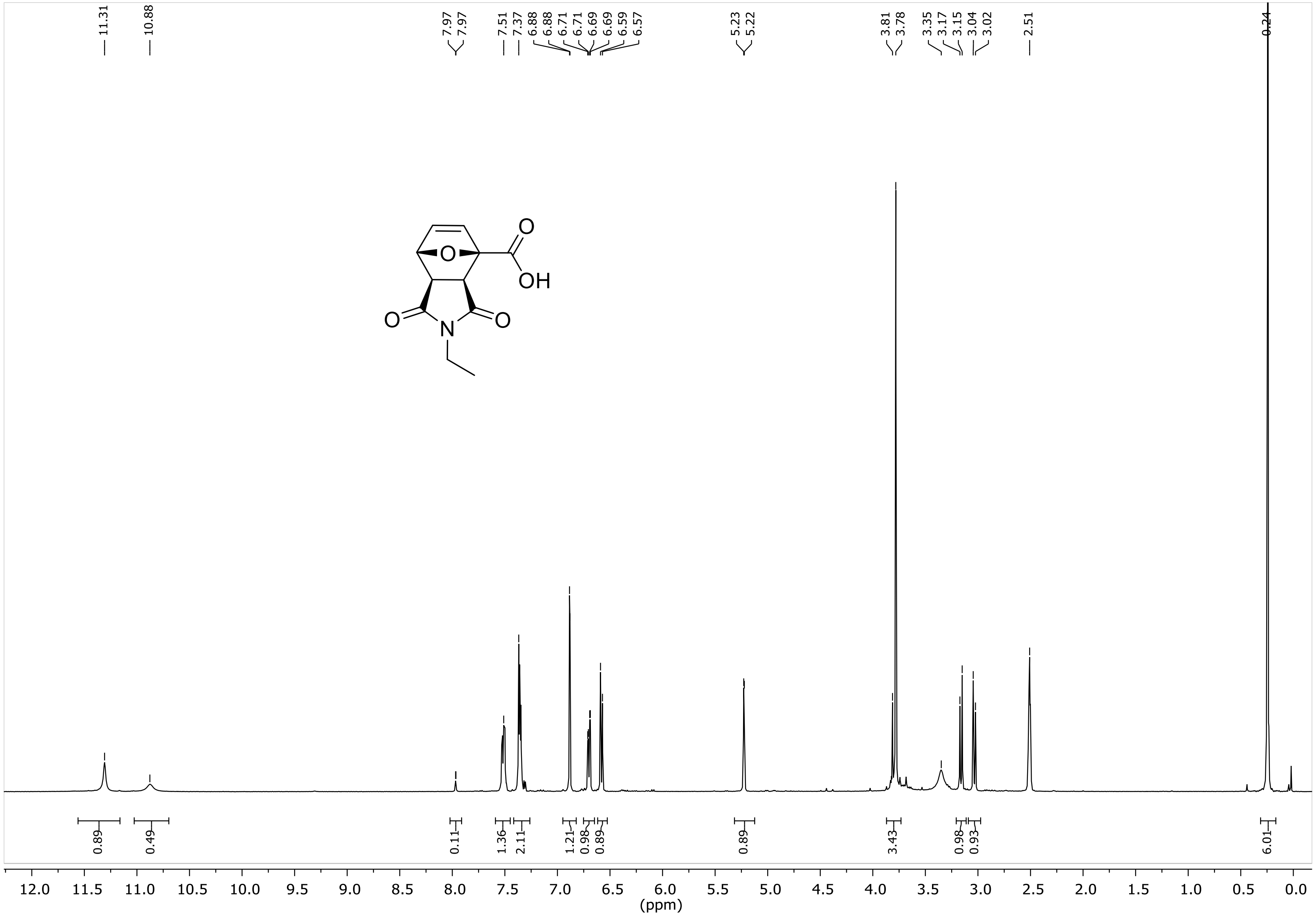

Figure S24. ^{1}H NMR spectrum (300 MHz, DMSO-d_6) of the reaction mixture for the conditions from Table S3, entry 16.

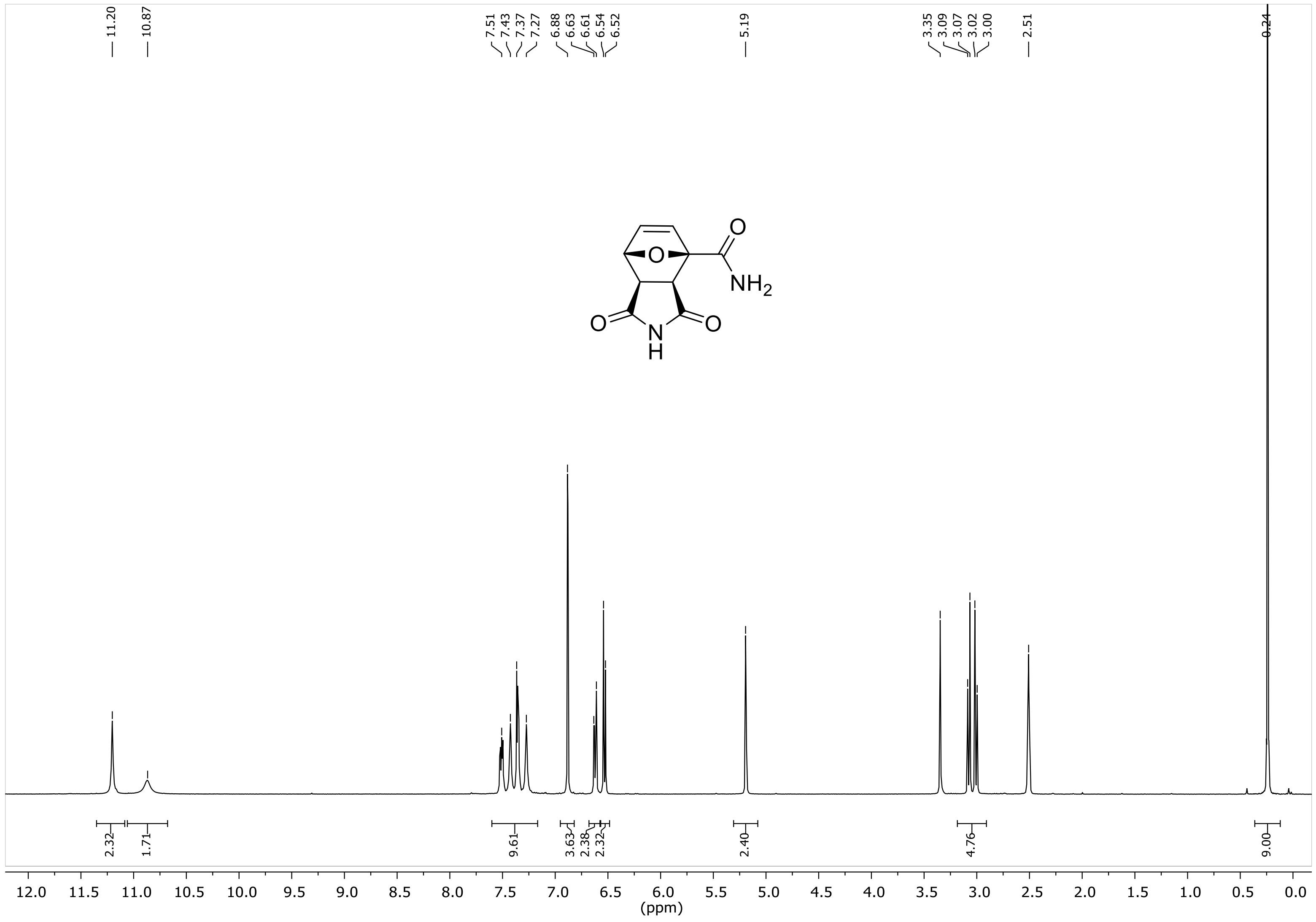

Figure S25. ^{1}H NMR spectrum (300 MHz, DMSO-d_6) of the reaction mixture for the conditions from Table S4, entry 2.

5.2. NMR spectra of the retro-DA reactions

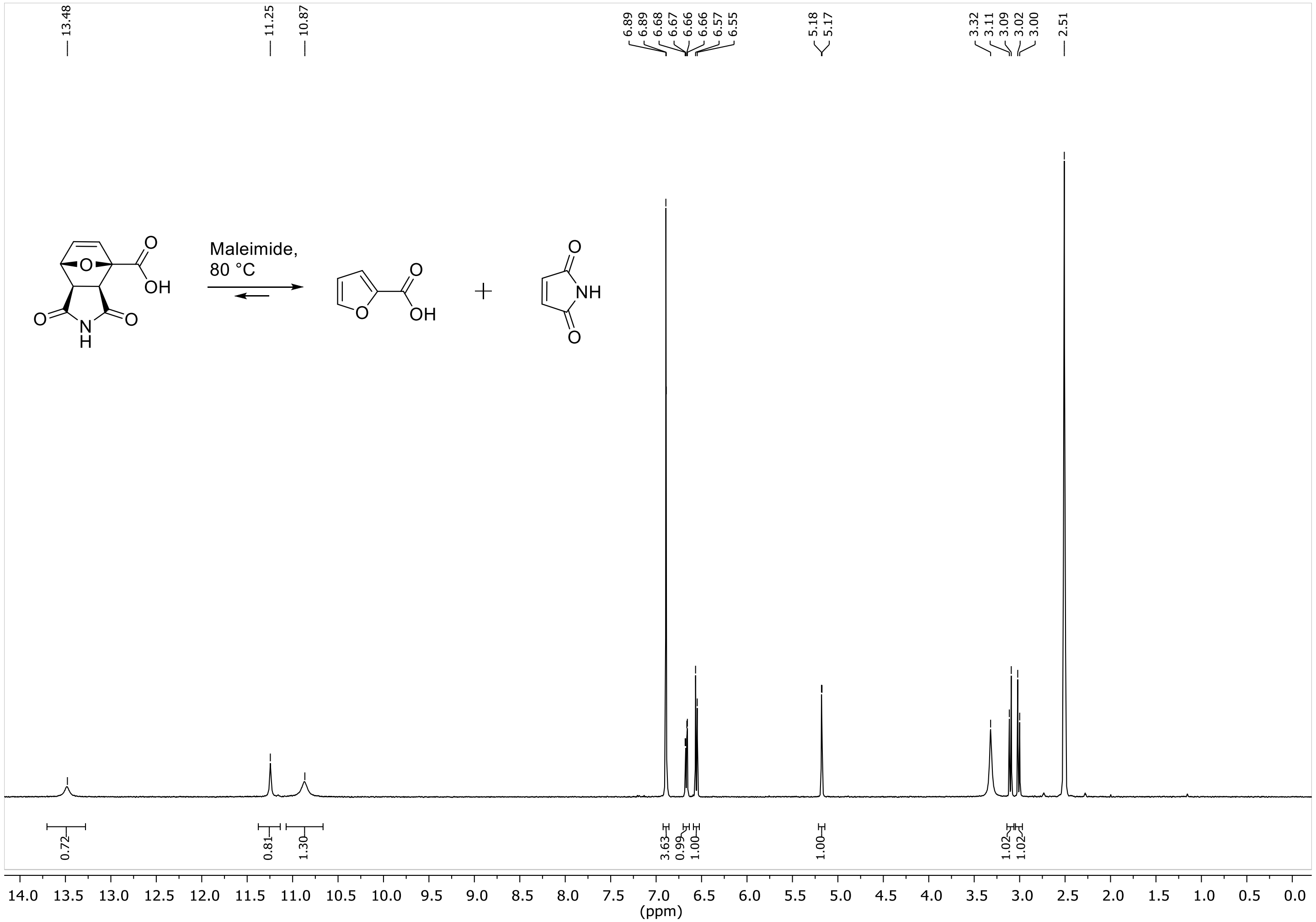

Figure S26. ^{1}H NMR spectrum (300 MHz, DMSO-d_6) of the retro DA reaction mixture for the conditions from Table S7, entry 2.

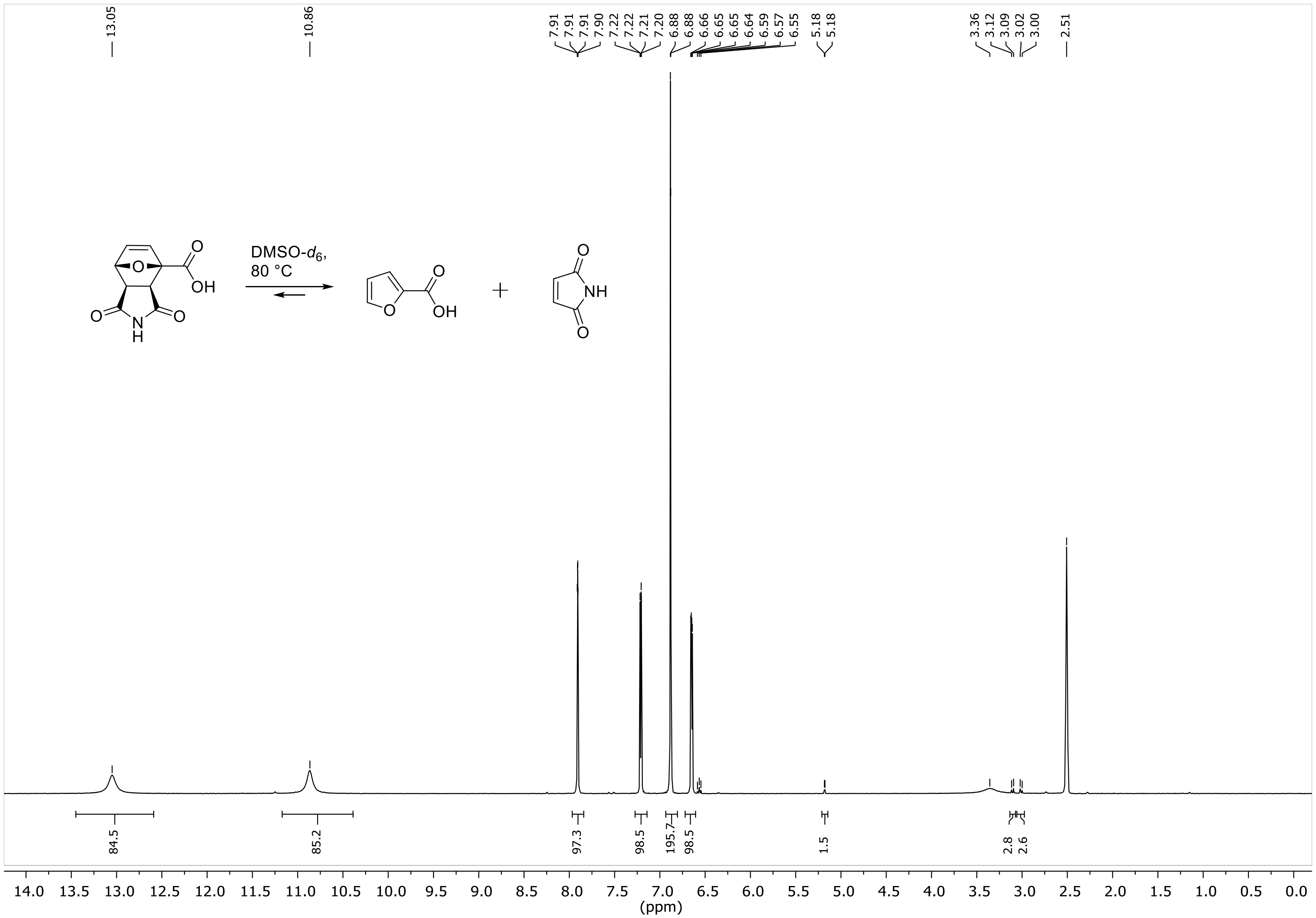

Figure S27. ^{1}H NMR spectrum (300 MHz, DMSO-d_6) of the retro DA reaction mixture for the conditions from Table S7, entry 4.

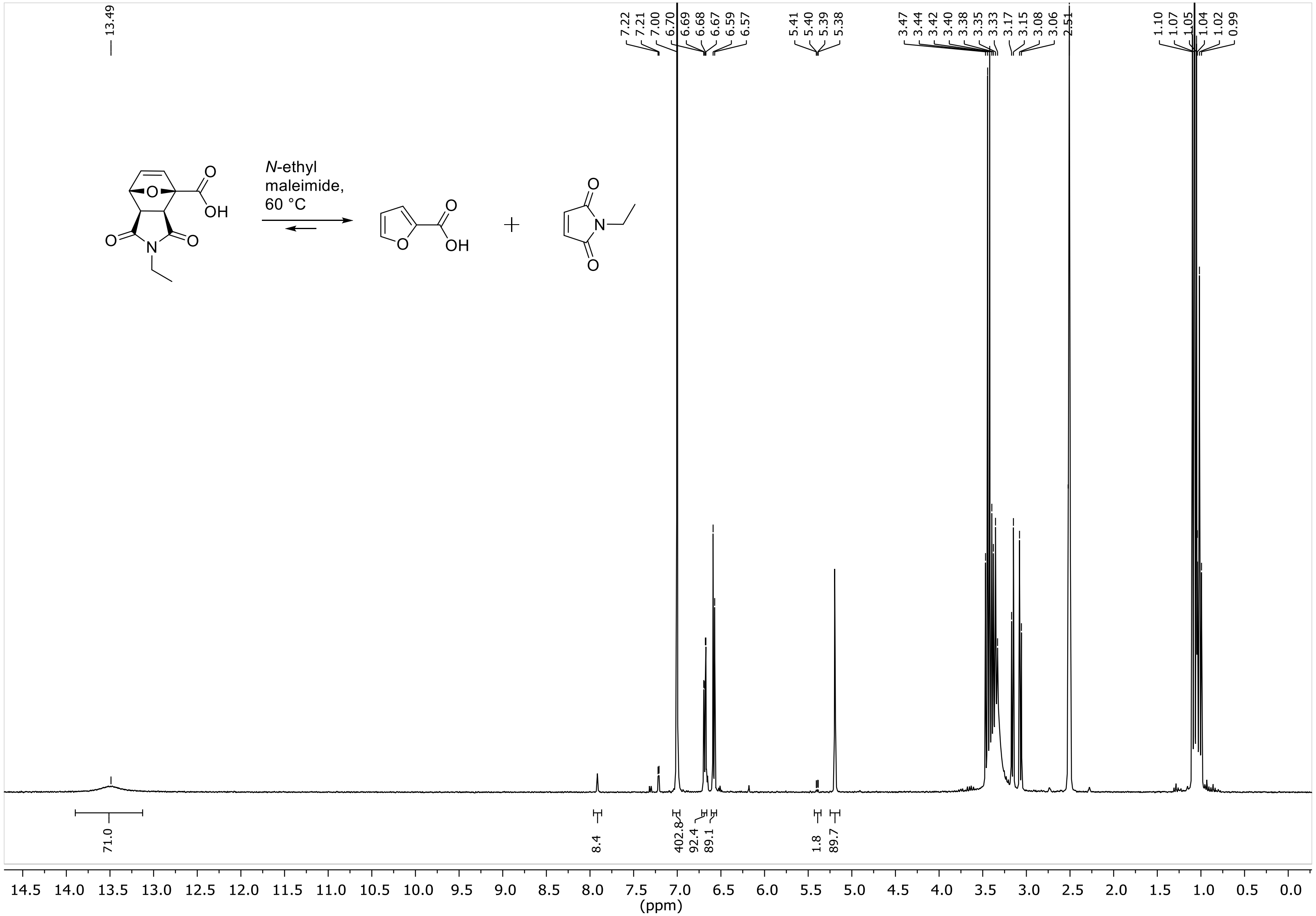

Figure S28. ^{1}H NMR spectrum (300 MHz, DMSO-d_6) of the retro DA reaction mixture for the conditions from Table S7, entry 5.

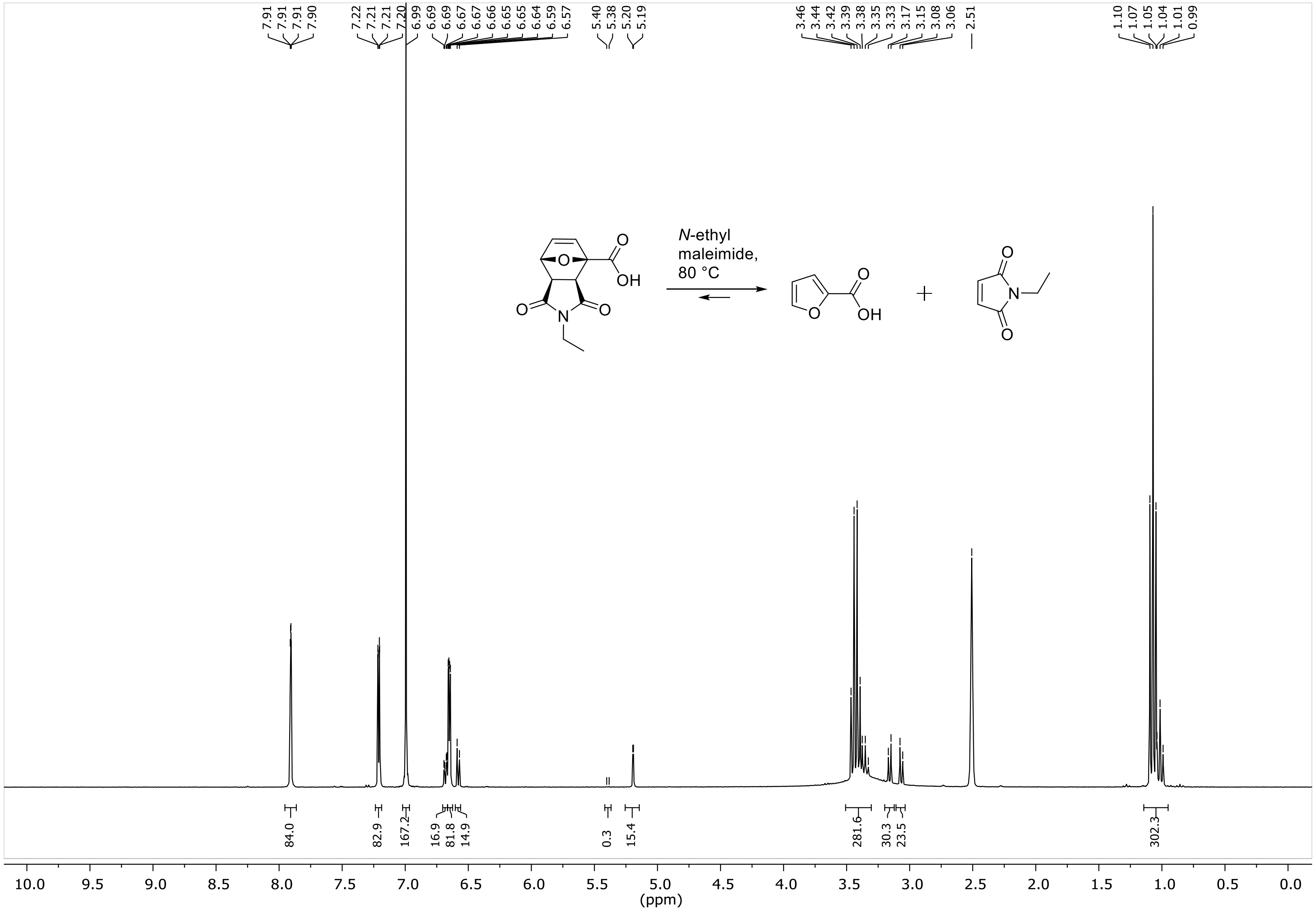

Figure S29. ^{1}H NMR spectrum (300 MHz, DMSO-d_6) of the retro DA reaction mixture for the conditions from Table S7, entry 6.

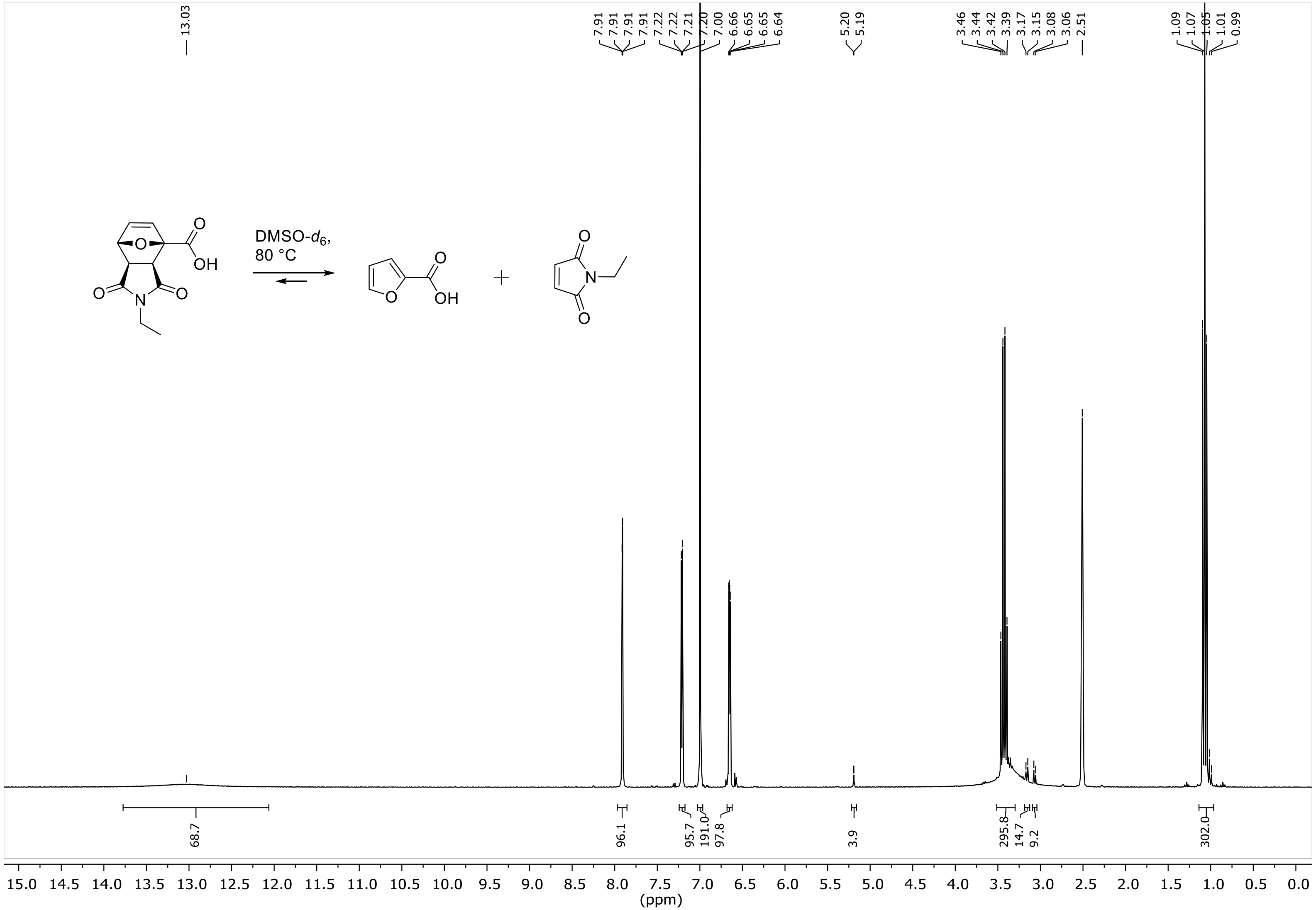

Figure S30. ^{1}H NMR spectrum (300 MHz, DMSO-d_6) of the retro DA reaction mixture for the conditions from Table S7, entry 8.

5.3. NMR spectra of the isolated *exo*-adducts

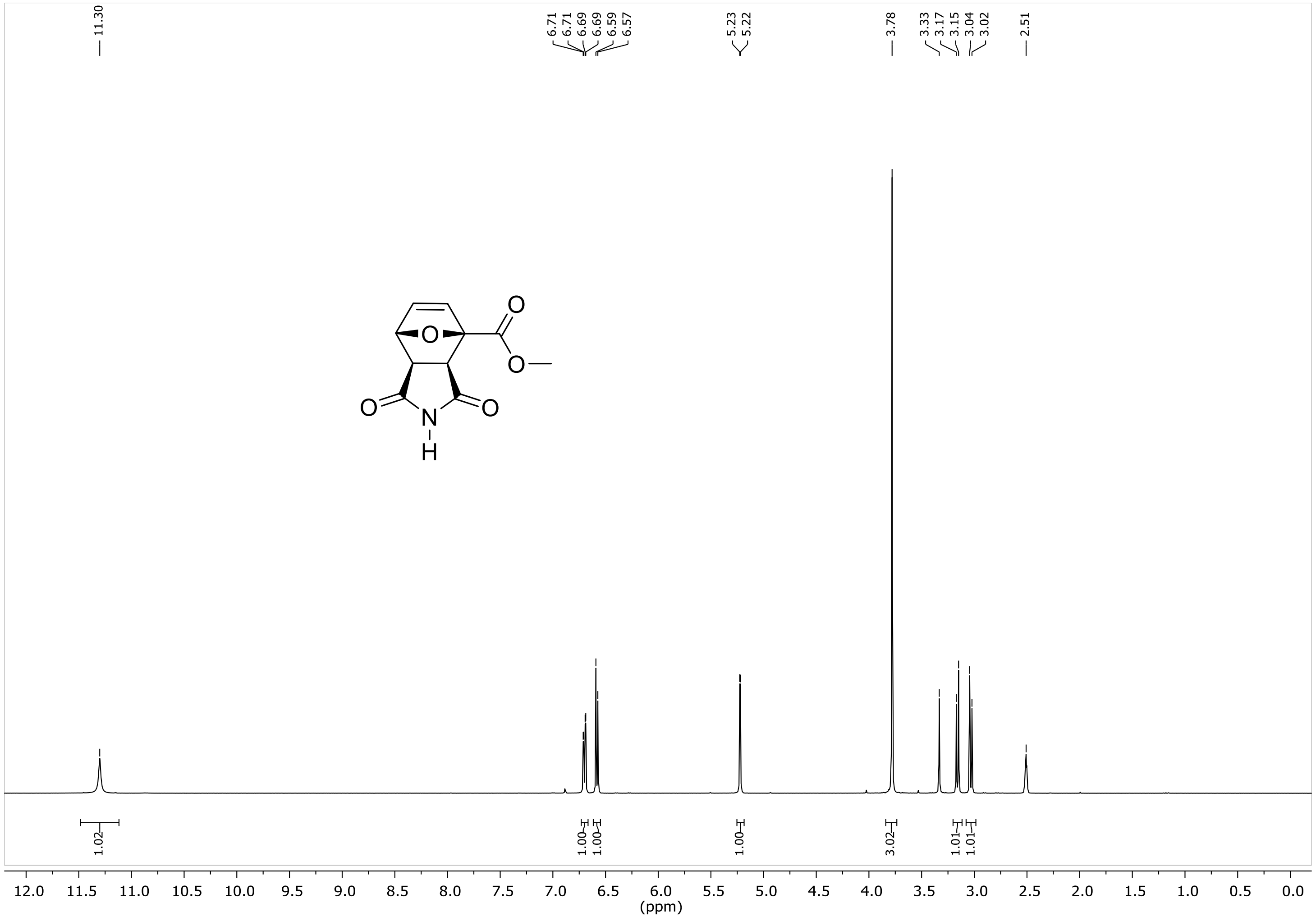

Figure S31. ^{1}H NMR spectrum (300 MHz, DMSO-d_6) of *exo*-**1a**.

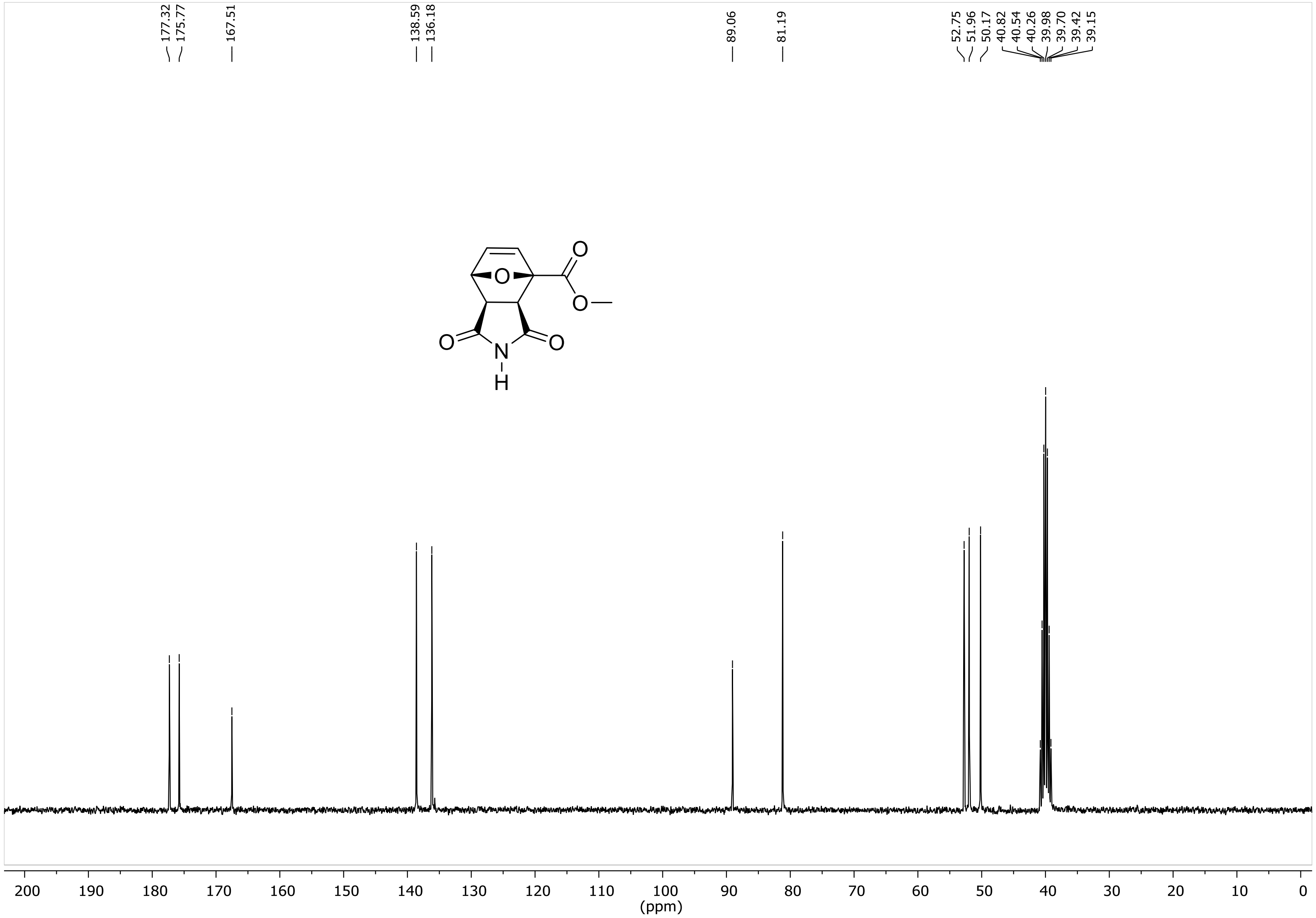

Figure S32. $^{13}C\{^{1}H\}$ NMR spectrum (75 MHz, DMSO-d_6) of *exo*-**1a**.

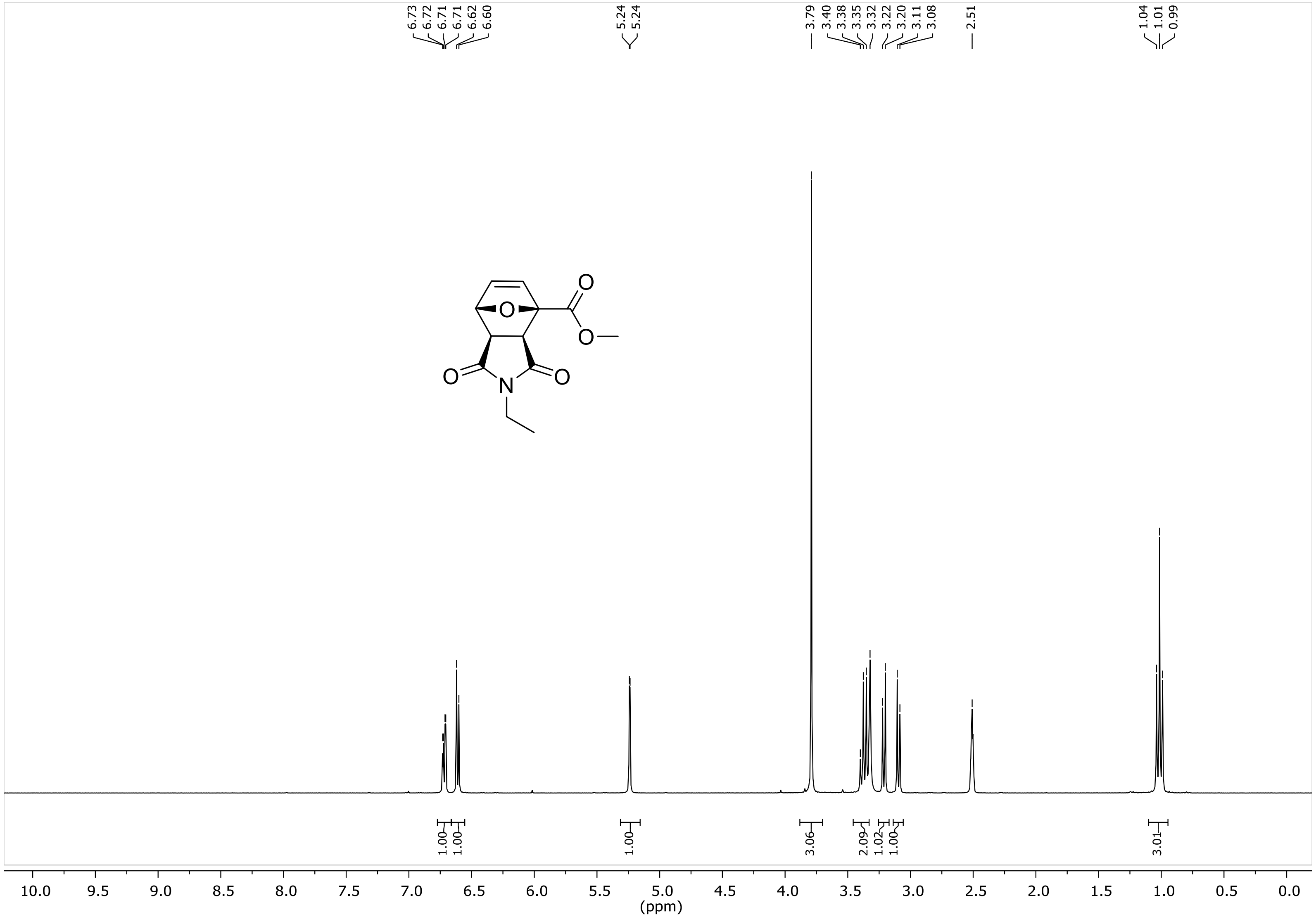

Figure S33. ^{1}H NMR spectrum (300 MHz, DMSO-d_6) of *exo*-**1b**.

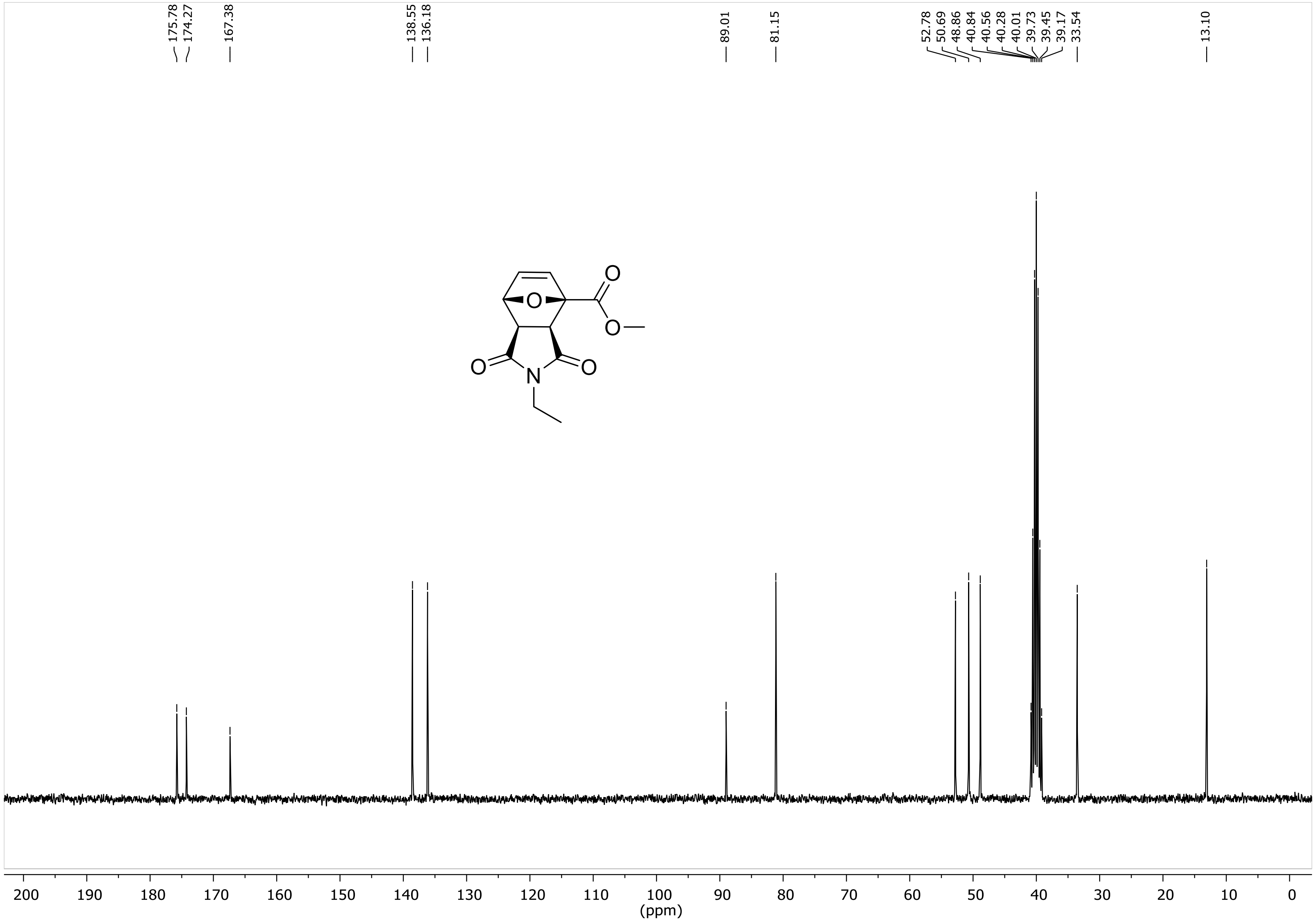

Figure S34. $^{13}C\{^{1}H\}$ NMR spectrum (75 MHz, DMSO-d_6) of *exo*-**1b**.

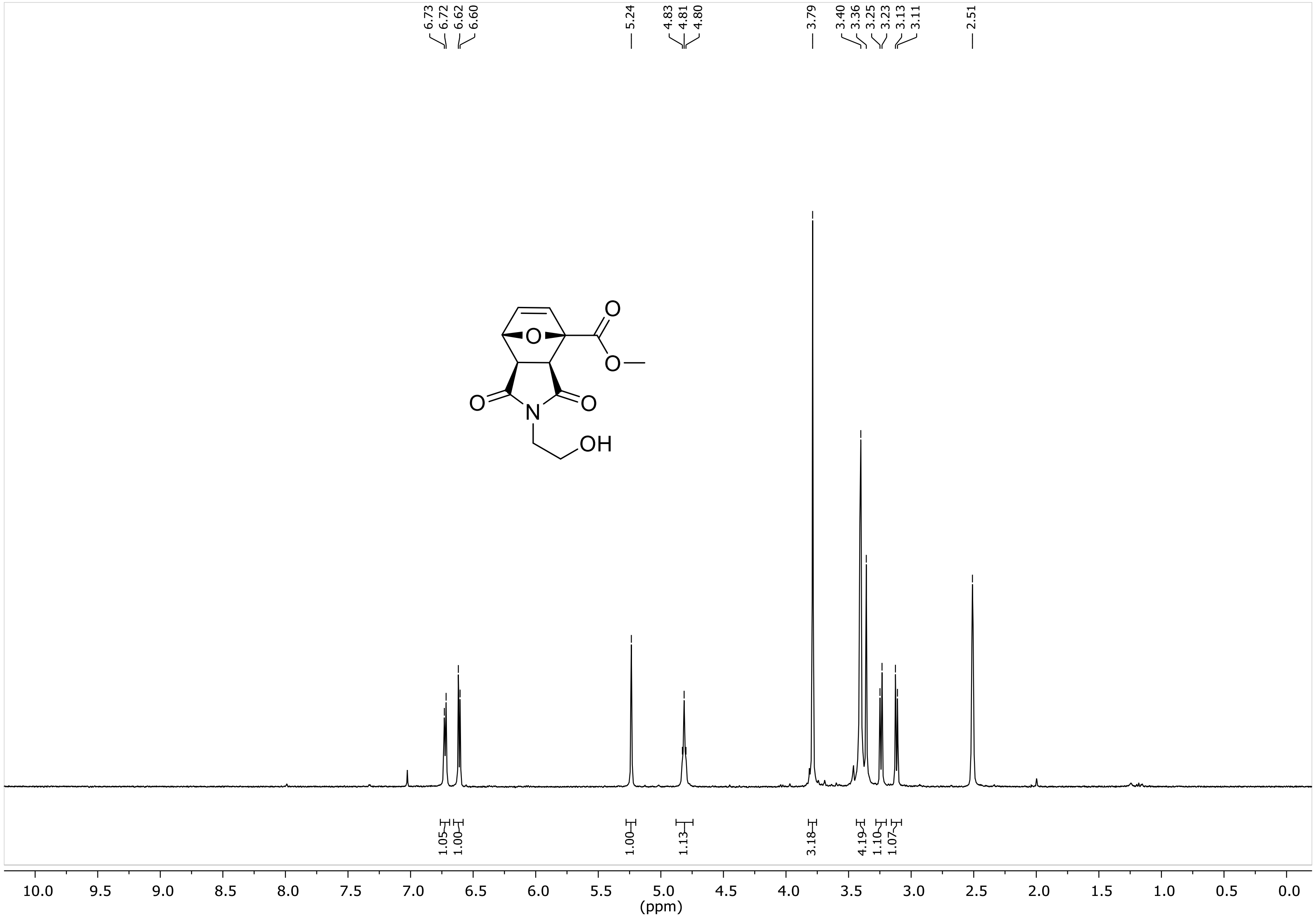

Figure S35. ^{1}H NMR spectrum (400 MHz, DMSO-d_6) of *exo*-**1c**.

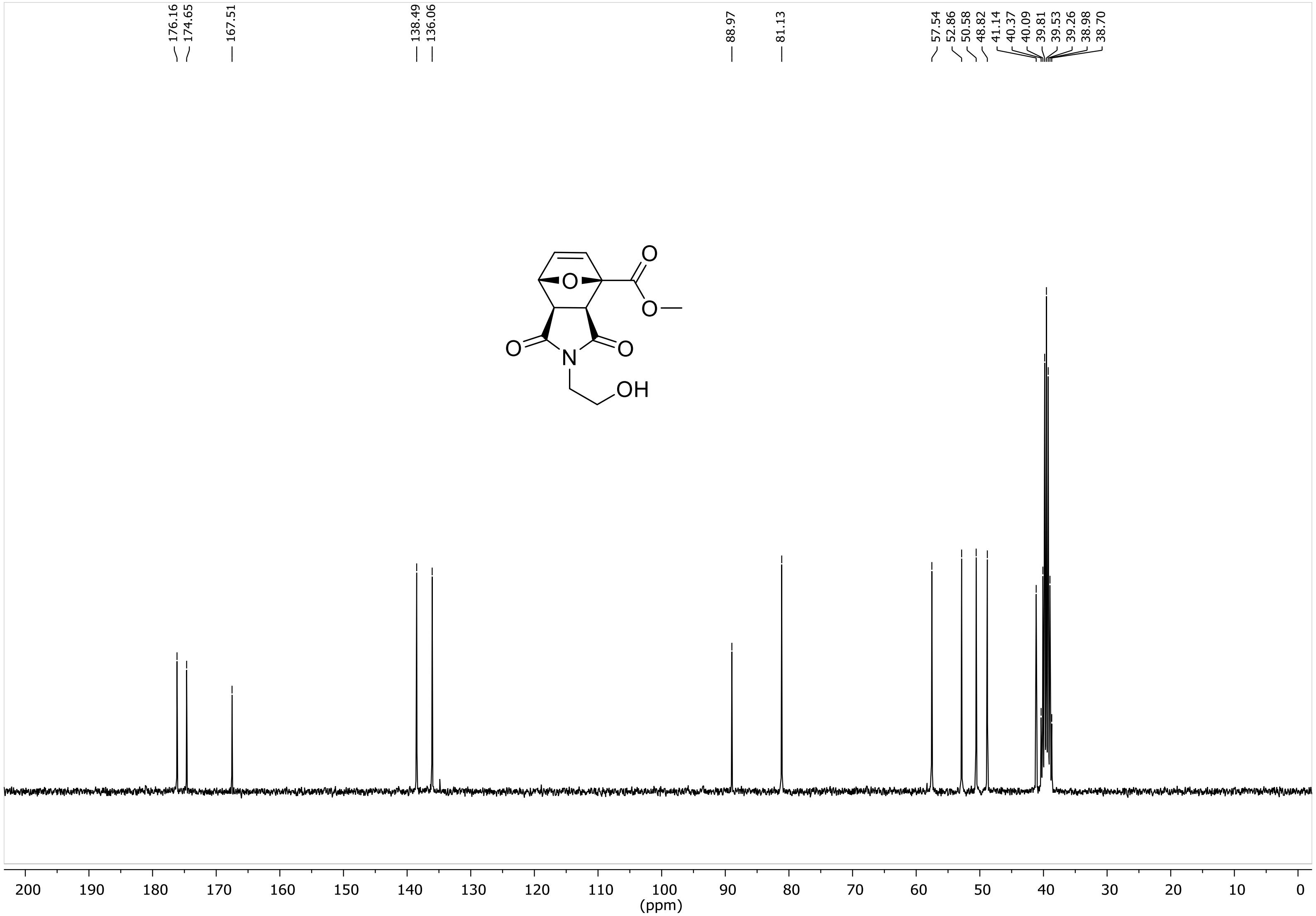

Figure S36. $^{13}C\{^{1}H\}$ NMR spectrum (75 MHz, DMSO-d_6) of *exo*-**1c**.

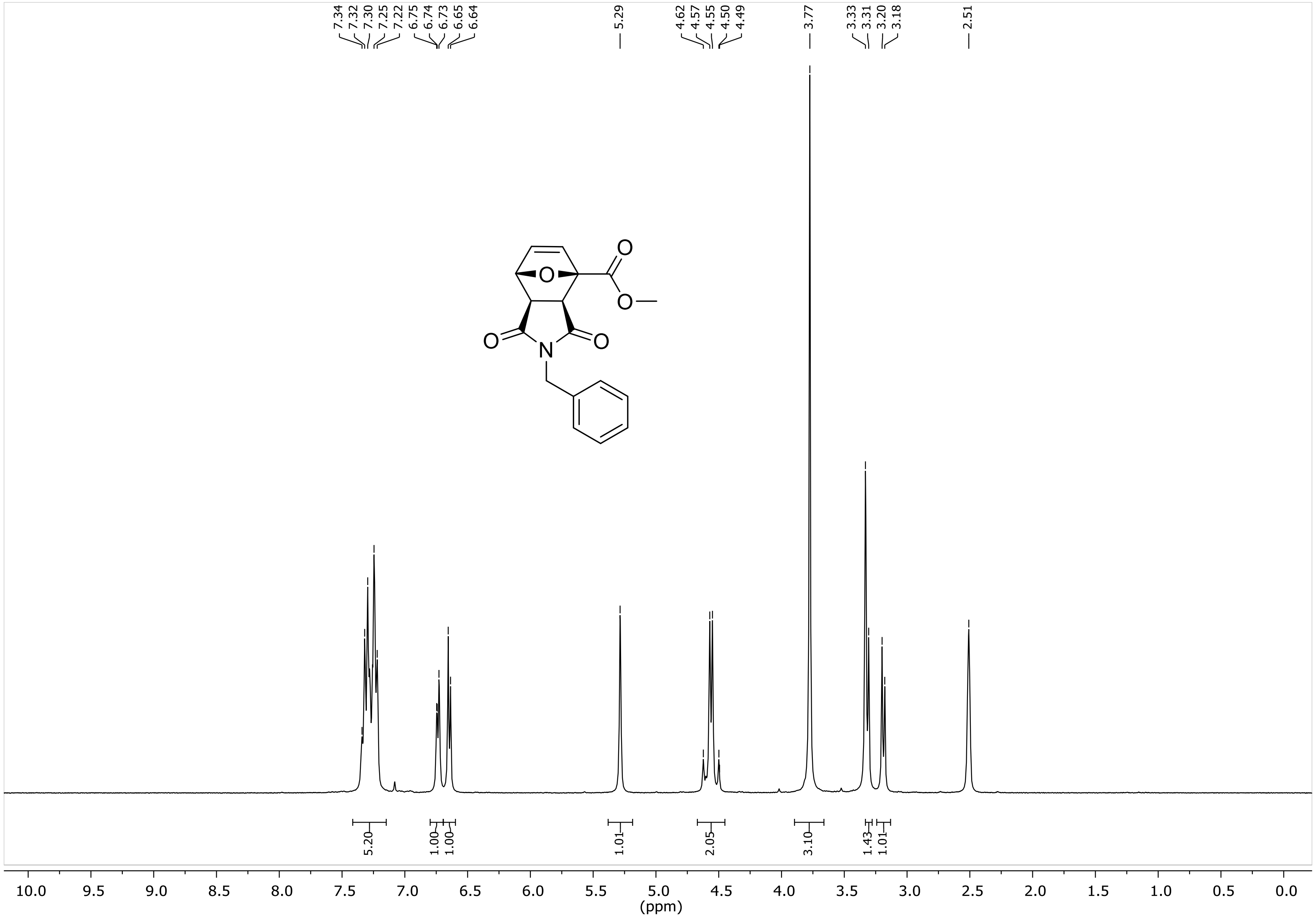

Figure S37. ^{1}H NMR spectrum (300 MHz, DMSO-d_6) of *exo*-**1d**.

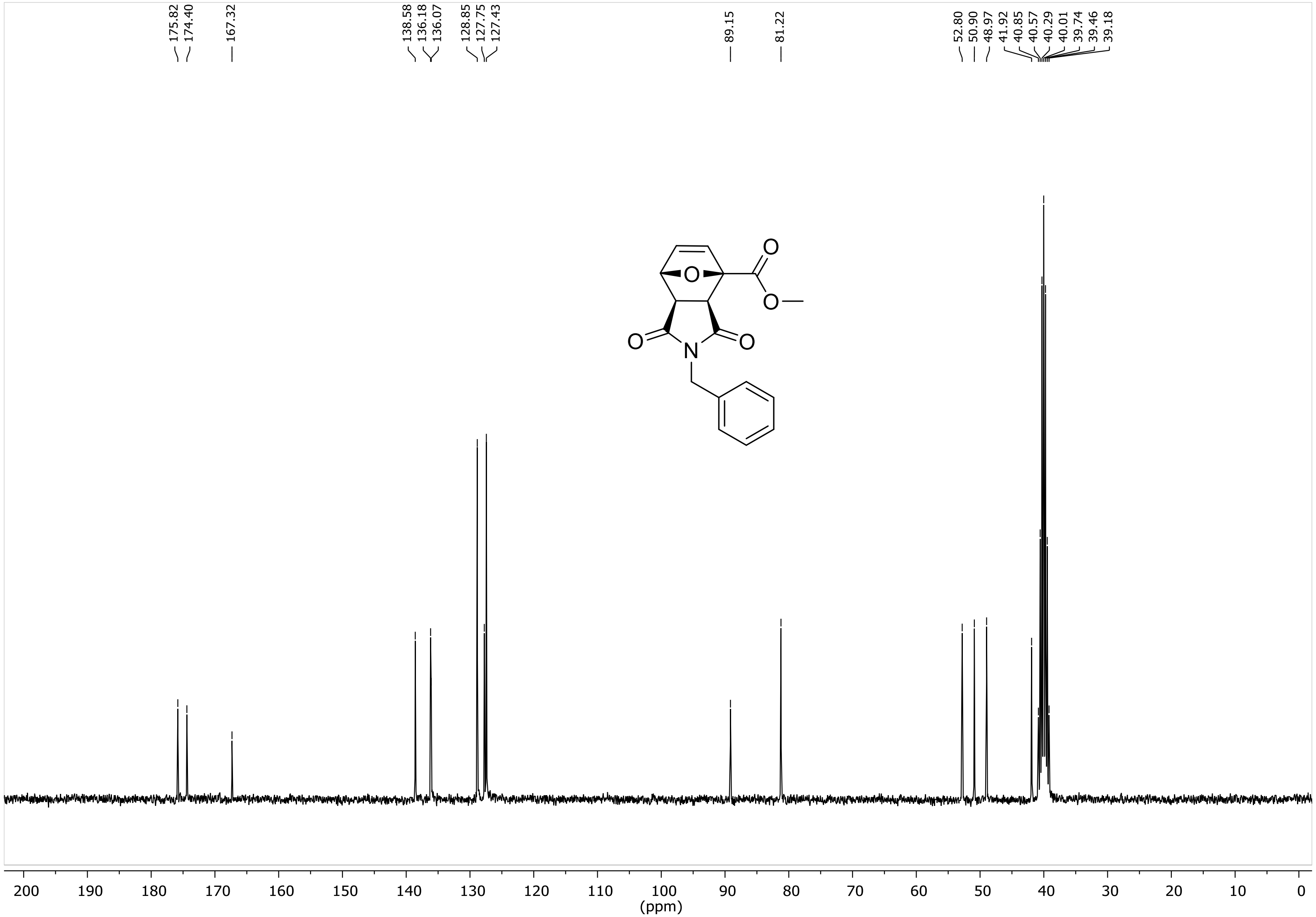

Figure S38. $^{13}C\{^{1}H\}$ NMR spectrum (75 MHz, DMSO-d_6) of *exo*-**1d**.

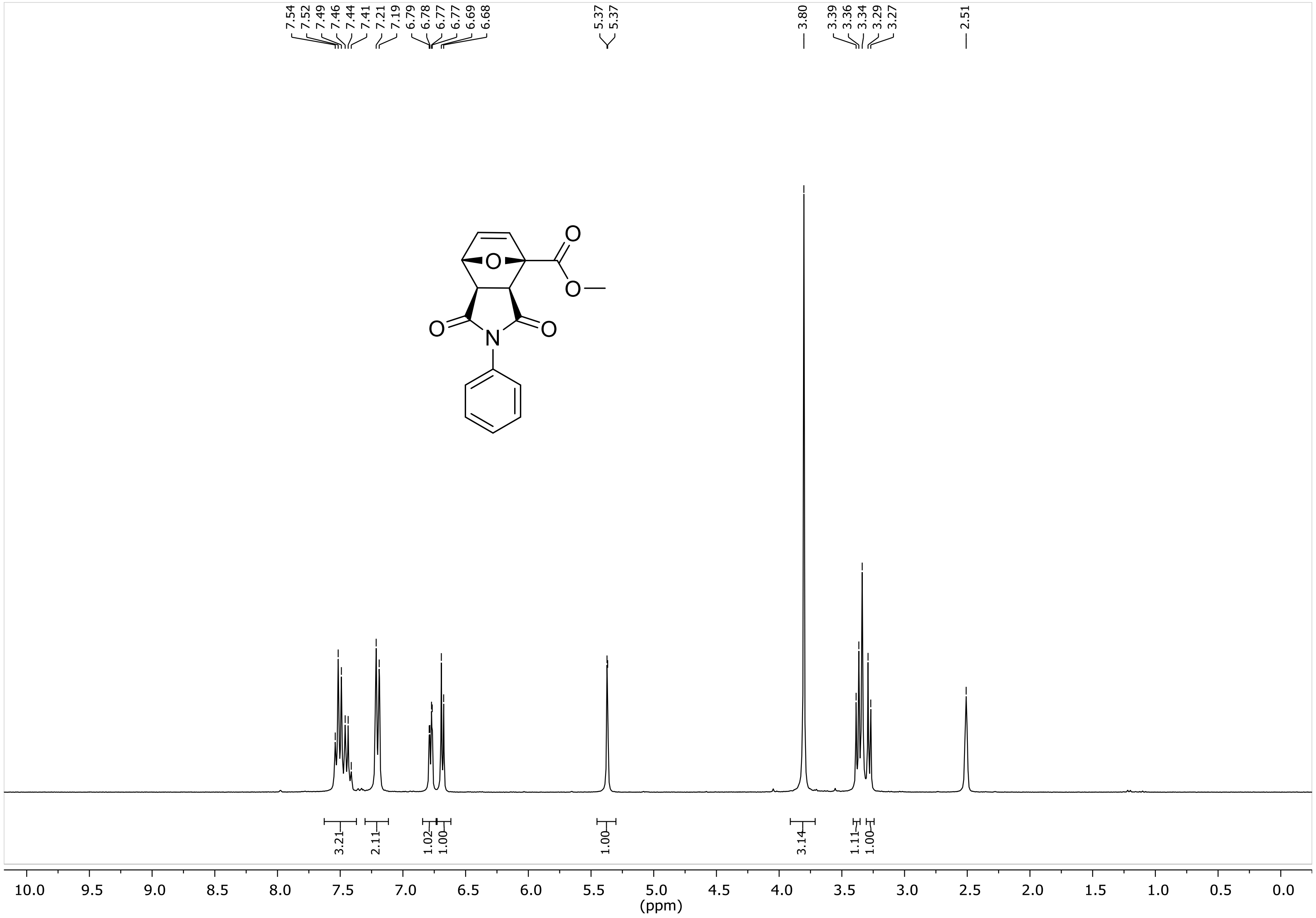

Figure S39. ^{1}H NMR spectrum (300 MHz, DMSO-d_6) of *exo*-**1e**.

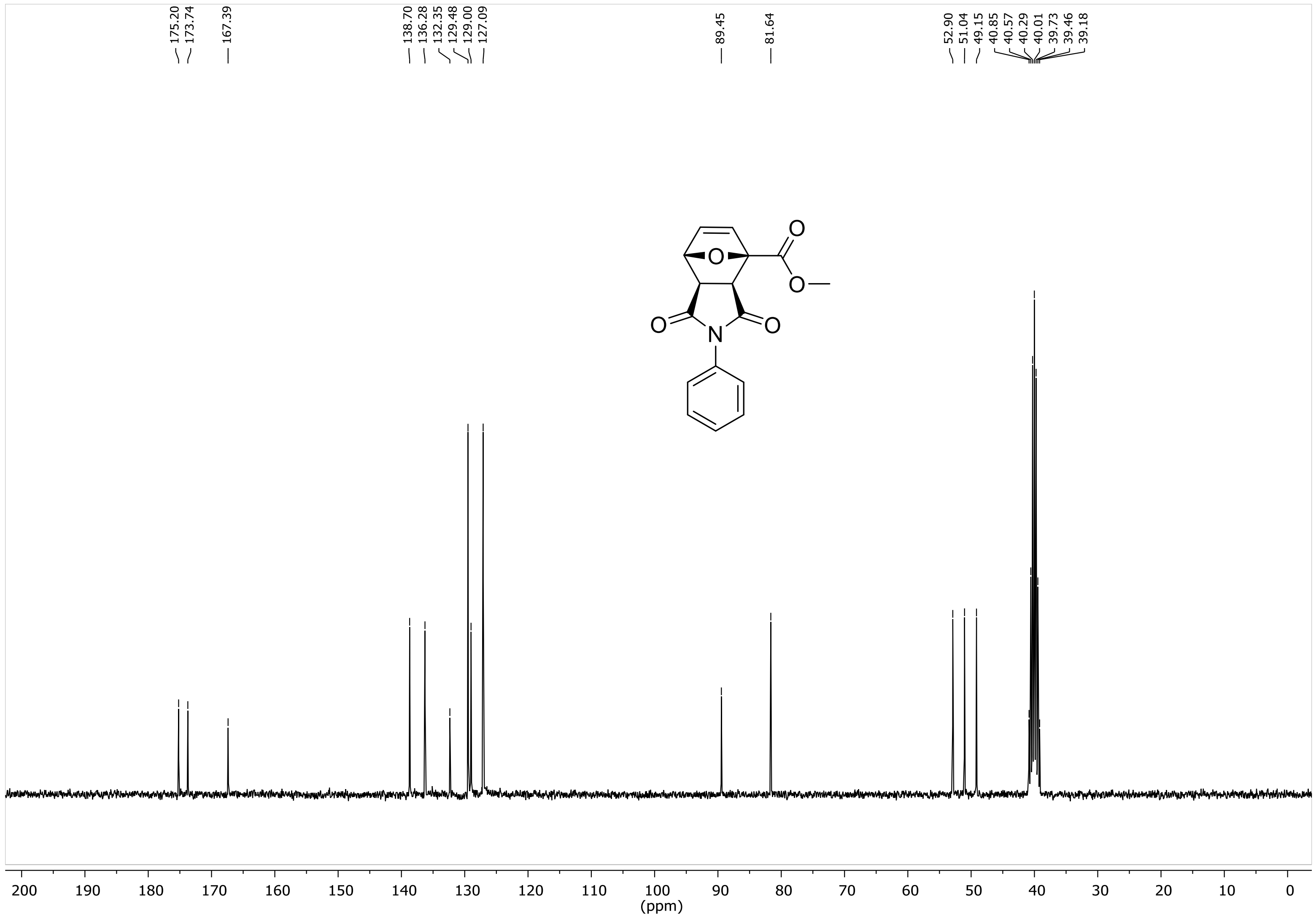

Figure S40. $^{13}C\{^{1}H\}$ NMR spectrum (75 MHz, DMSO-d_6) of *exo*-**1e**.

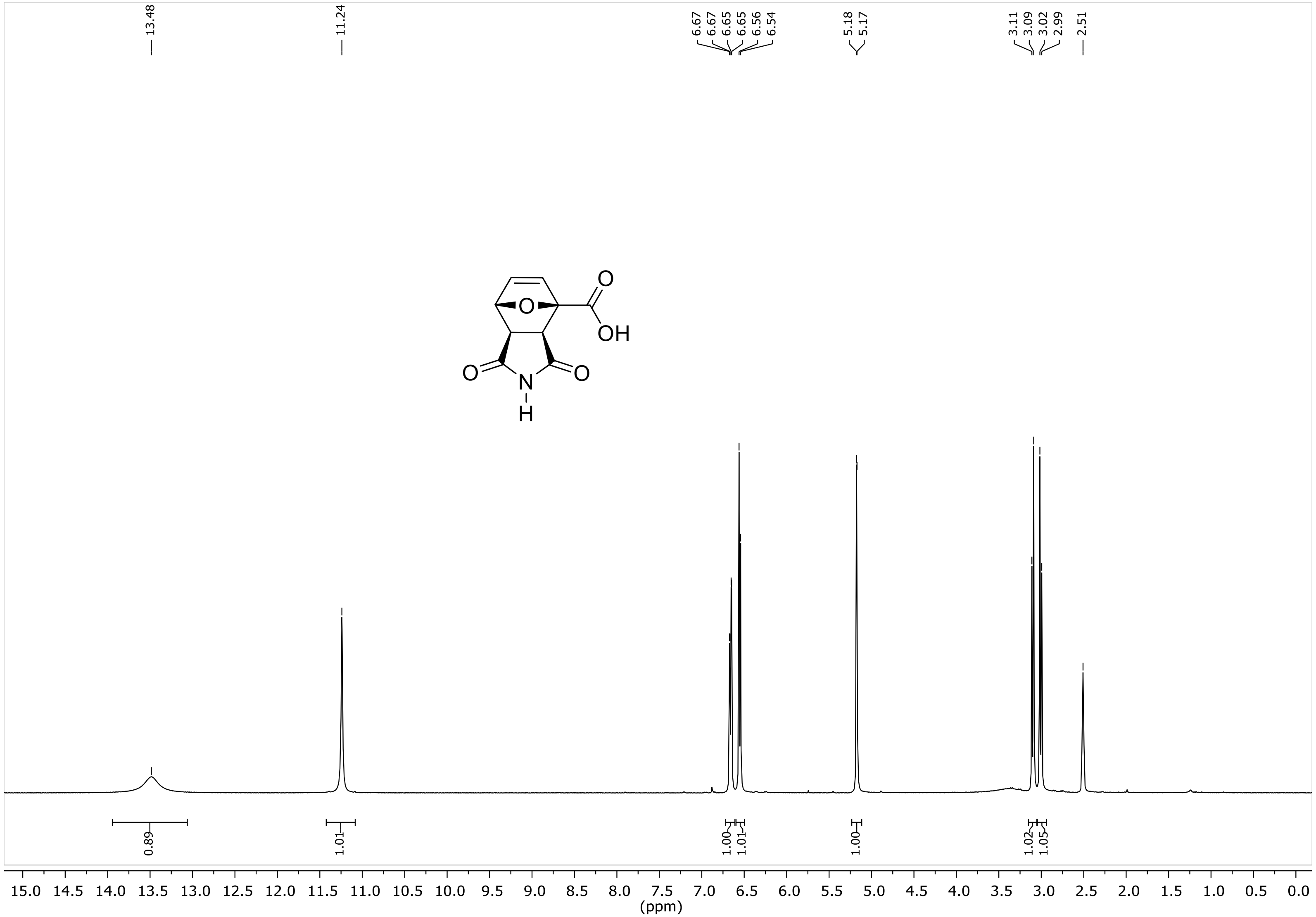

Figure S41. ^{1}H NMR spectrum (300 MHz, DMSO-d_6) of *exo*-**2a**.

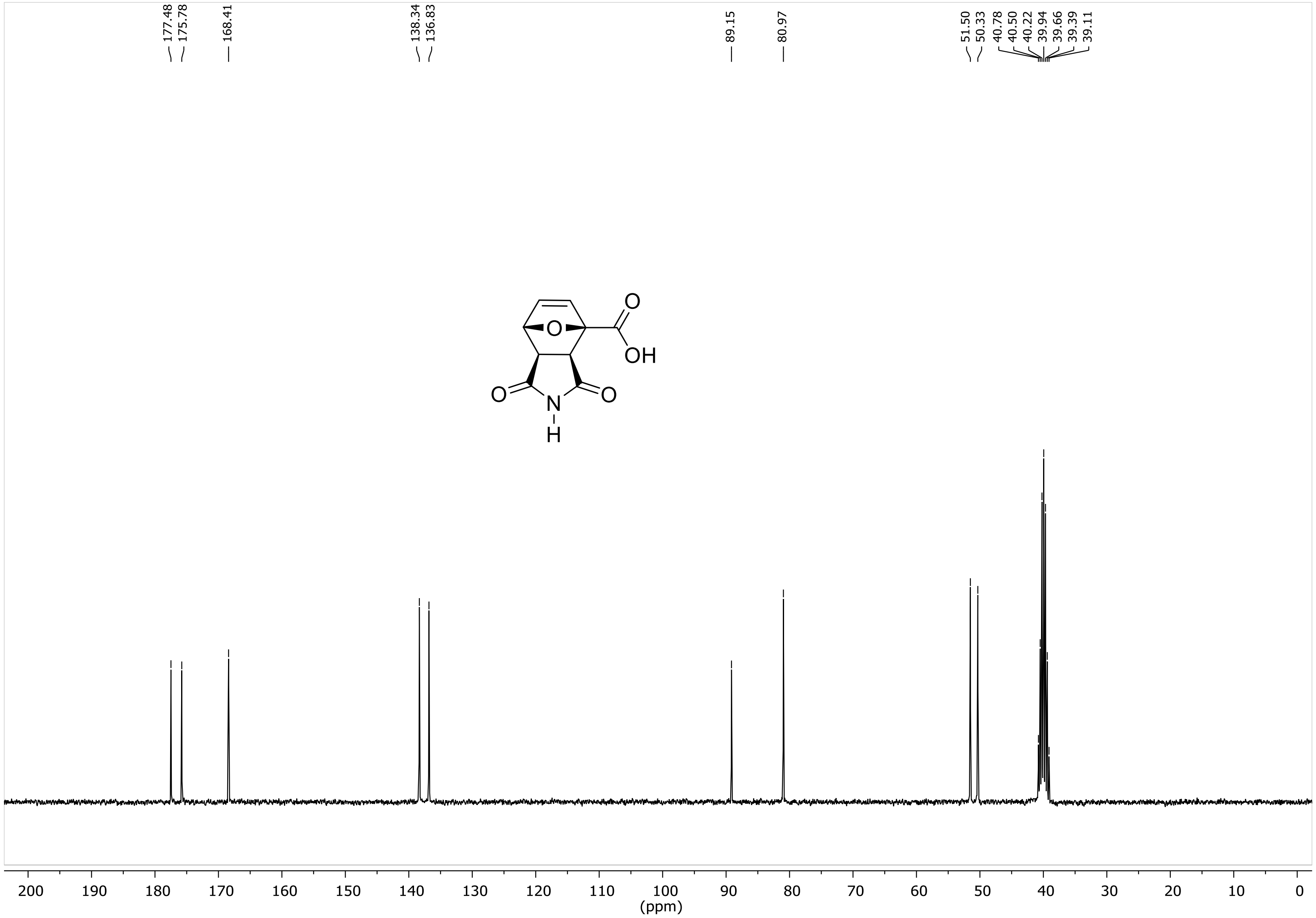

Figure S42. $^{13}C\{^{1}H\}$ NMR spectrum (75 MHz, DMSO-d_6) of *exo*-**2a**.

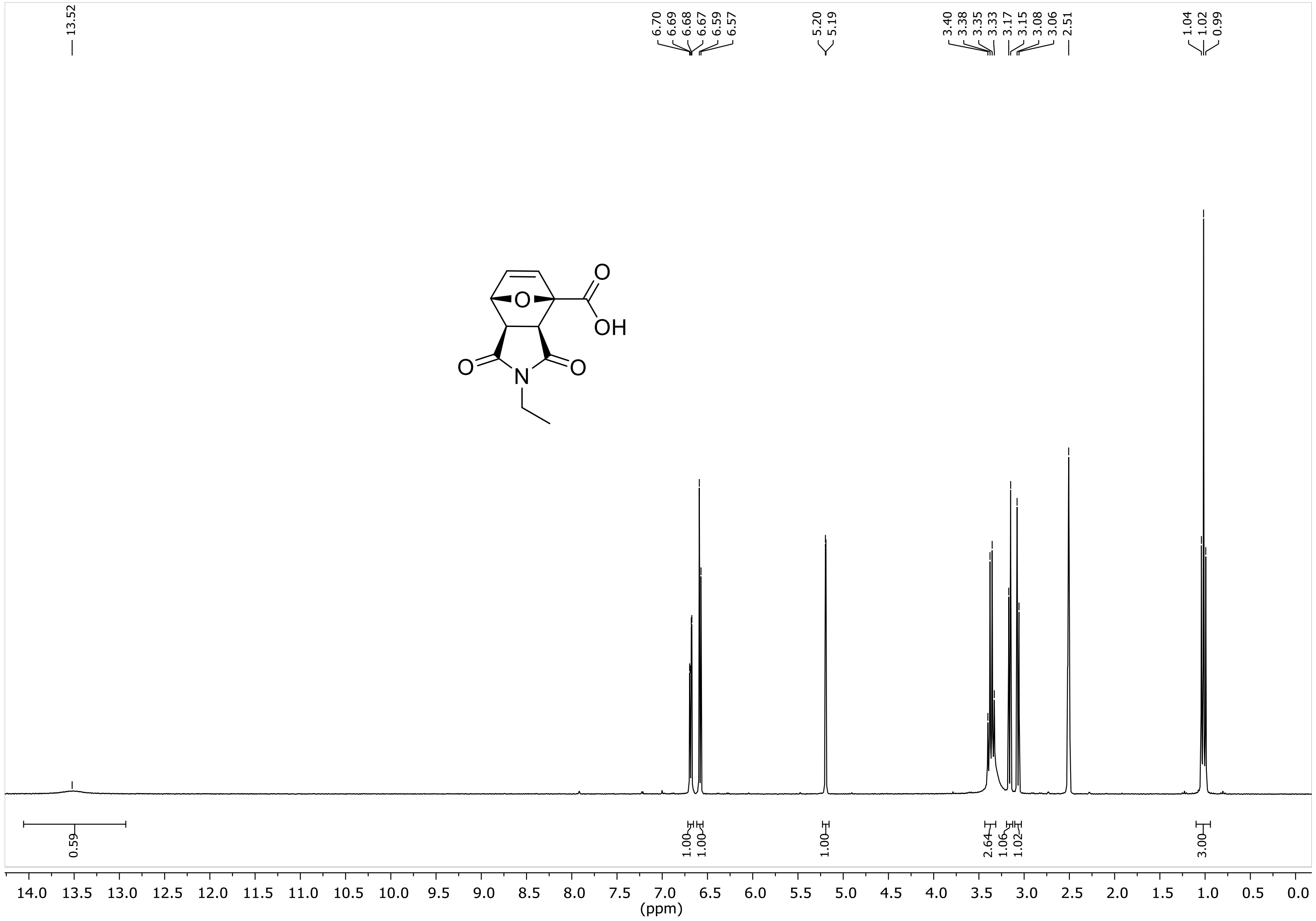

Figure S43. ^{1}H NMR spectrum (300 MHz, DMSO-d_6) of *exo*-**2b**.

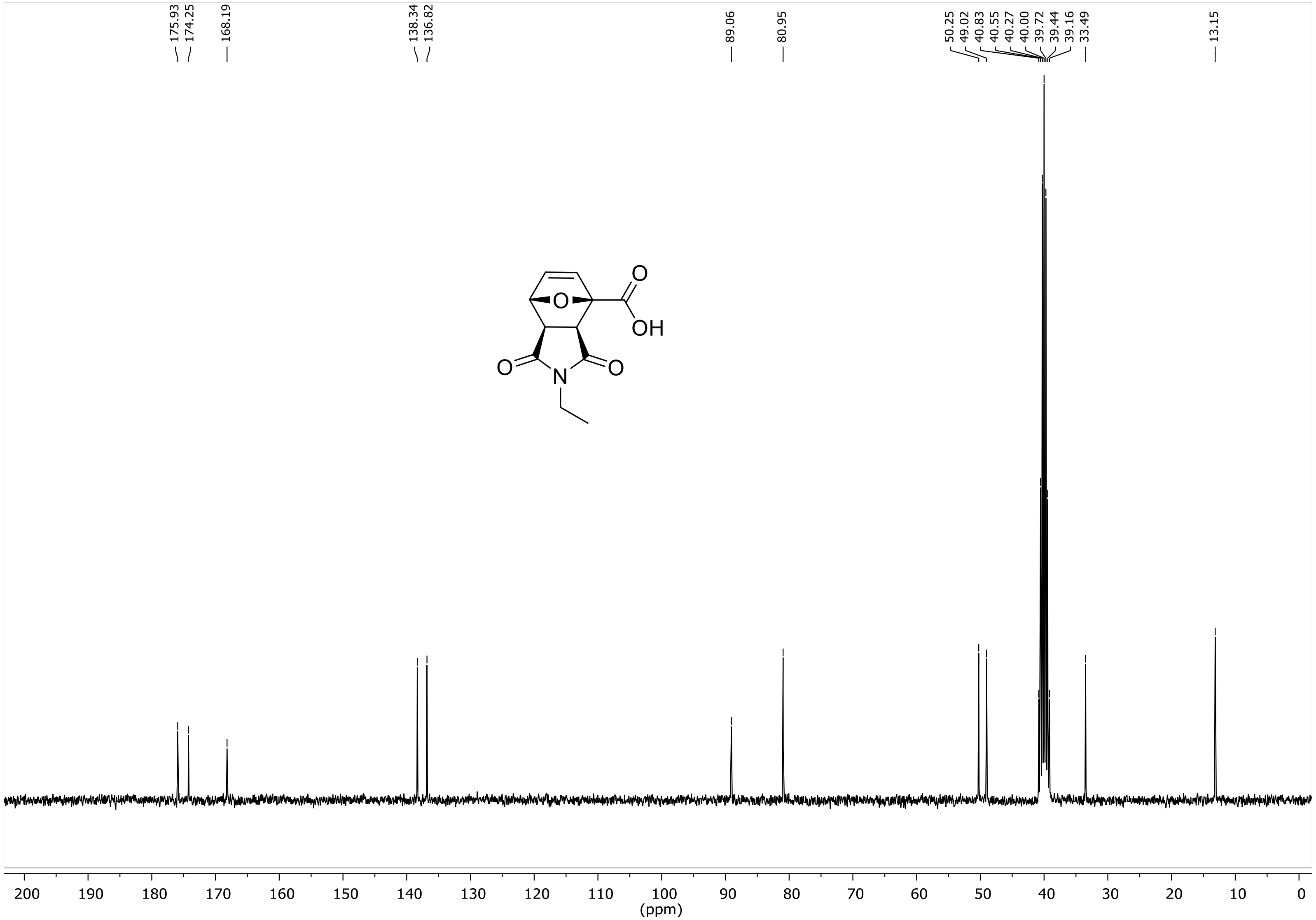

Figure S44. $^{13}C\{^1H\}$ NMR spectrum (75 MHz, DMSO-d_6) of *exo*-**2b**.

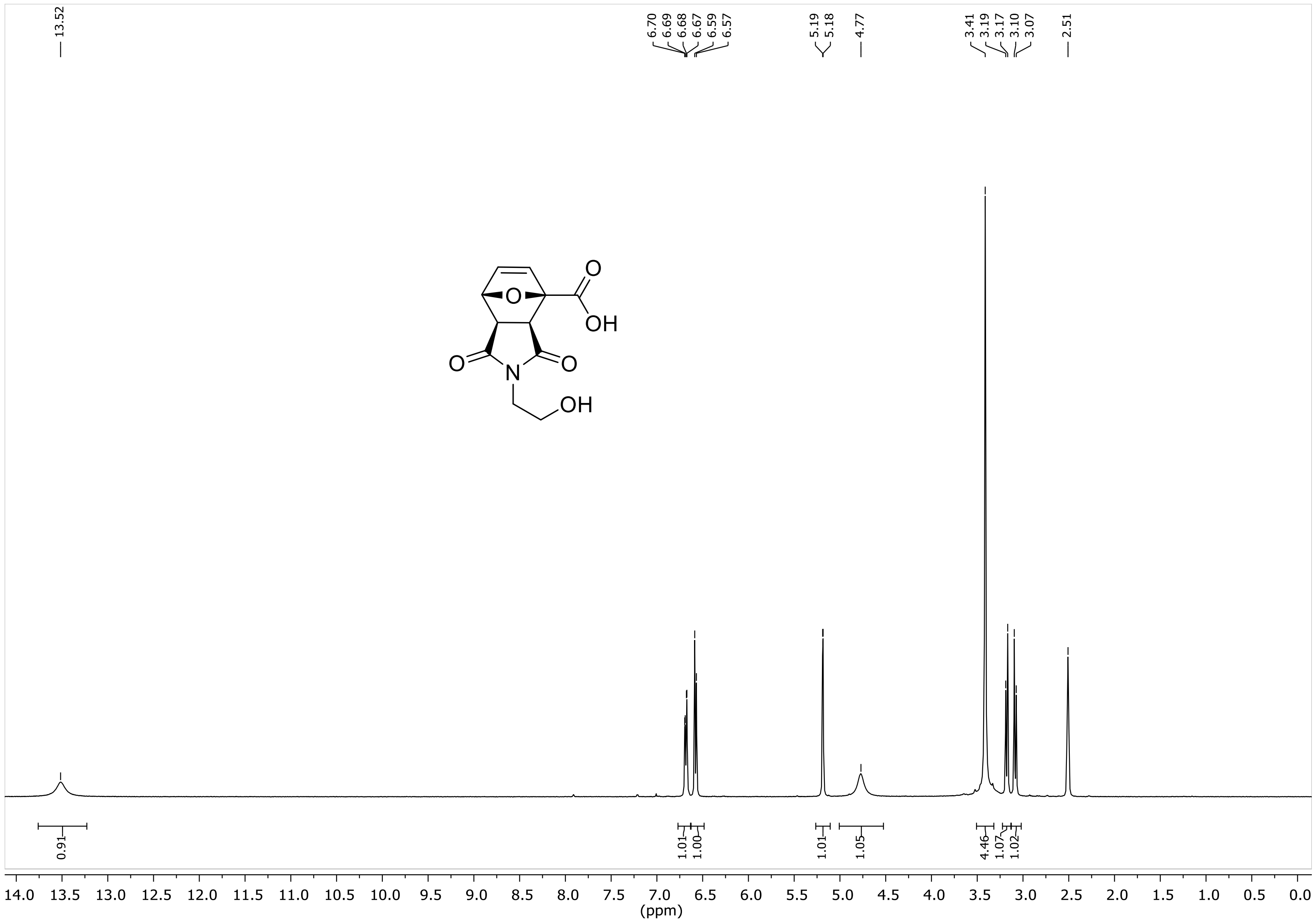

Figure S45. ^{1}H NMR spectrum (300 MHz, DMSO-d_6) of *exo*-**2c**.

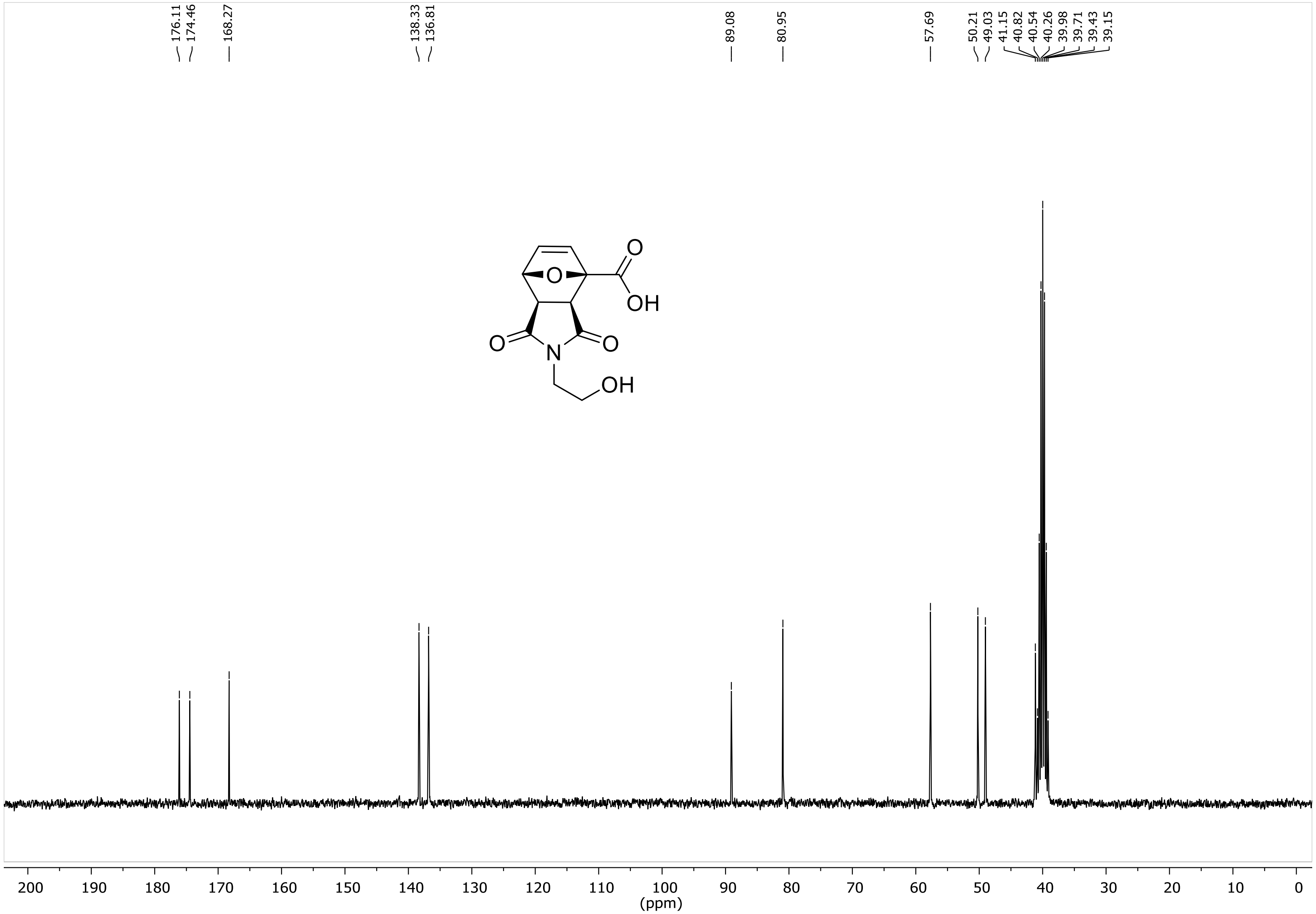

Figure S46. $^{13}C\{^{1}H\}$ NMR spectrum (75 MHz, DMSO-d_6) of *exo*-**2c**.

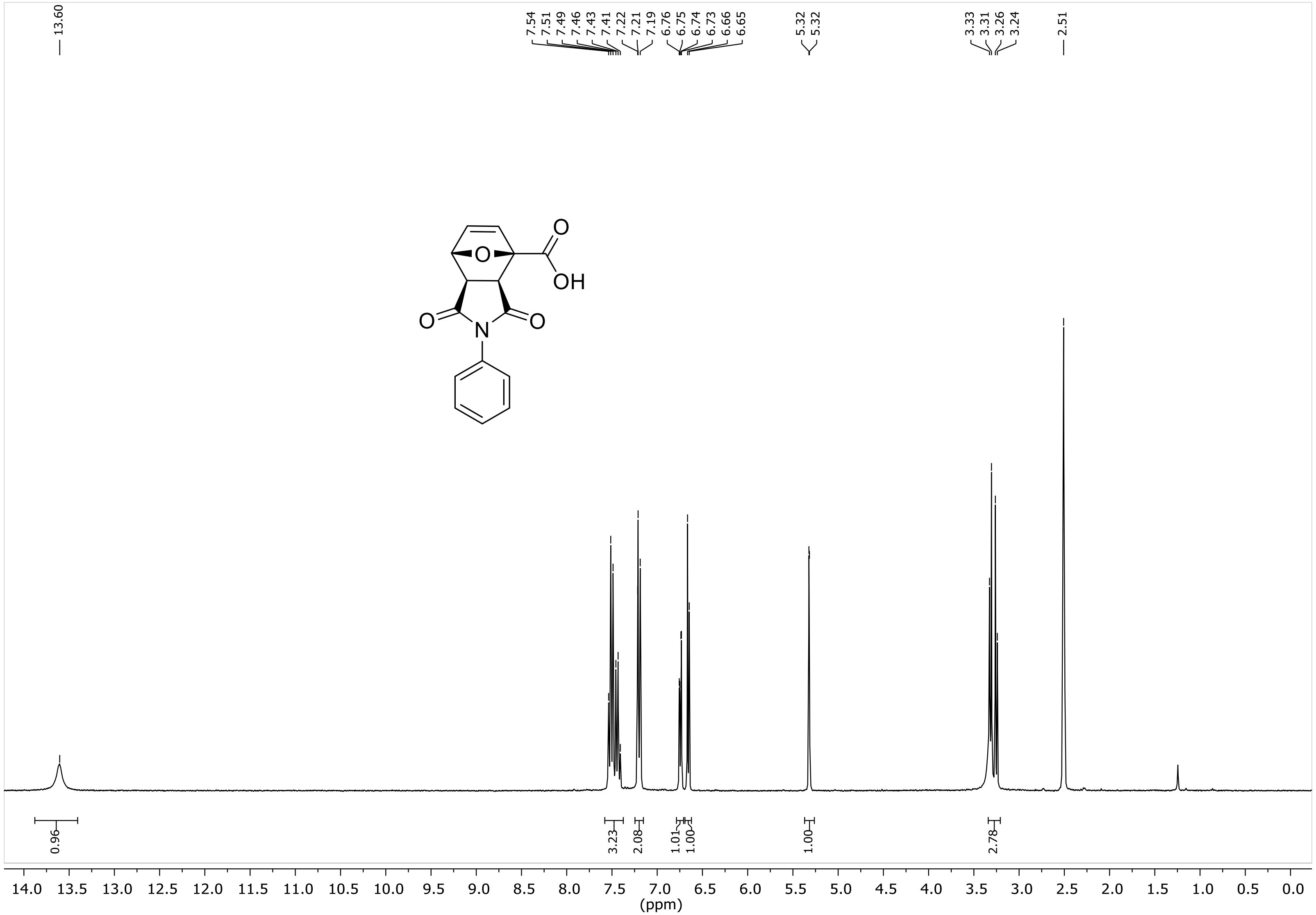

Figure S47. ^{1}H NMR spectrum (300 MHz, DMSO-d_6) of *exo*-**2e**.

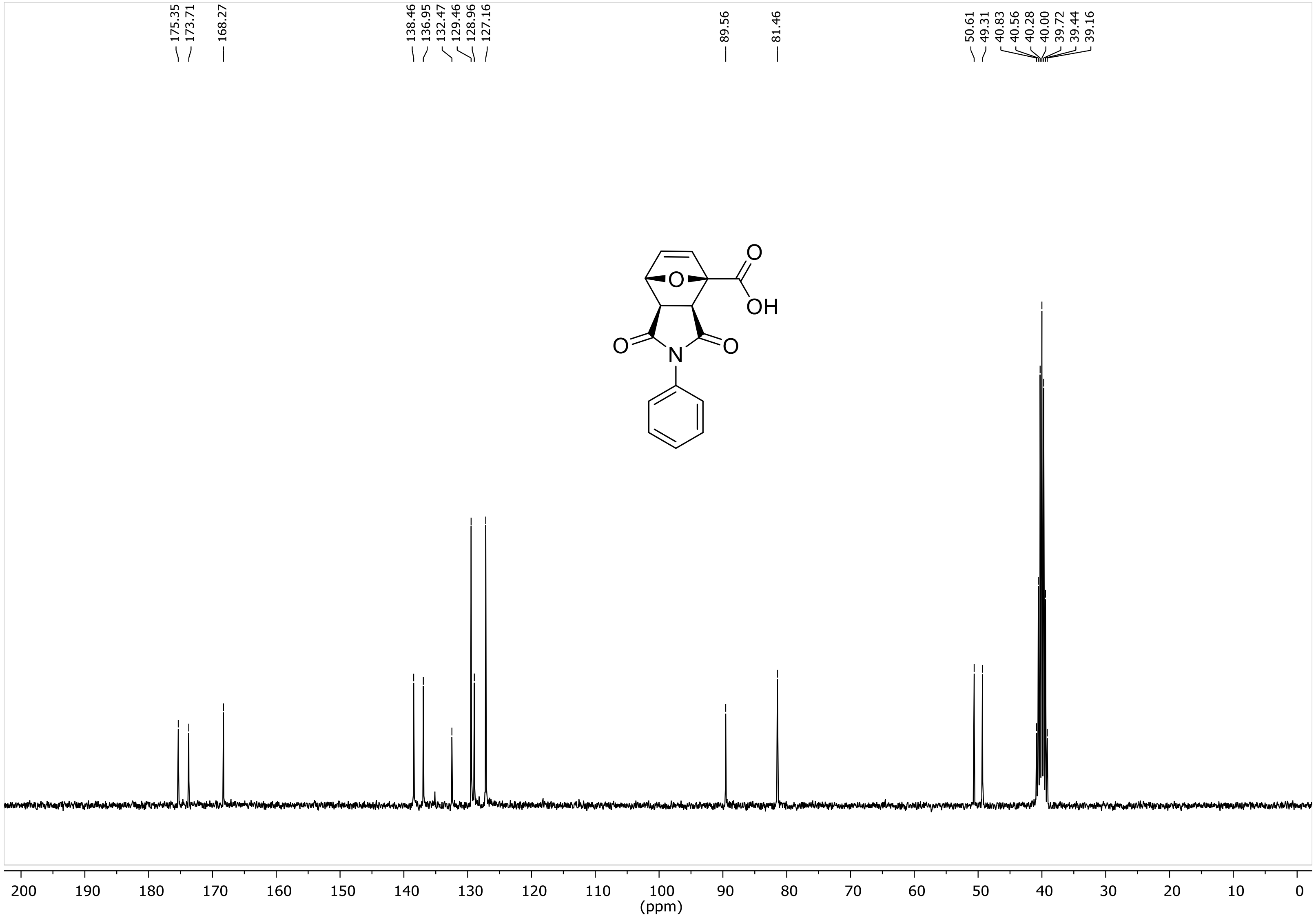

Figure S48. $^{13}C\{^{1}H\}$ NMR spectrum (75 MHz, DMSO-d_6) of *exo*-**2e**.

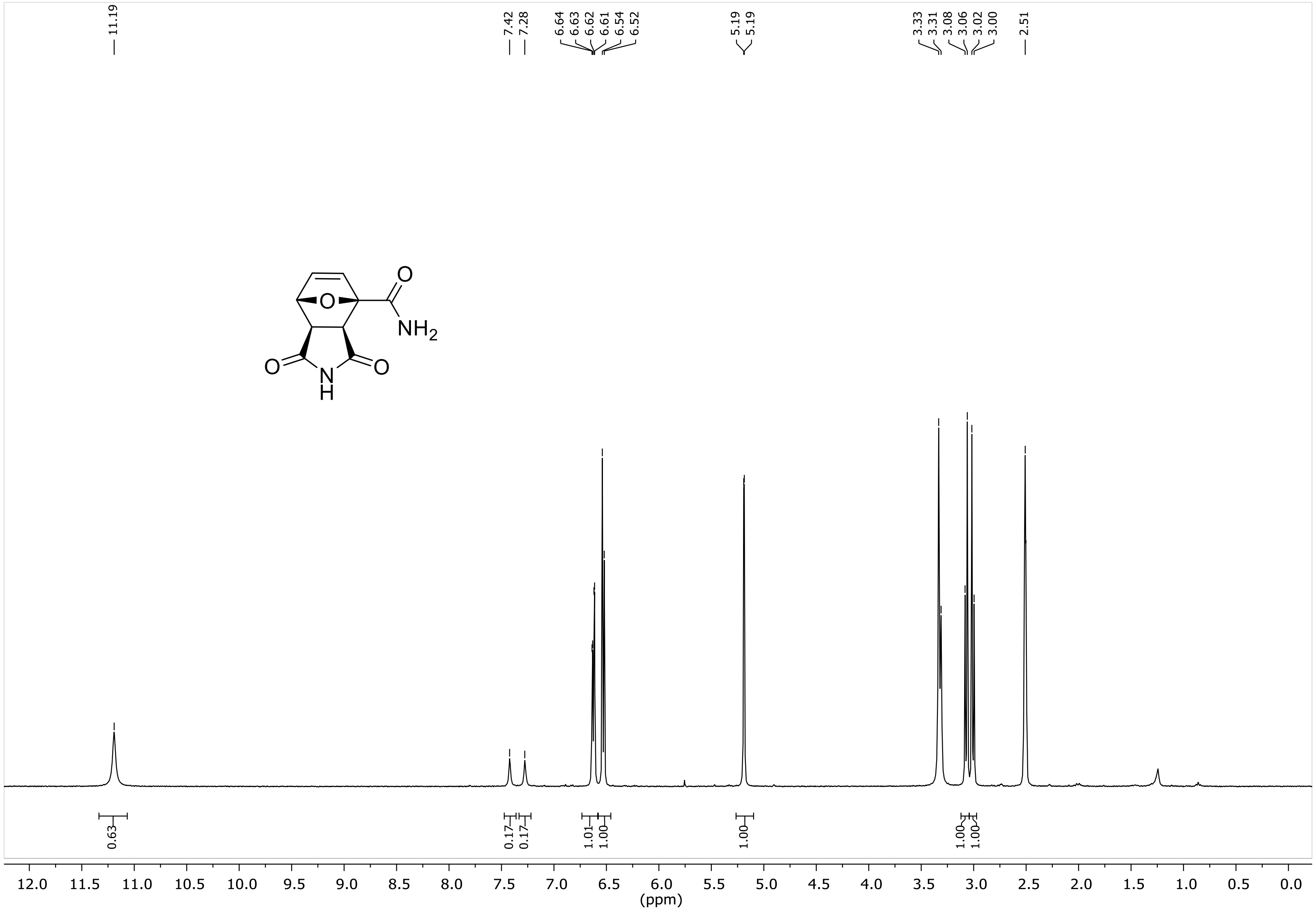

Figure S49. ^{1}H NMR spectrum (300 MHz, DMSO-d_6) of *exo*-**3a**.

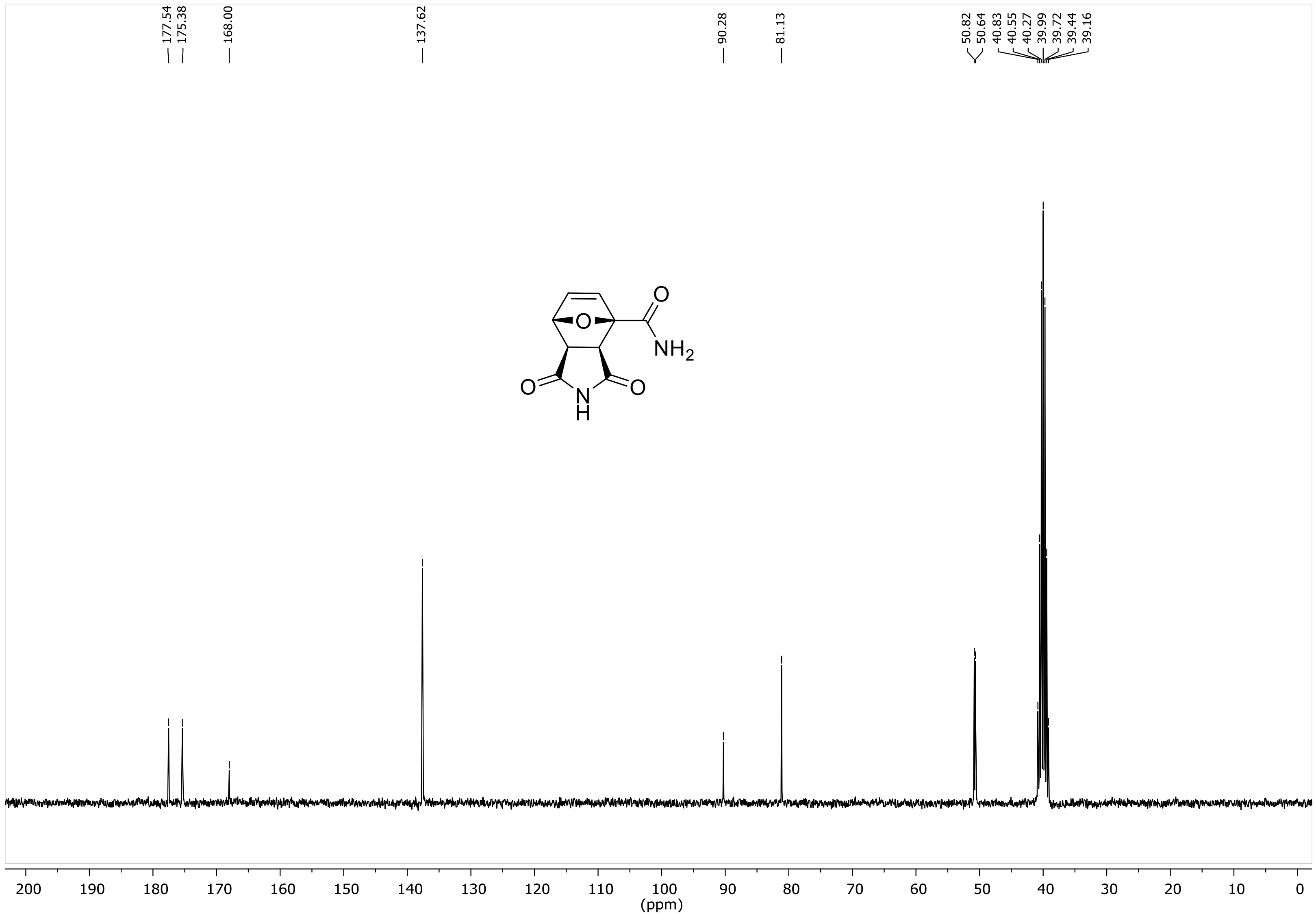

Figure S50. $^{13}C\{^{1}H\}$ NMR spectrum (75 MHz, DMSO-d_6) of *exo*-**3a**.

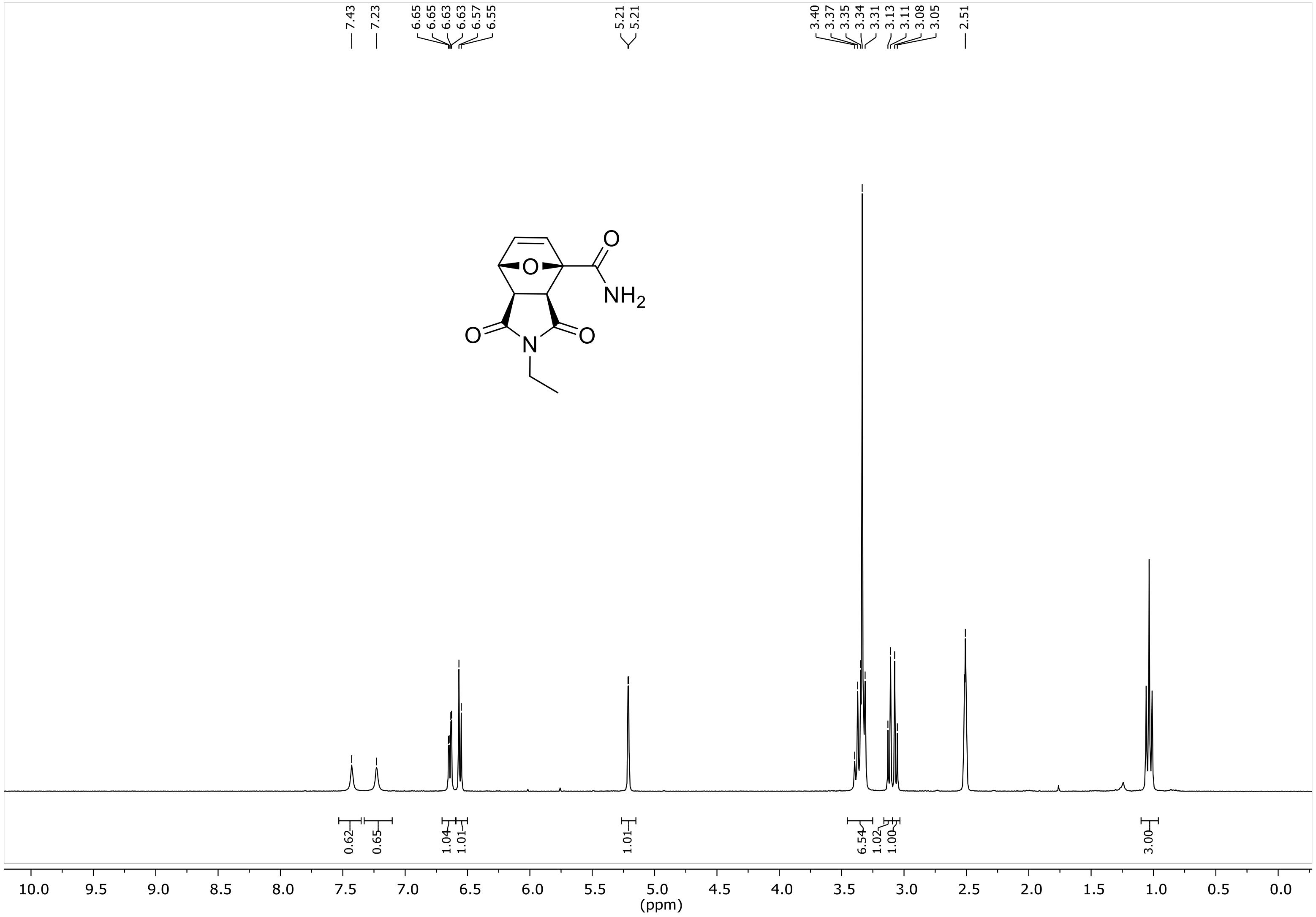

Figure S51. ^{1}H NMR spectrum (300 MHz, DMSO-d_6) of *exo*-**3b**.

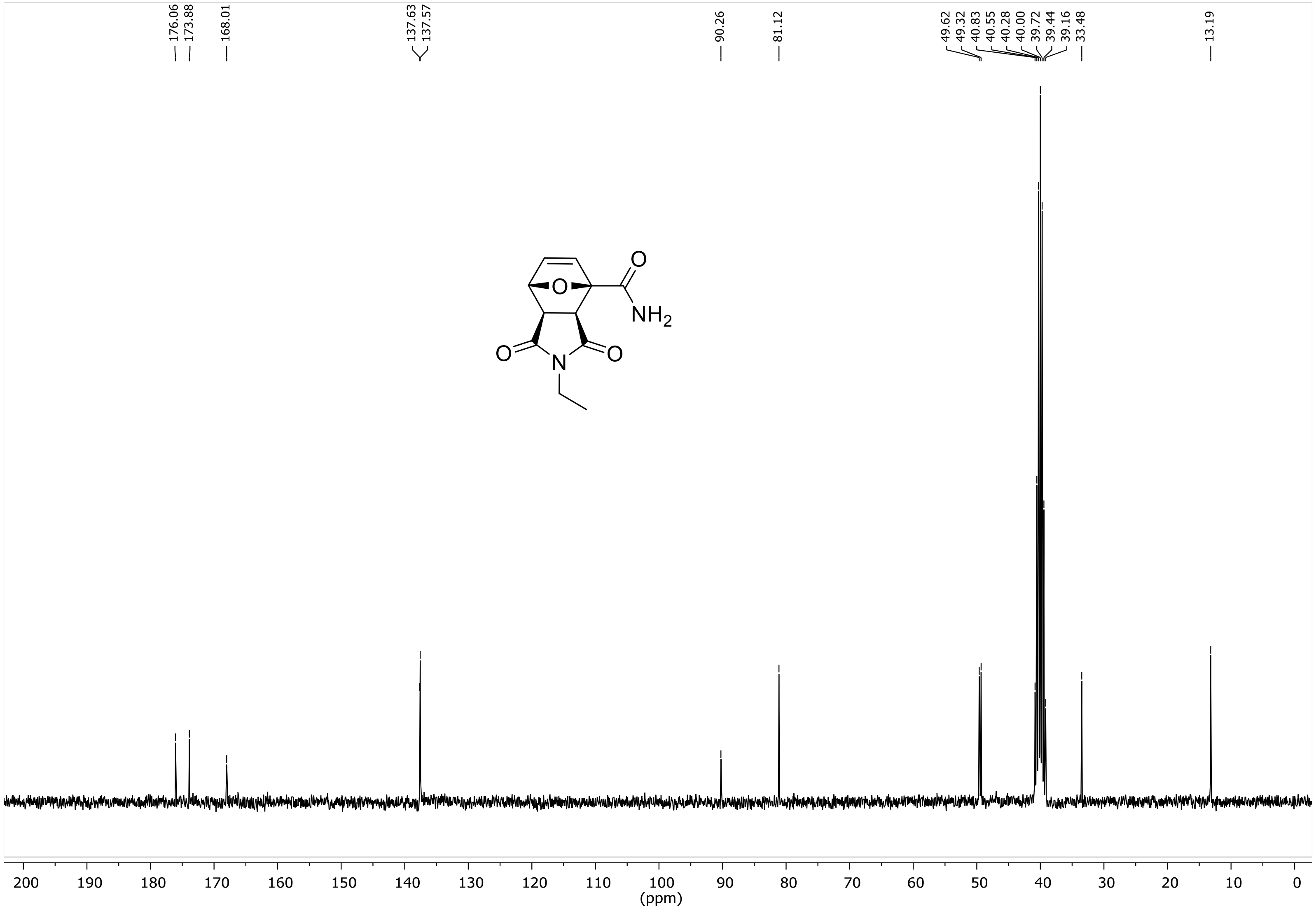

Figure S52. $^{13}C\{^{1}H\}$ NMR spectrum (75 MHz, DMSO-d_6) of *exo*-**3b**.

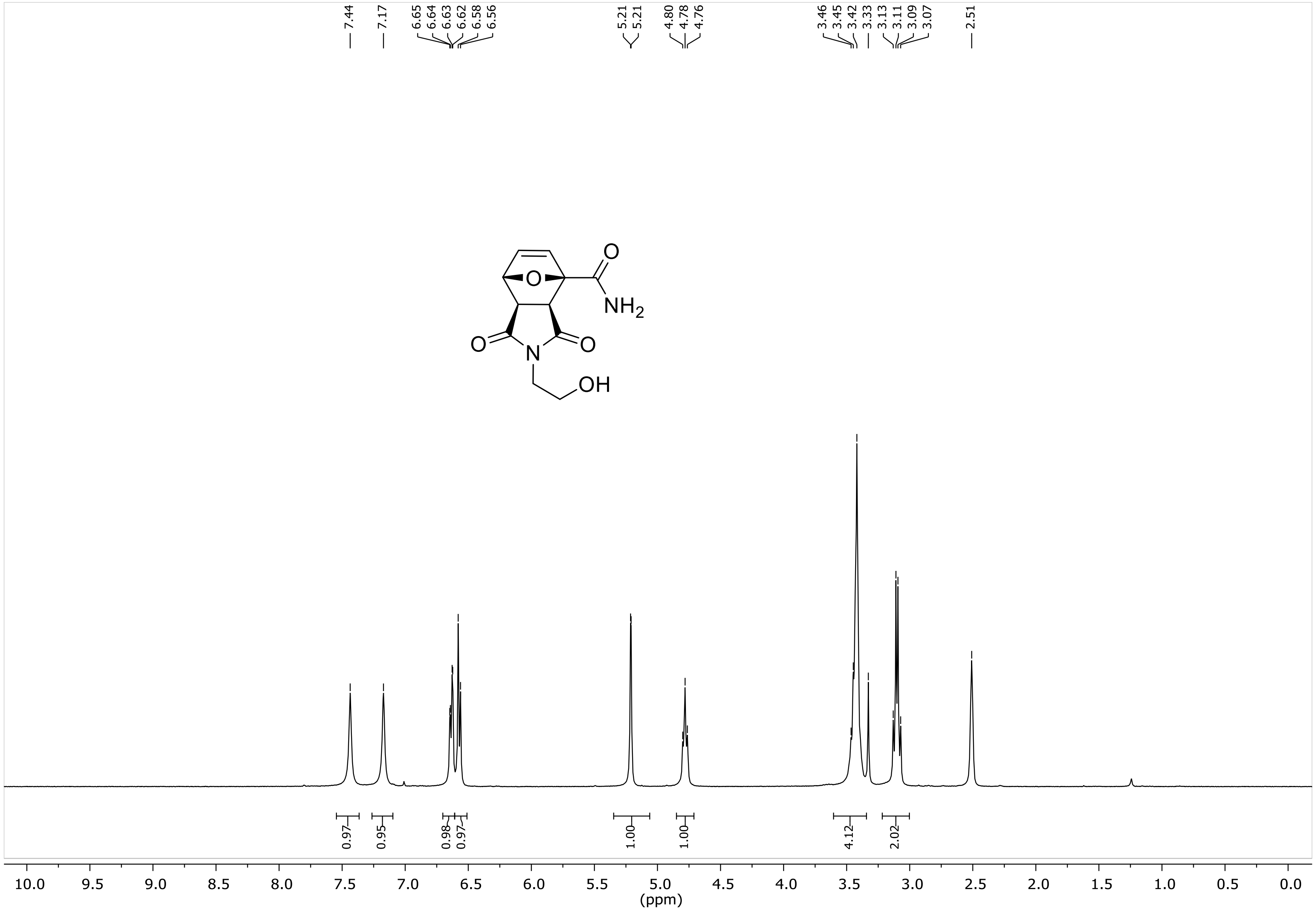

Figure S53. ^{1}H NMR spectrum (300 MHz, DMSO-d_6) of *exo*-**3c**.

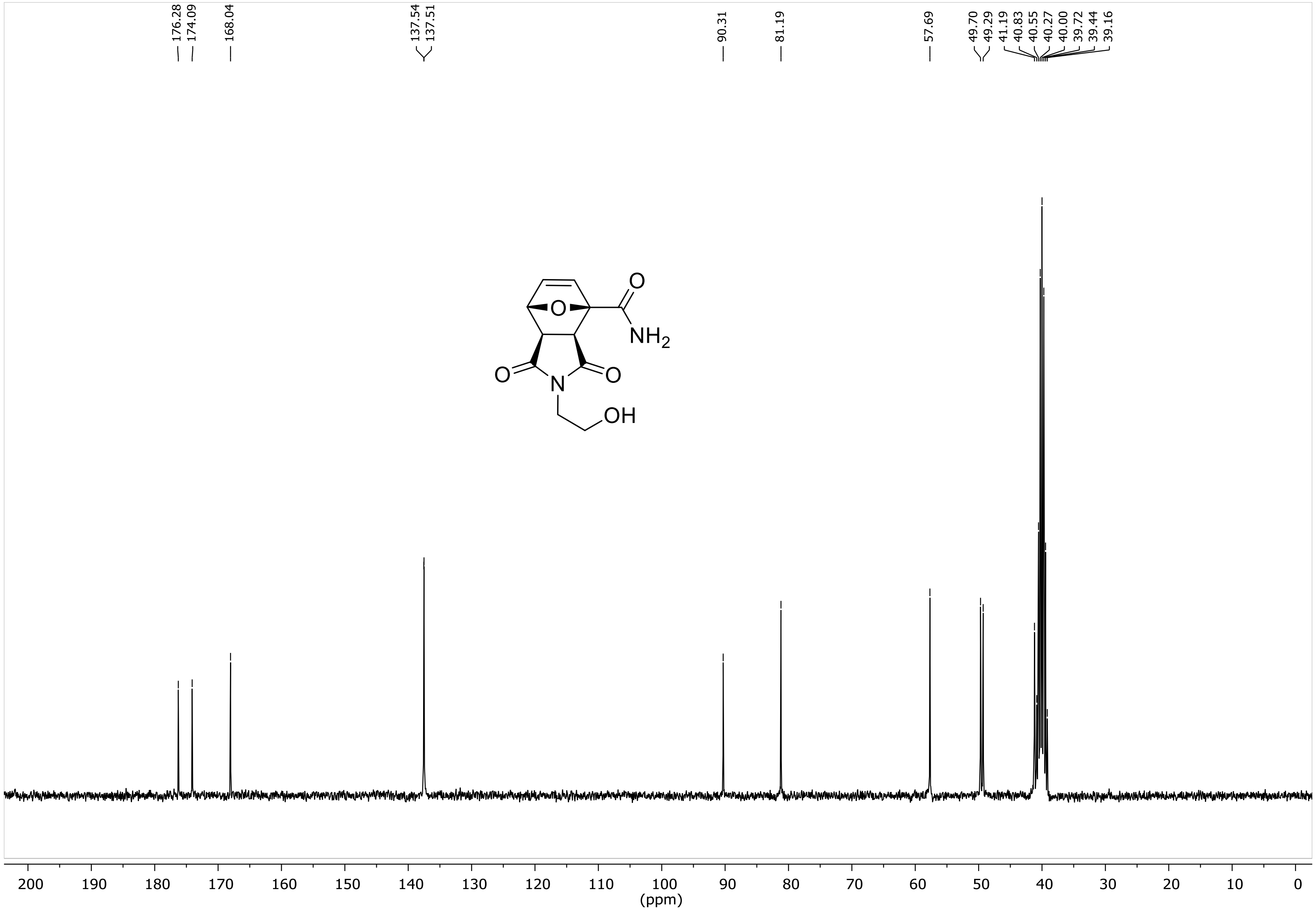

Figure S54. $^{13}C\{^{1}H\}$ NMR spectrum (75 MHz, DMSO-d_6) of *exo*-**3c**.

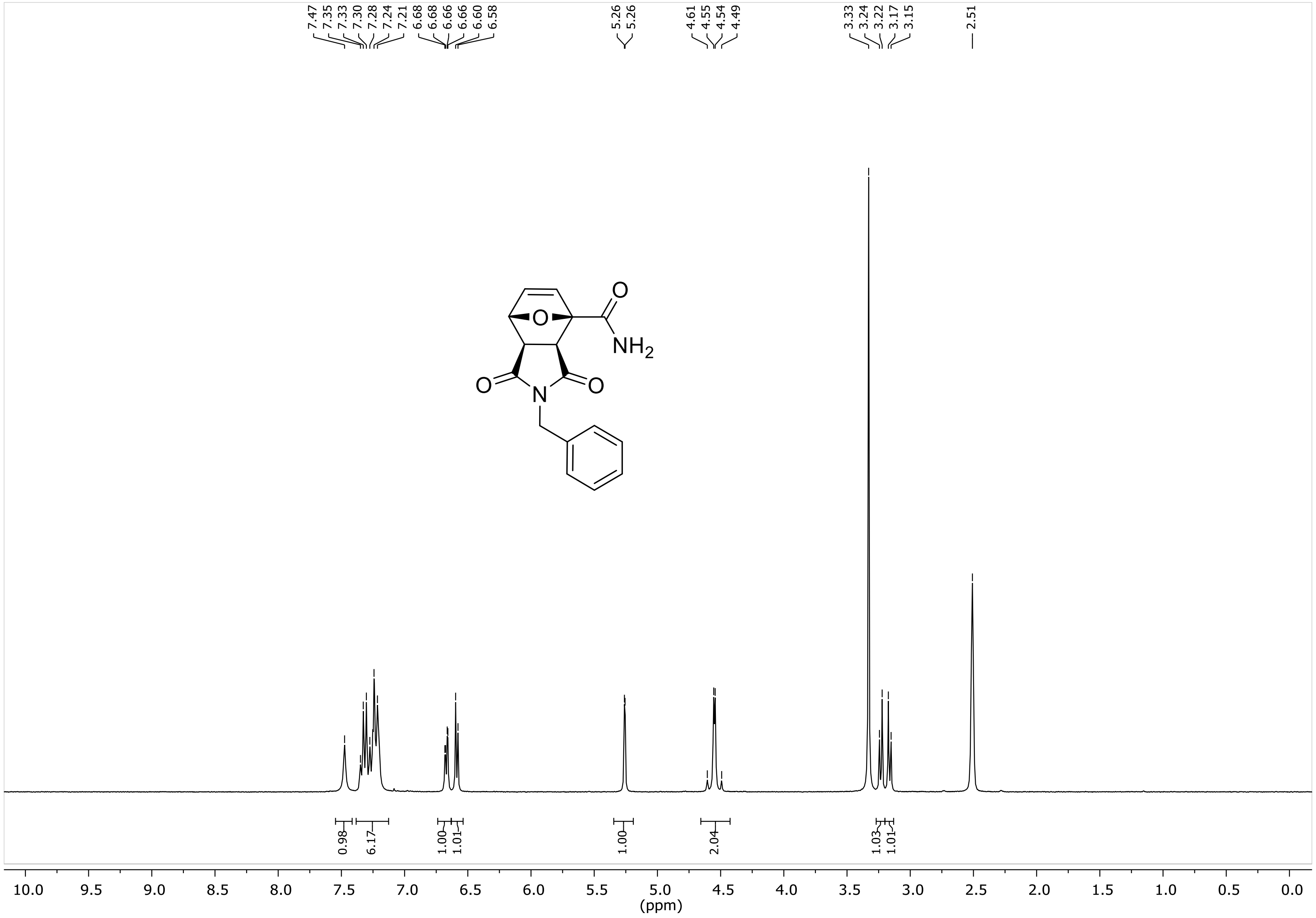

Figure S55. ^{1}H NMR spectrum (300 MHz, DMSO-d_6) of *exo*-**3d**.

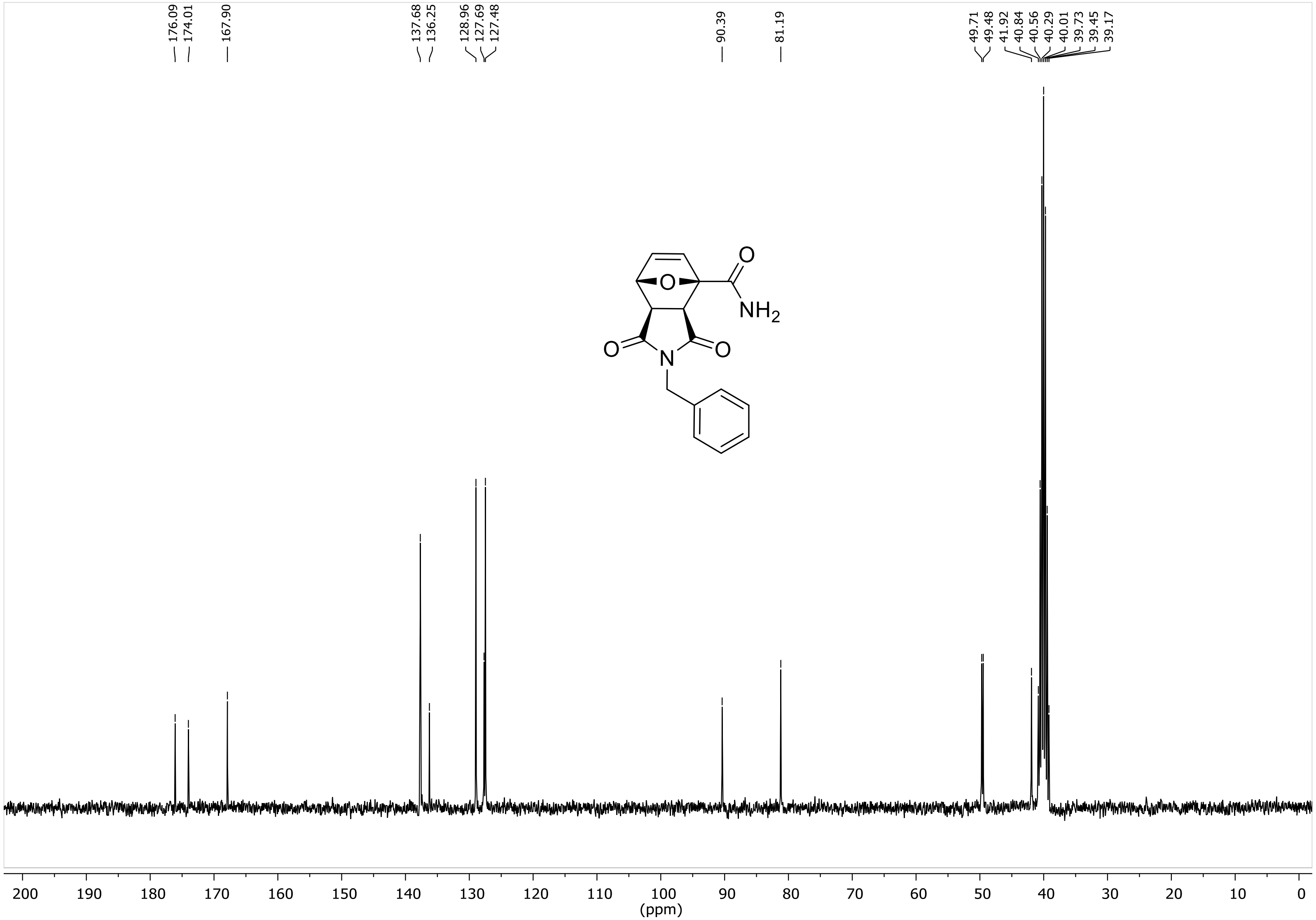

Figure S56. $^{13}C\{^{1}H\}$ NMR spectrum (75 MHz, DMSO-d_6) of *exo*-**3d**.

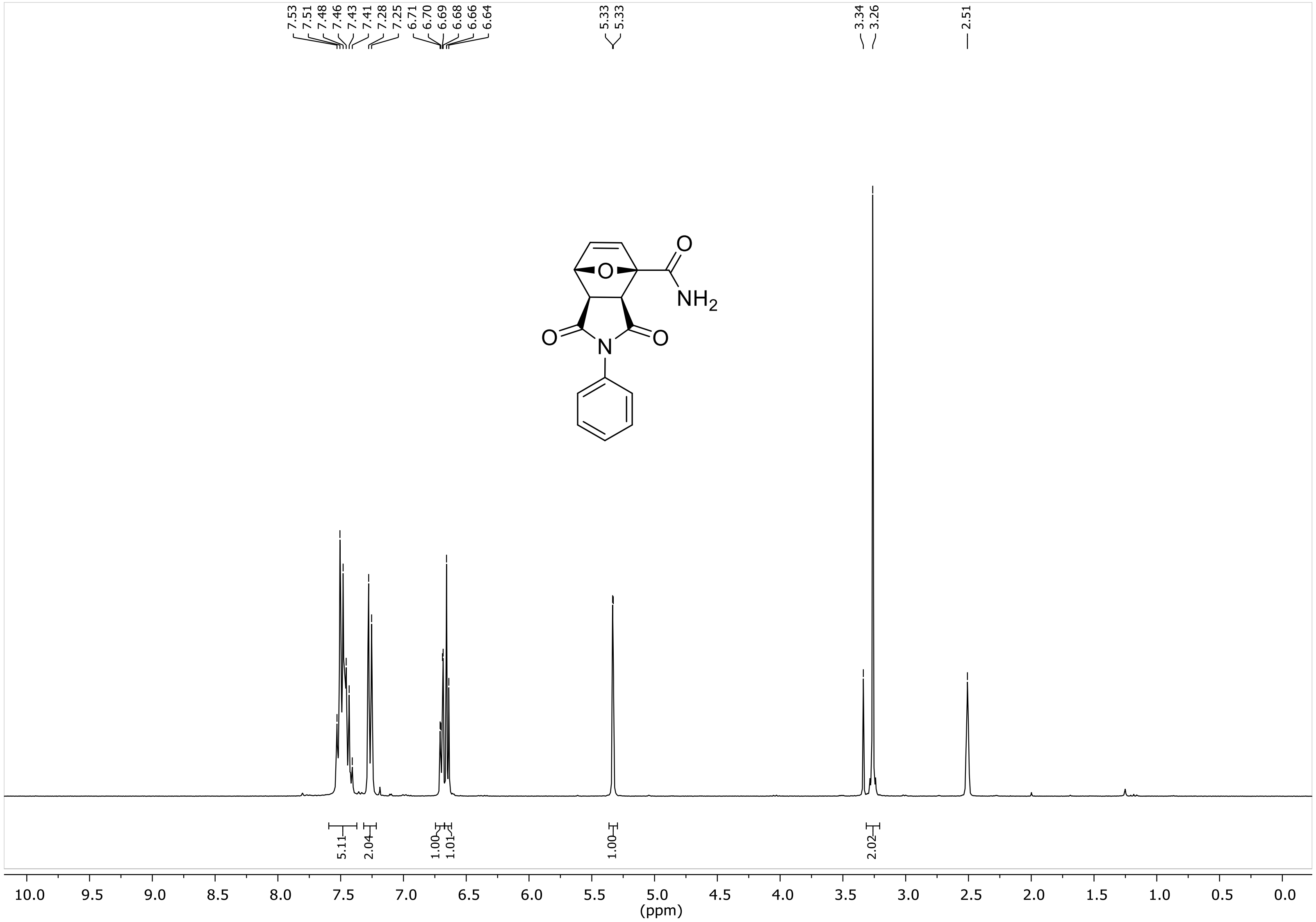

Figure S57. ^{1}H NMR spectrum (300 MHz, DMSO-d_6) of *exo*-**3e**.

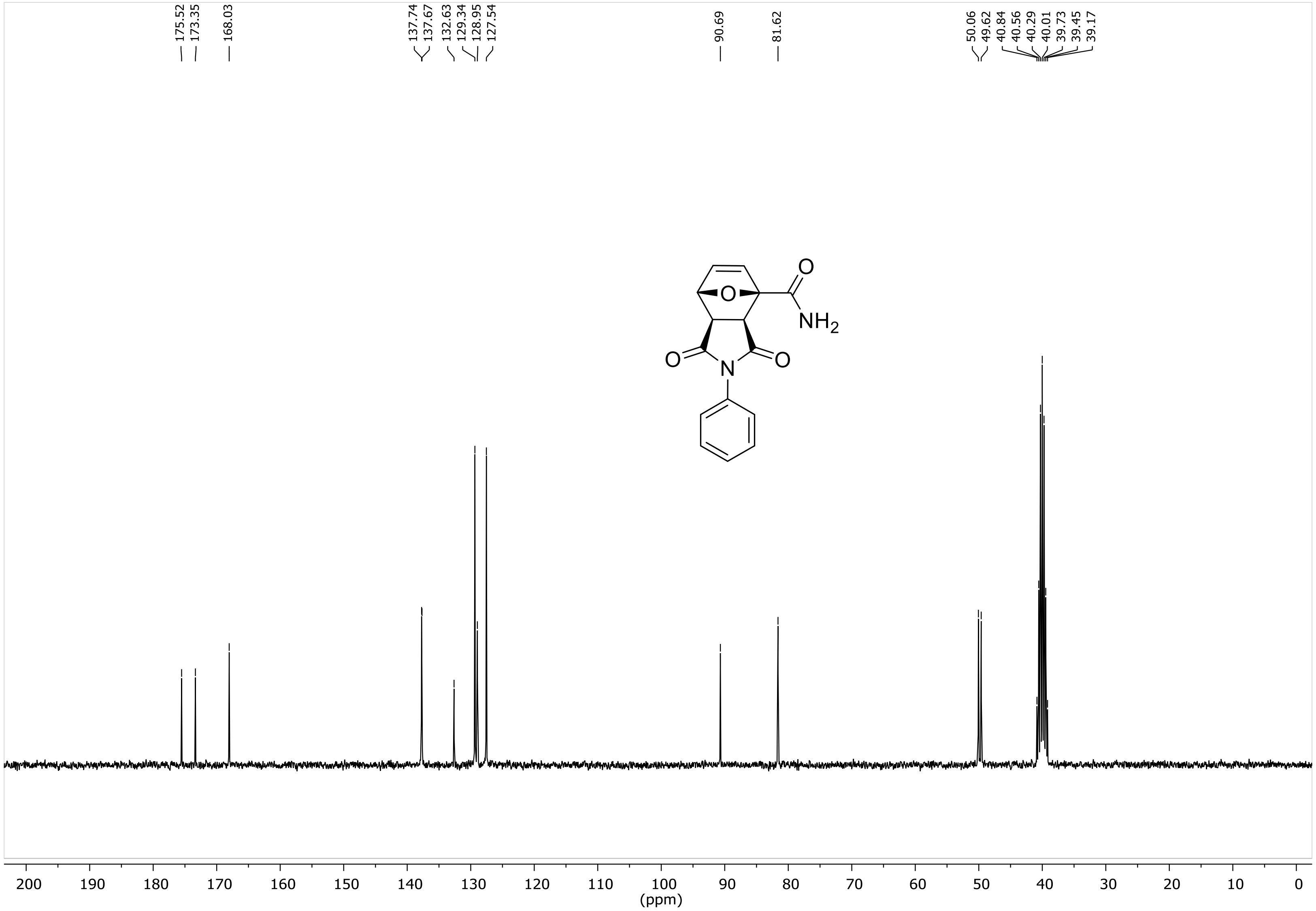

Figure S58. $^{13}C\{^{1}H\}$ NMR spectrum (75 MHz, DMSO-d_6) of *exo*-**3e**.

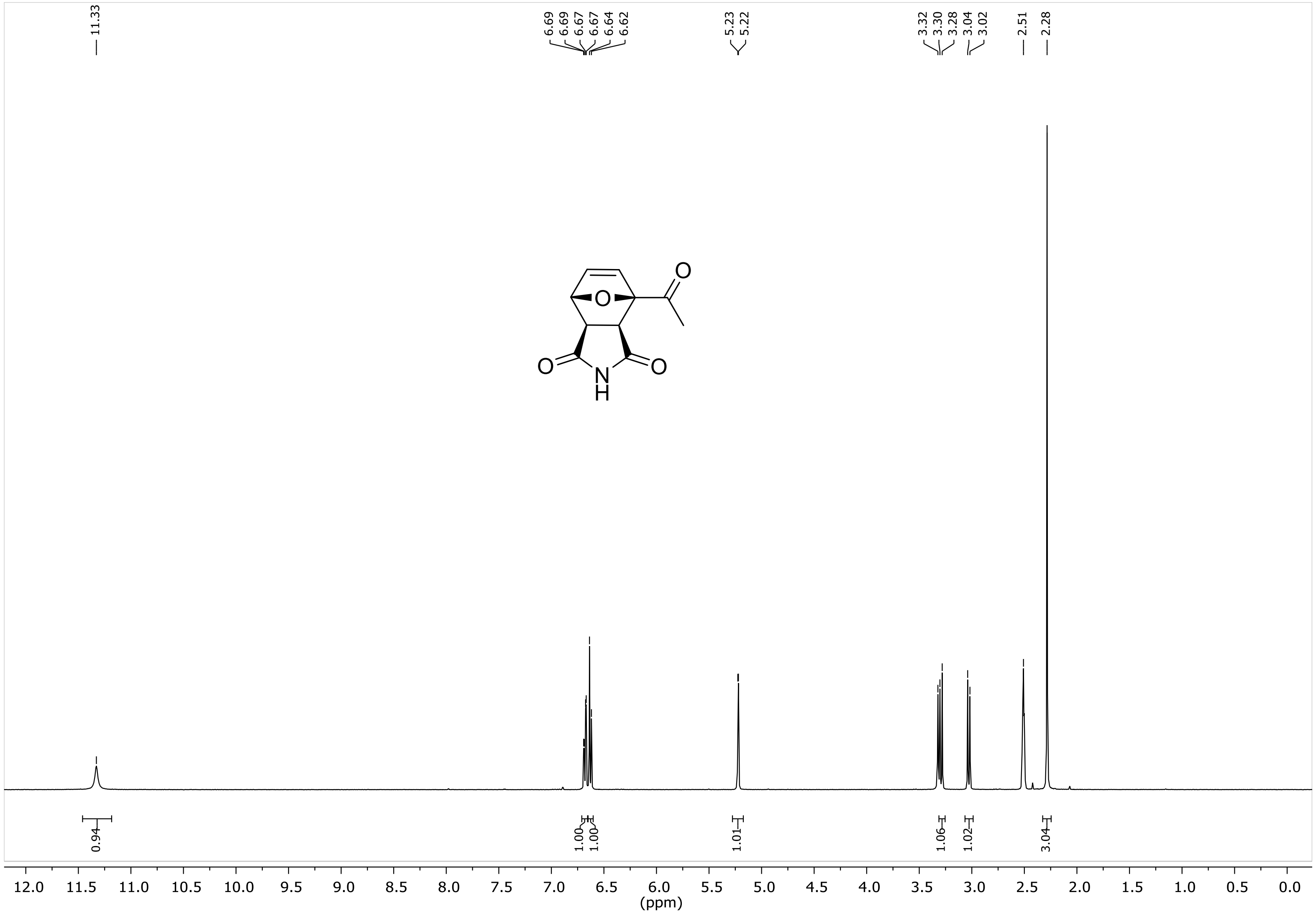

Figure S59. ^{1}H NMR spectrum (300 MHz, DMSO-d_6) of *exo*-**4a**.

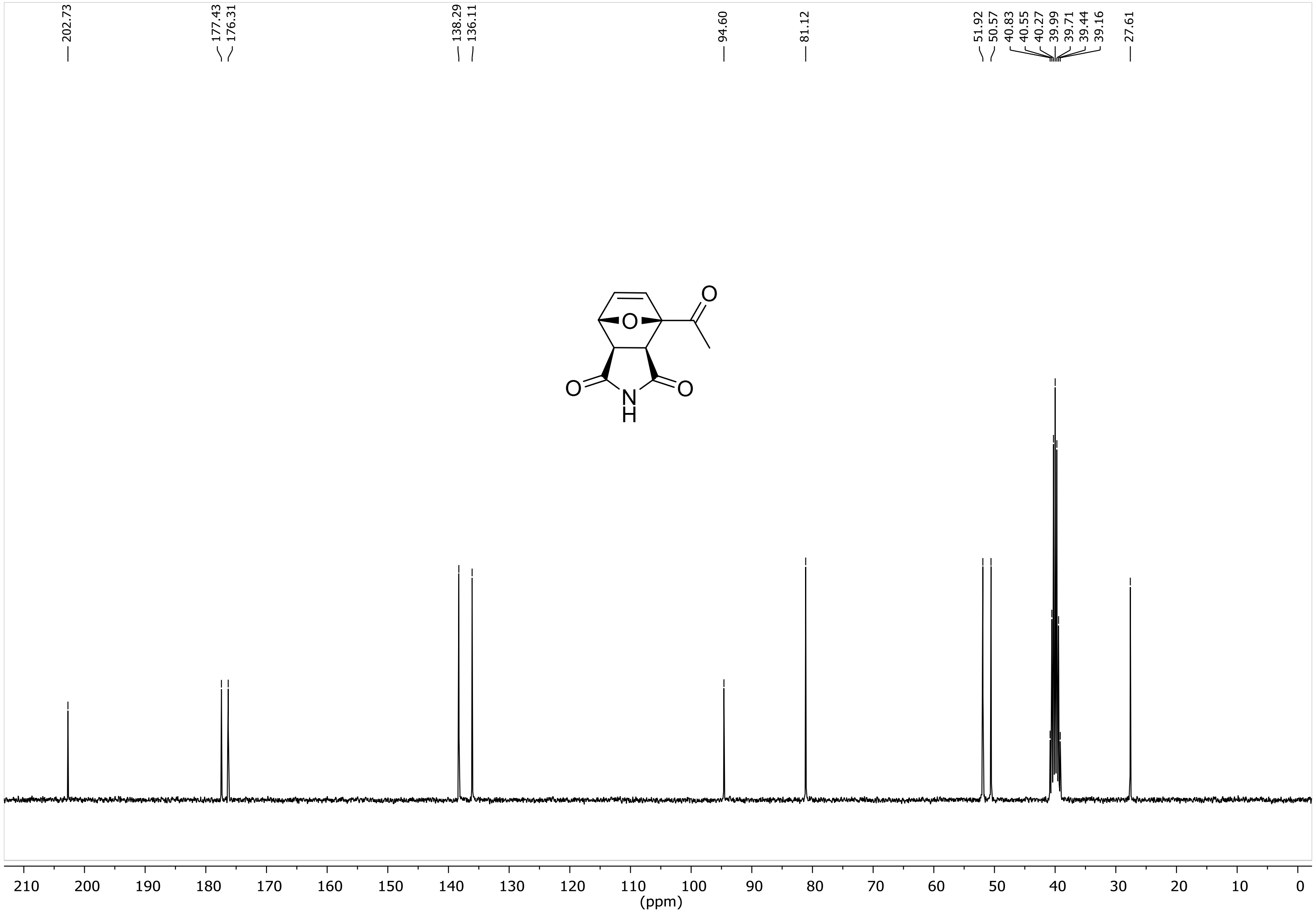

Figure S60. $^{13}C\{^{1}H\}$ NMR spectrum (75 MHz, DMSO-d_6) of *exo*-**4a**.

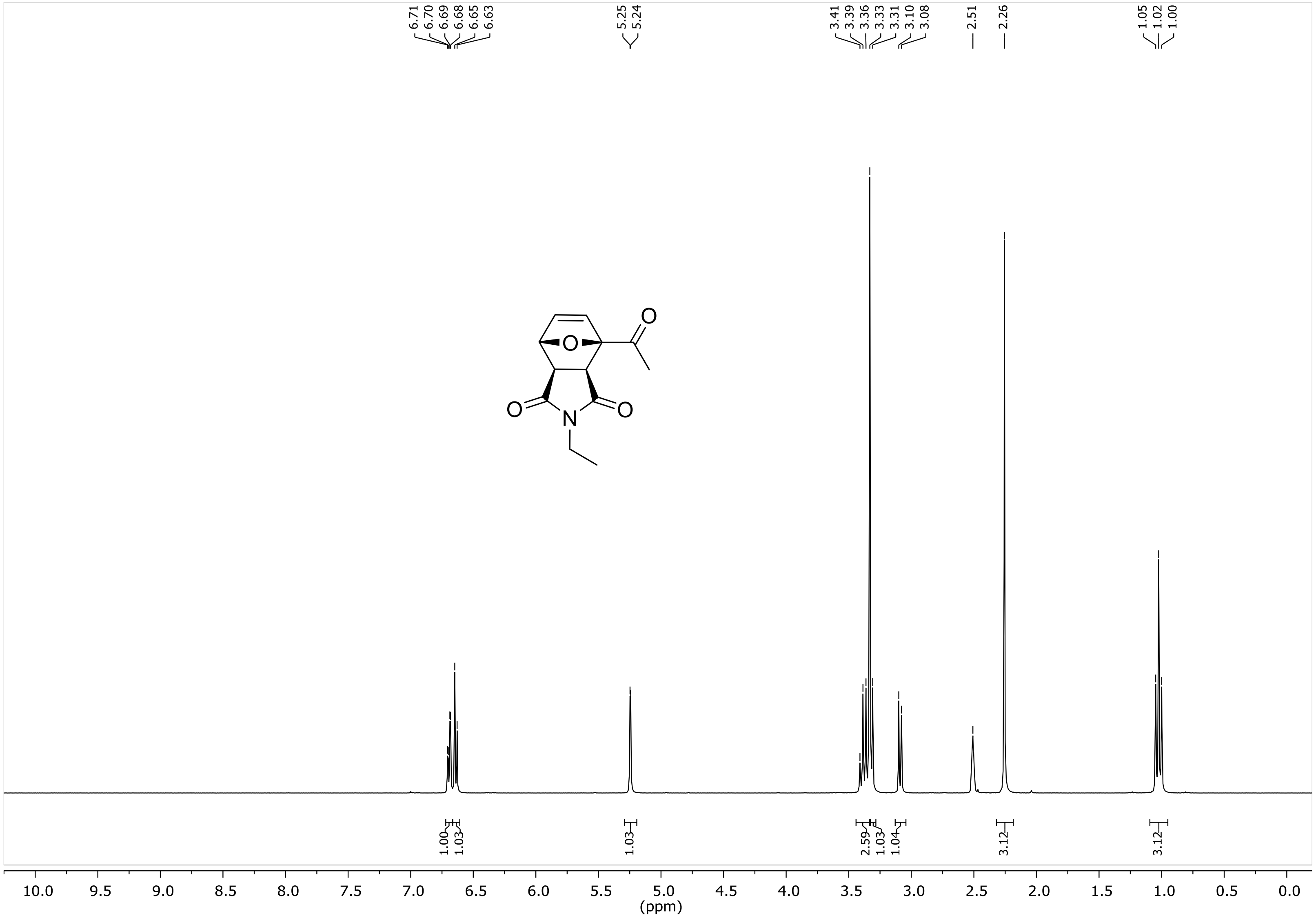

Figure S61. ^{1}H NMR spectrum (300 MHz, DMSO-d_6) of *exo*-**4b**.

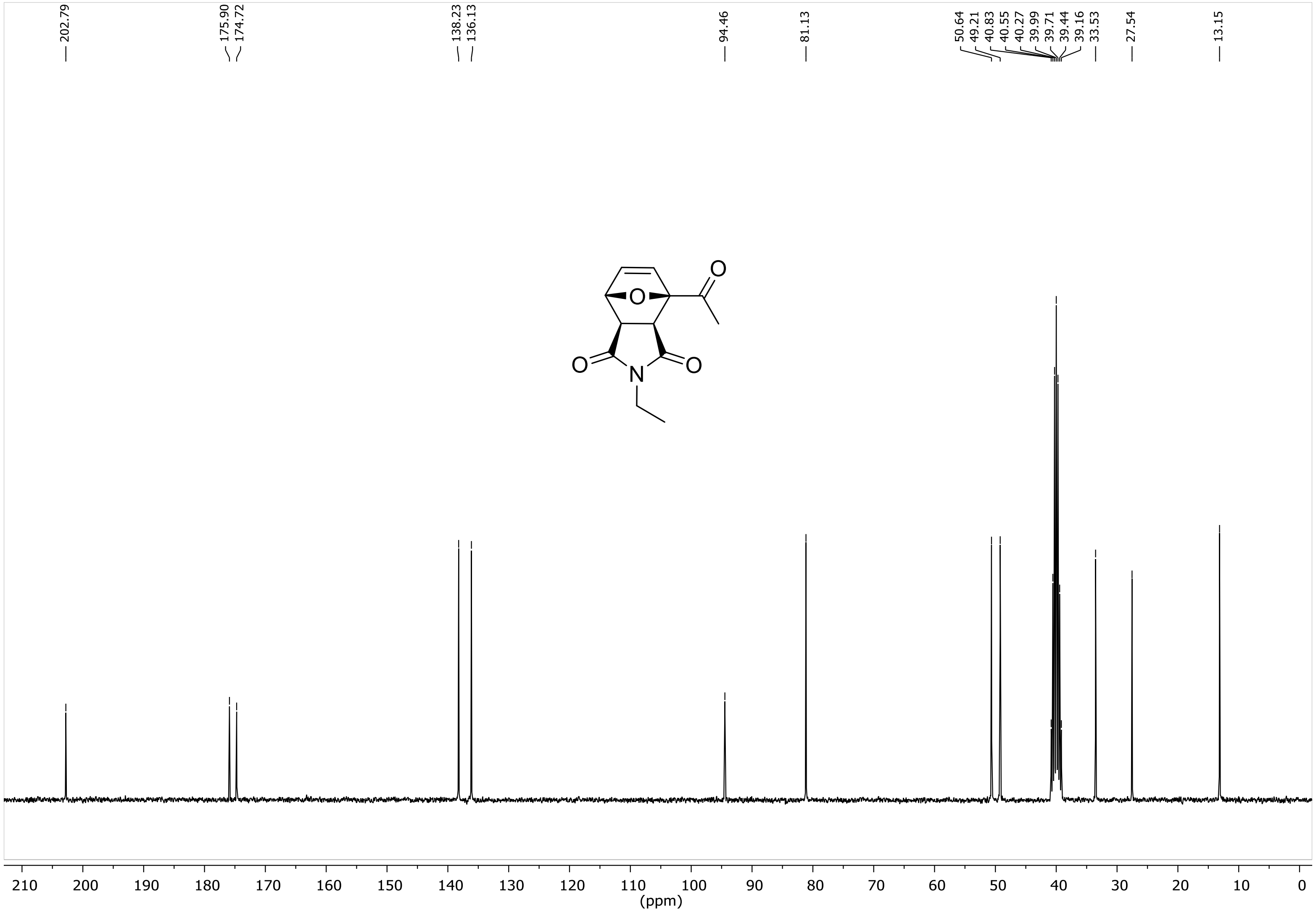

Figure S62. $^{13}C\{^{1}H\}$ NMR spectrum (75 MHz, DMSO-d_6) of *exo*-**4b**.

5.4. Solid-state NMR spectra

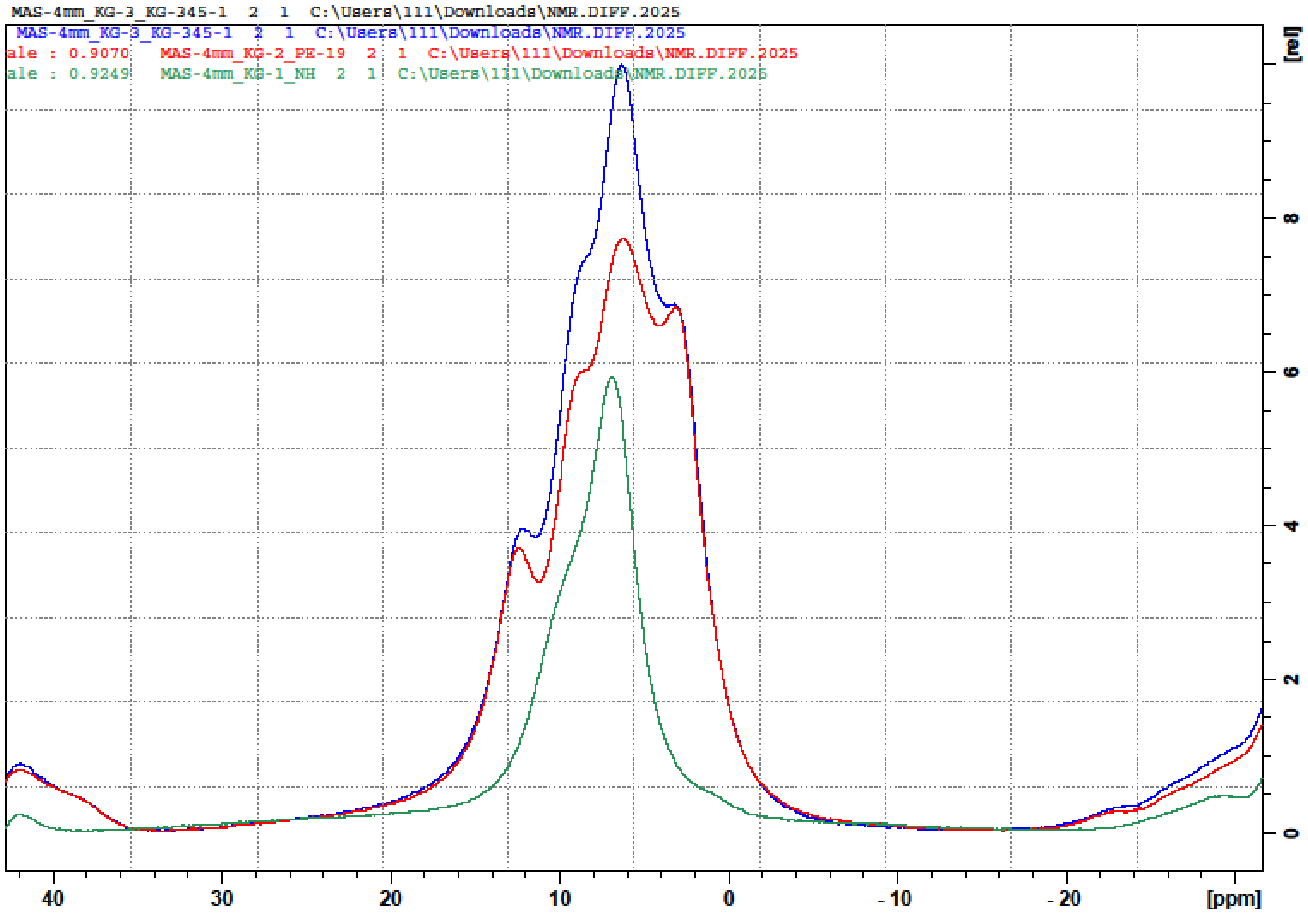

Figure S63. Comparative ^{1}H solid-state NMR (ssNMR) spectra (400.1 MHz) of maleimide (green), pure *exo*-**3a** (red), and the reaction mixture (blue) obtained from the reaction of 2-furoic acid with 1.25 equivalents of maleimide (conditions from Table S3, entry 2).

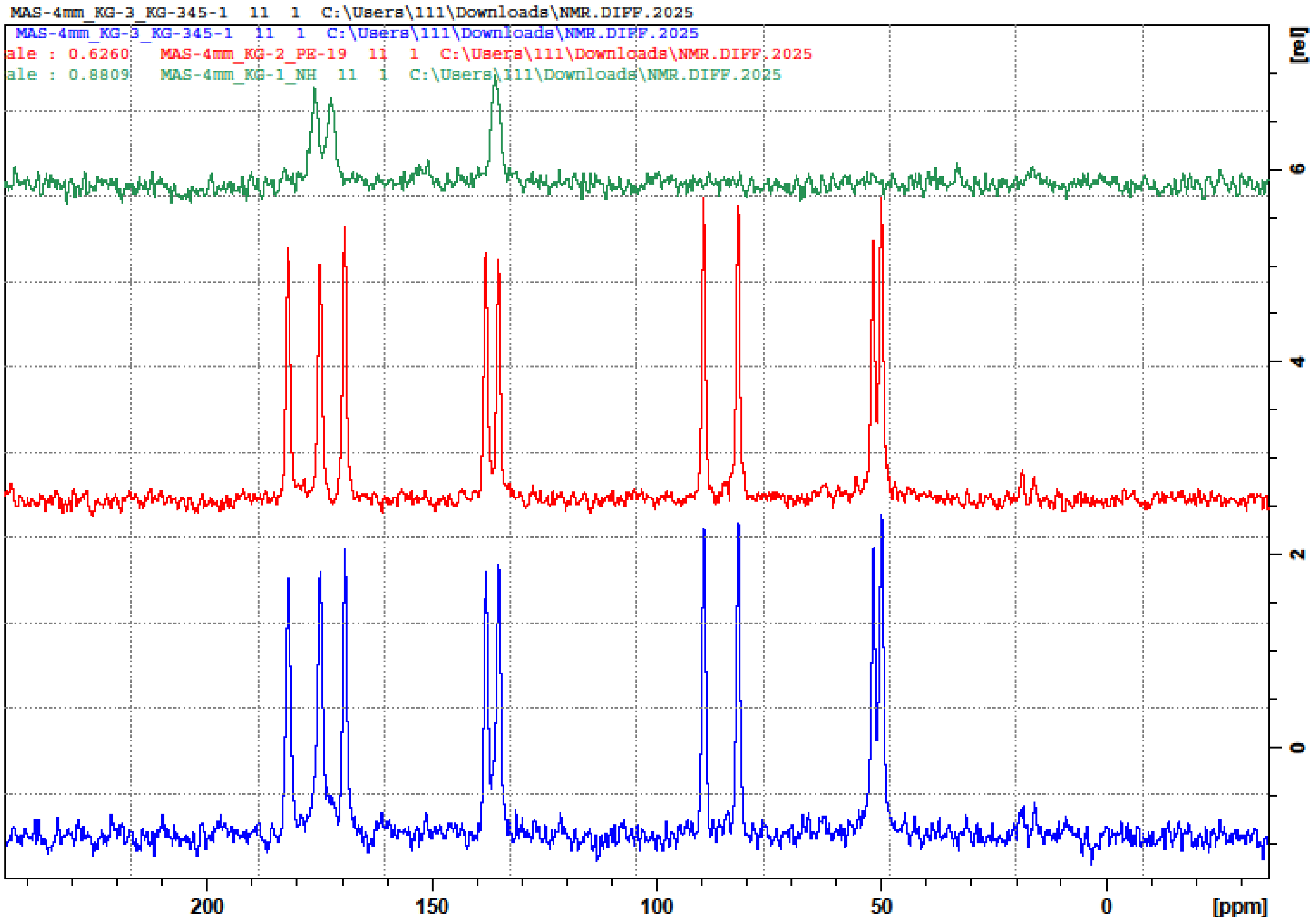

Figure S64. Comparative ^{13}C ssNMR spectra (100.6 MHz) of maleimide (green), pure *exo*-**3a** (red), and the reaction mixture (blue) obtained from the reaction of 2-furoic acid with 1.25 equivalents of maleimide (conditions from Table S3, entry 2).

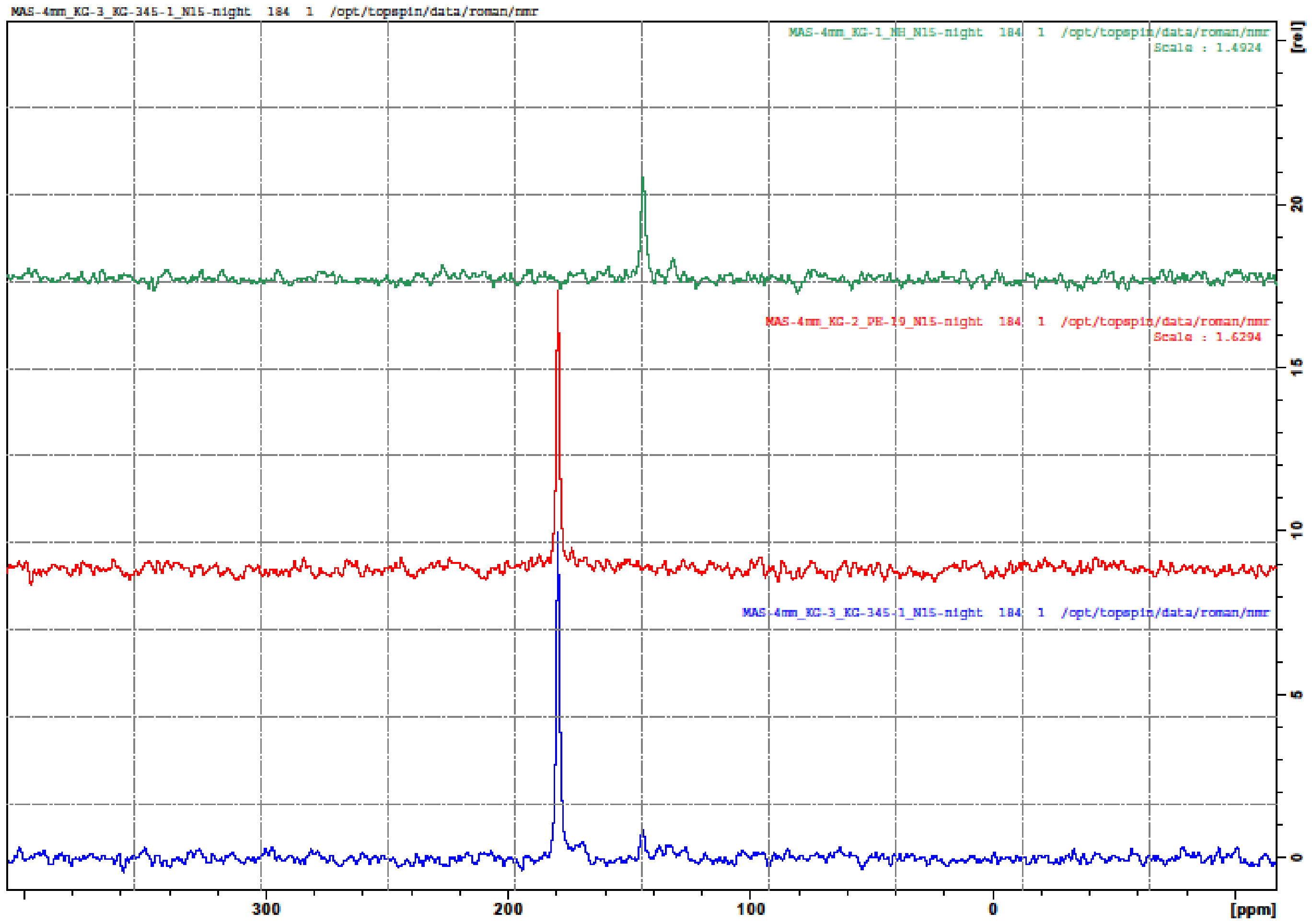

Figure S65. Comparative ^{15}N ssNMR spectra (40.6 MHz) of maleimide (green), pure *exo*-**3a** (red), and the reaction mixture (blue) obtained from the reaction of 2-furoic acid with 1.25 equivalents of maleimide (conditions from Table S3, entry 2).

6. LCMS spectra

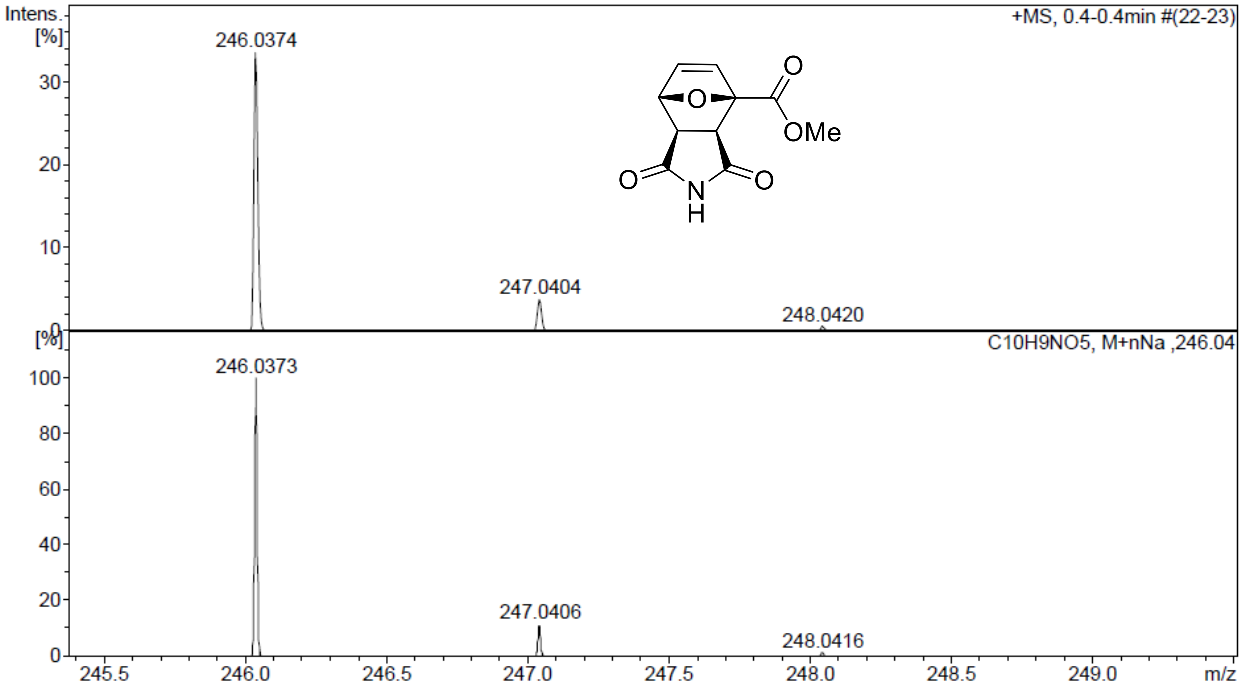

Figure S66. HRMS (ESI) spectrum of compound *exo*-**1a**.

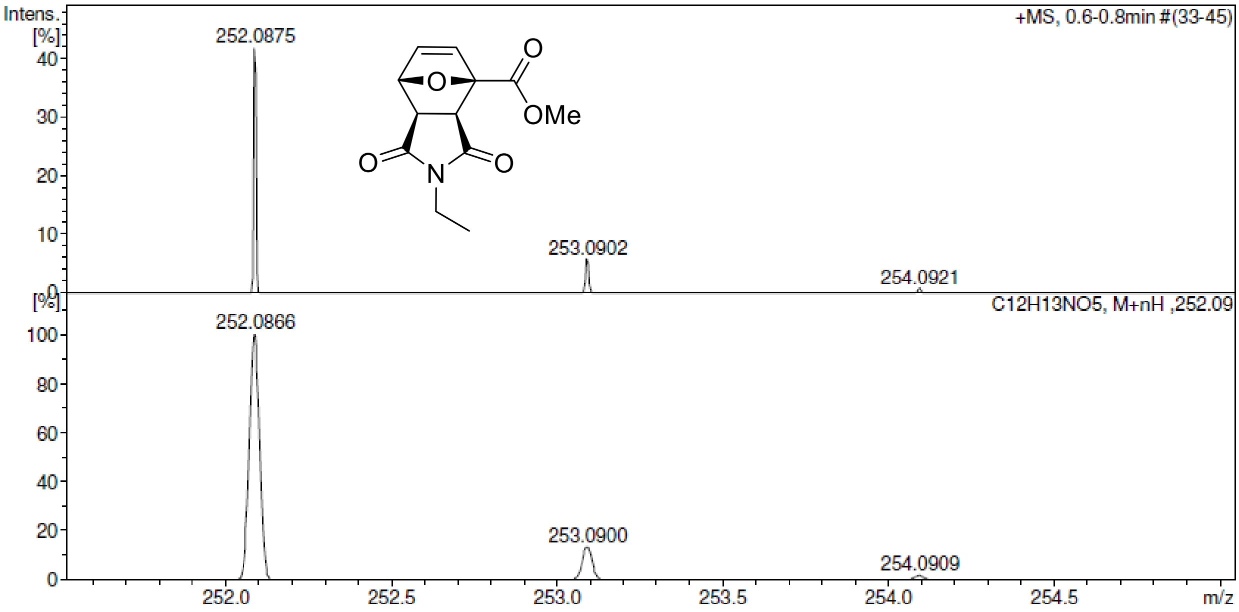

Figure S67. HRMS (ESI) spectrum of compound *exo*-**1b**.

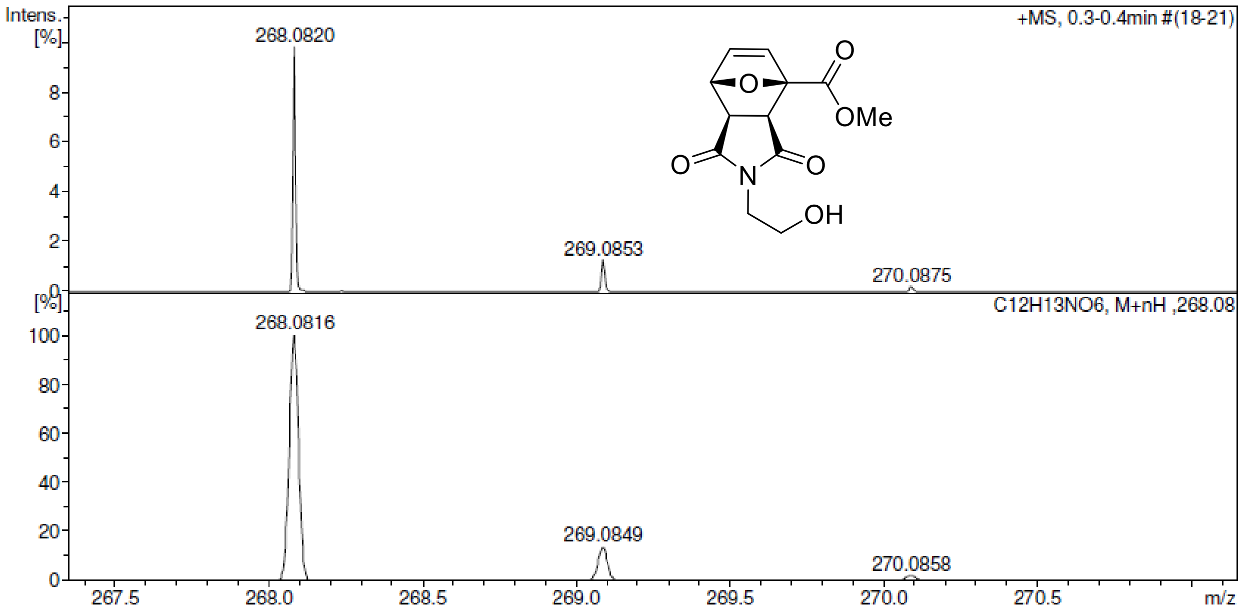

Figure S68. HRMS (ESI) spectrum of compound *exo*-**1c**.

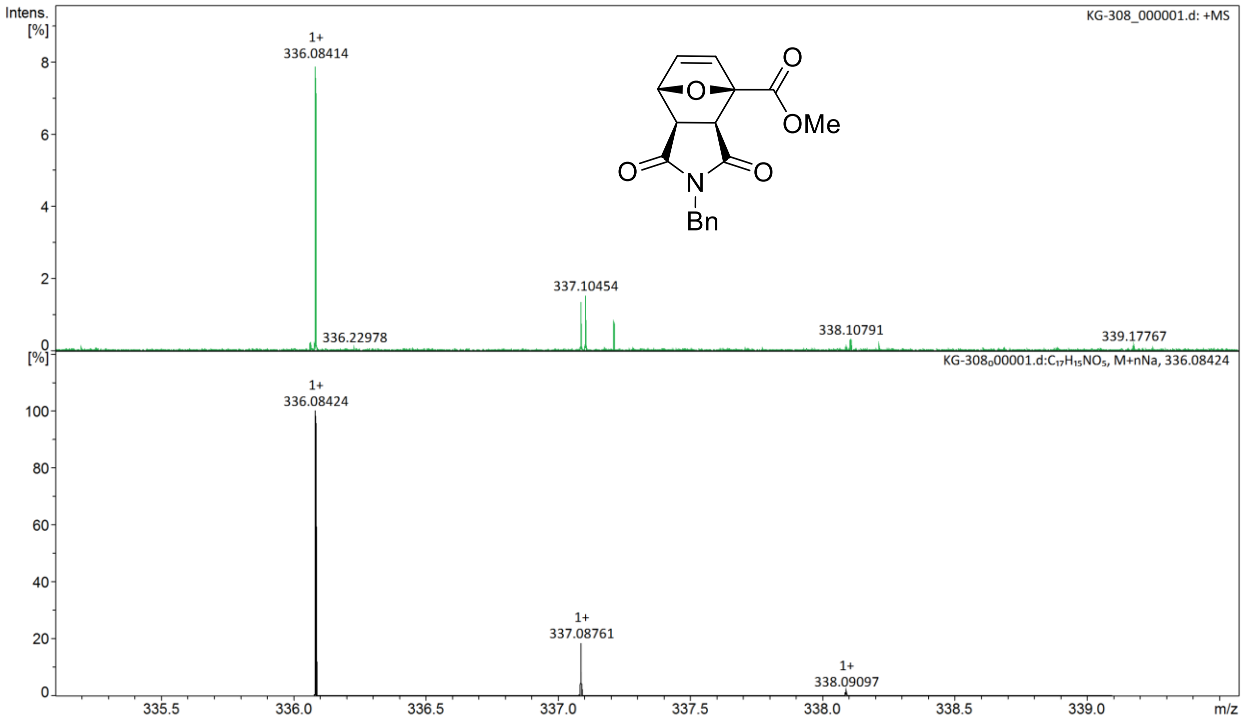

Figure S69. HRMS (ESI) spectrum of compound *exo*-**1d**.

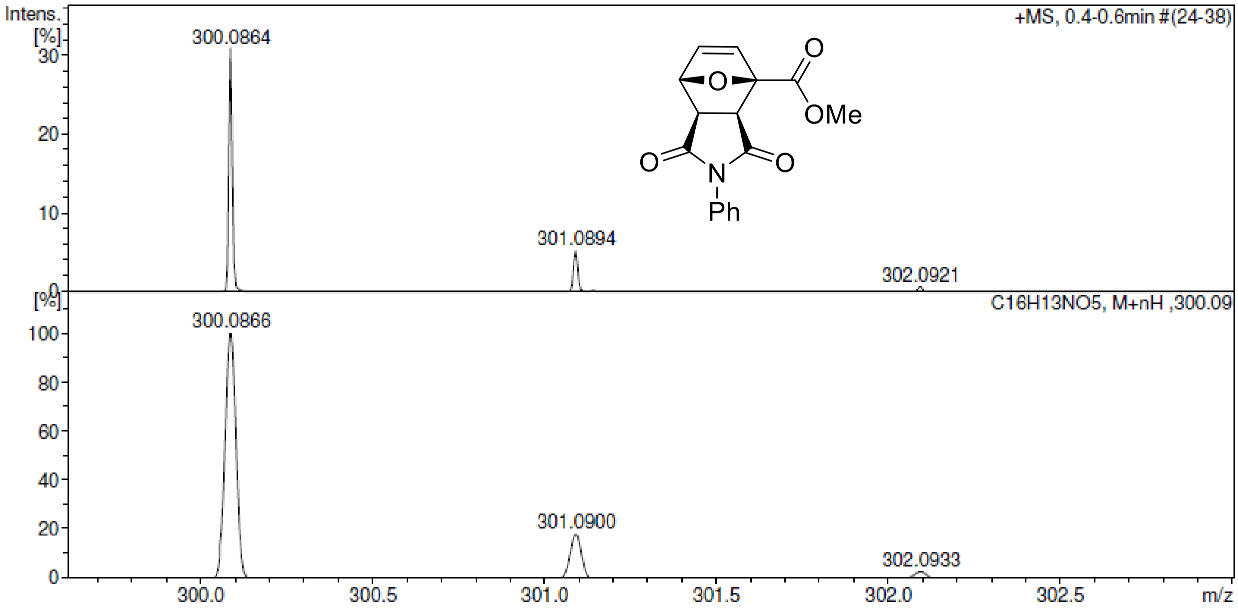

Figure S70. HRMS (ESI) spectrum of compound *exo*-**1e**.

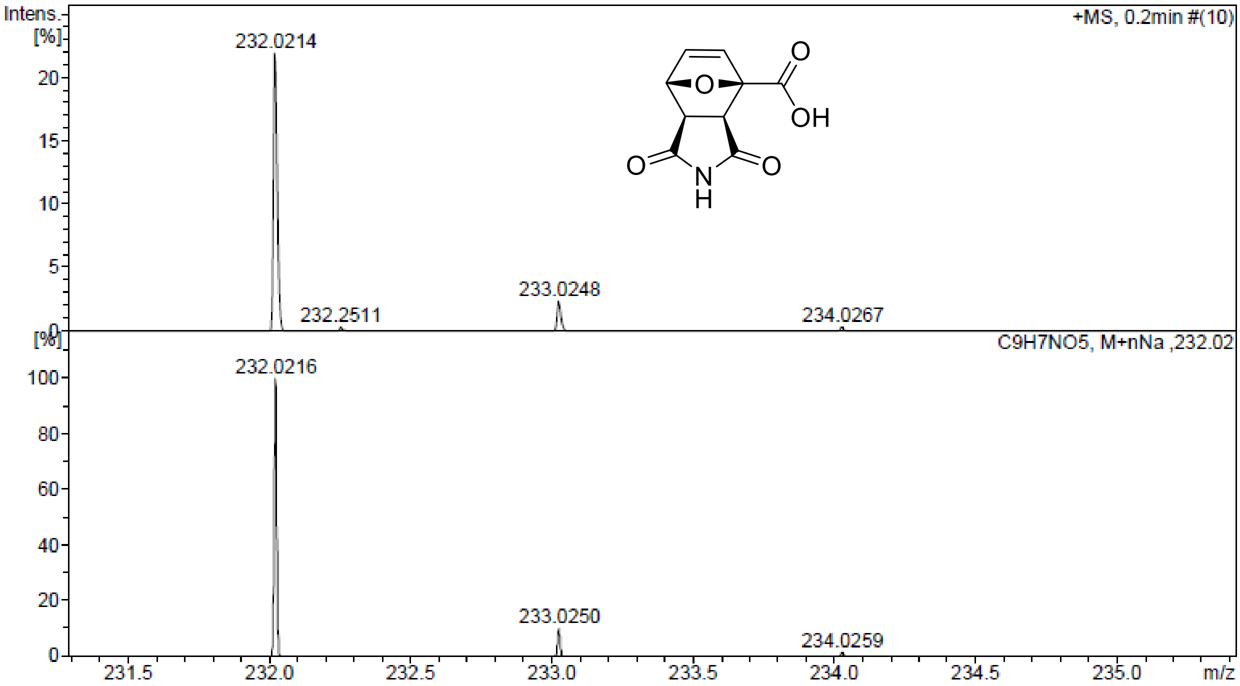

Figure S71. HRMS (ESI) spectrum of compound *exo*-**2a**.

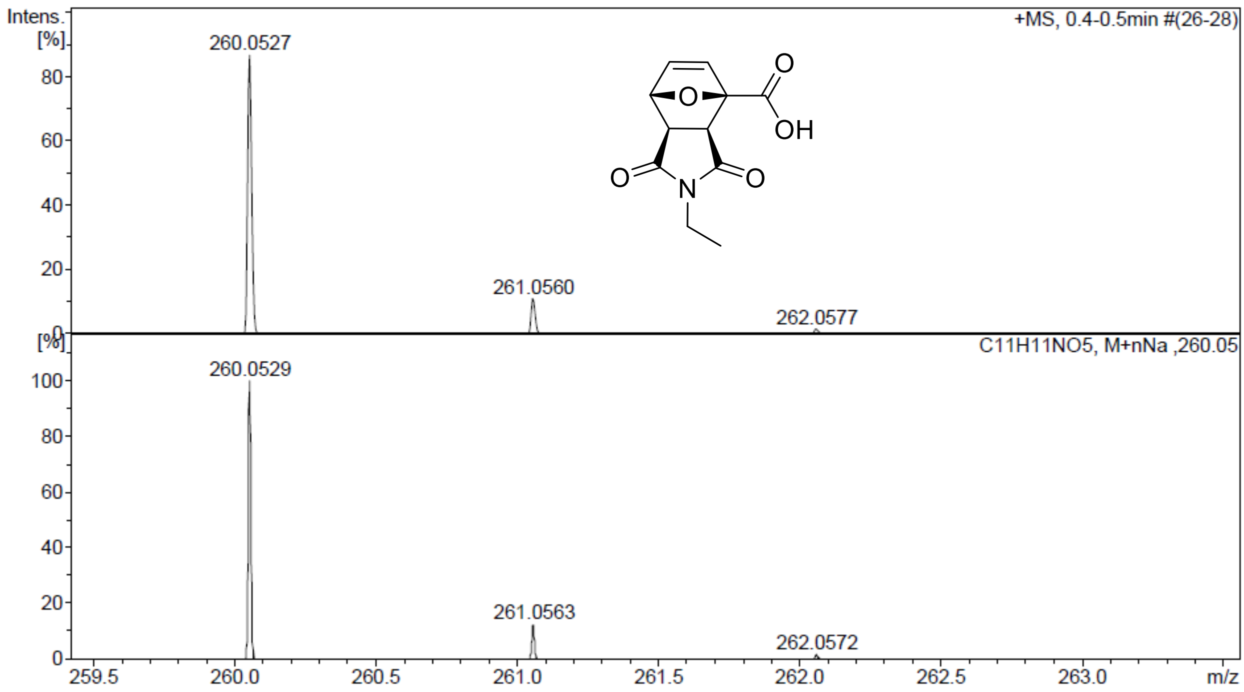

Figure S72. HRMS (ESI) spectrum of compound *exo*-**2b**.

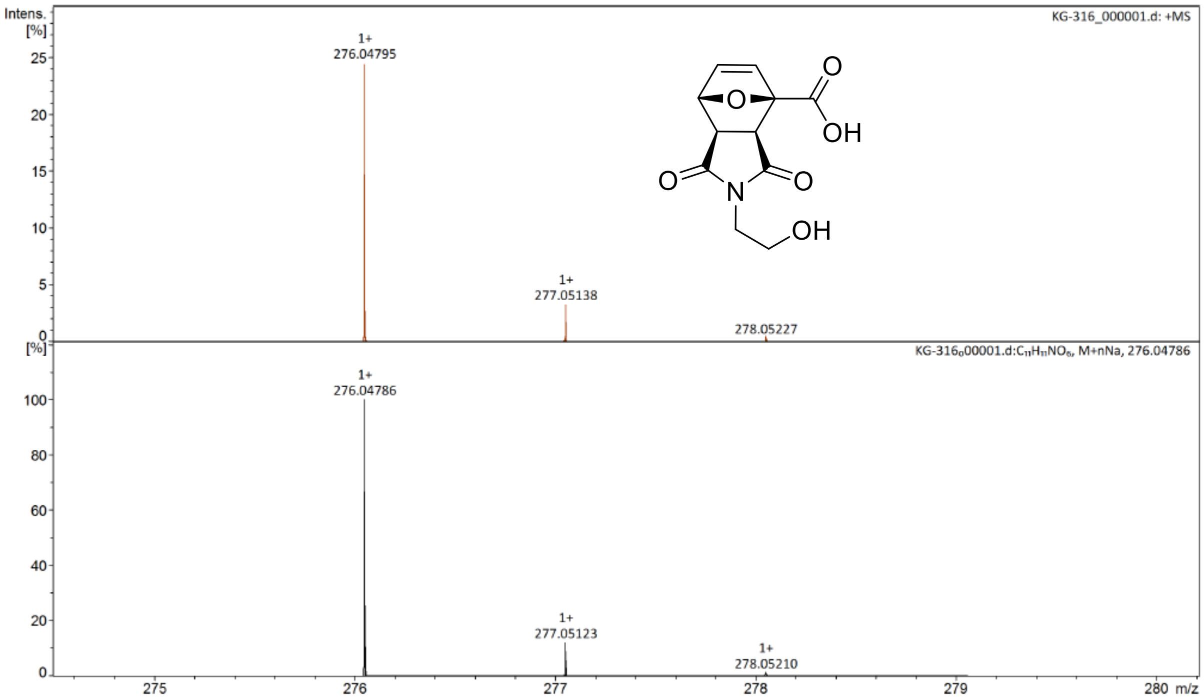

Figure S73. HRMS (ESI) spectrum of compound *exo*-**2c**.

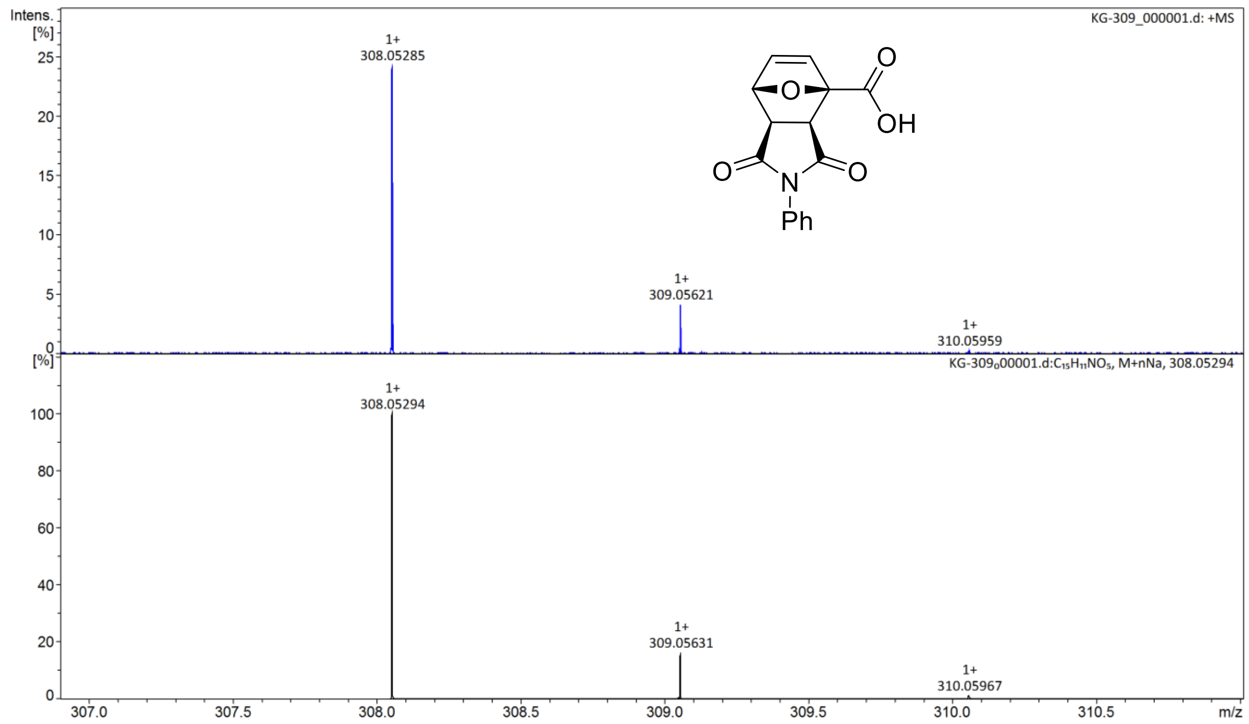

Figure S74. HRMS (ESI) spectrum of compound *exo*-**2e**.

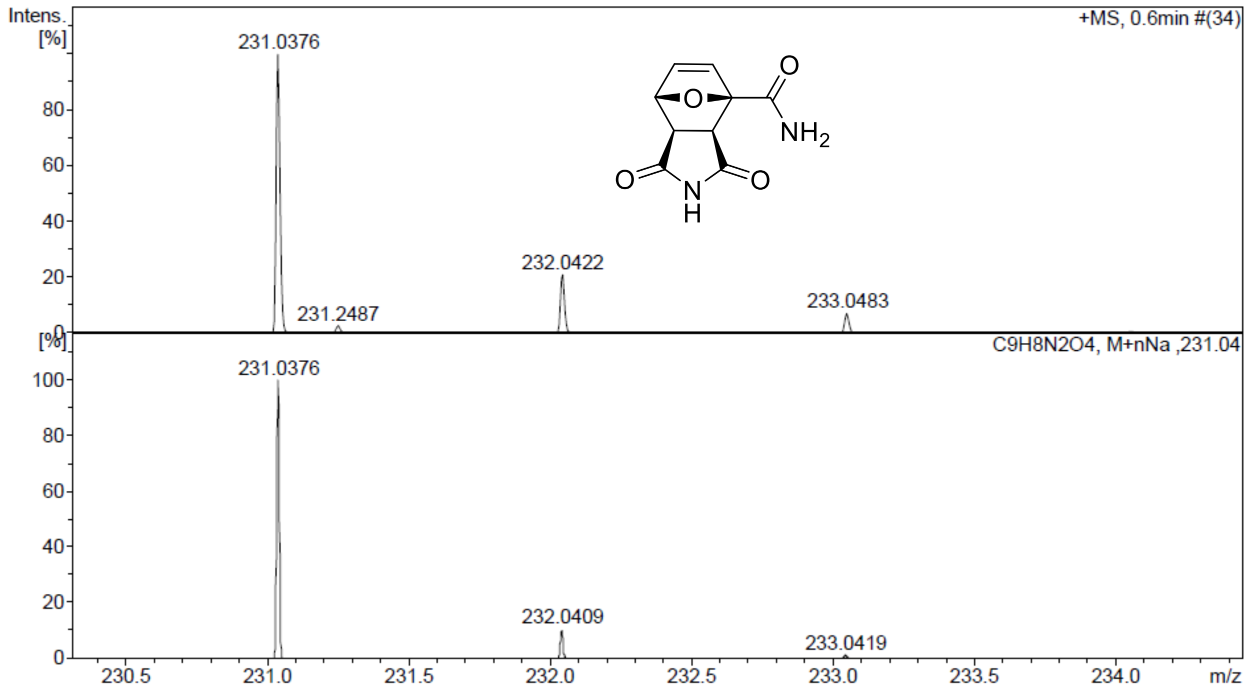

Figure S75. HRMS (ESI) spectrum of compound *exo*-**3a**.

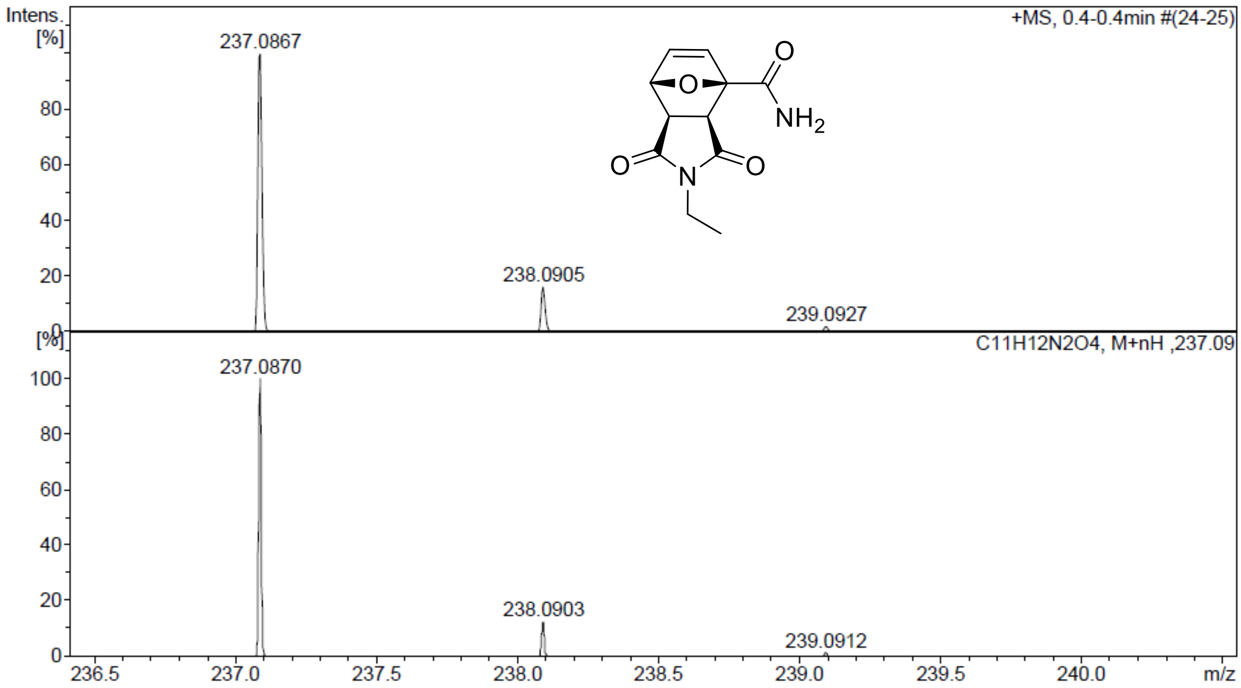

Figure S76. HRMS (ESI) spectrum of compound *exo*-**3b**.

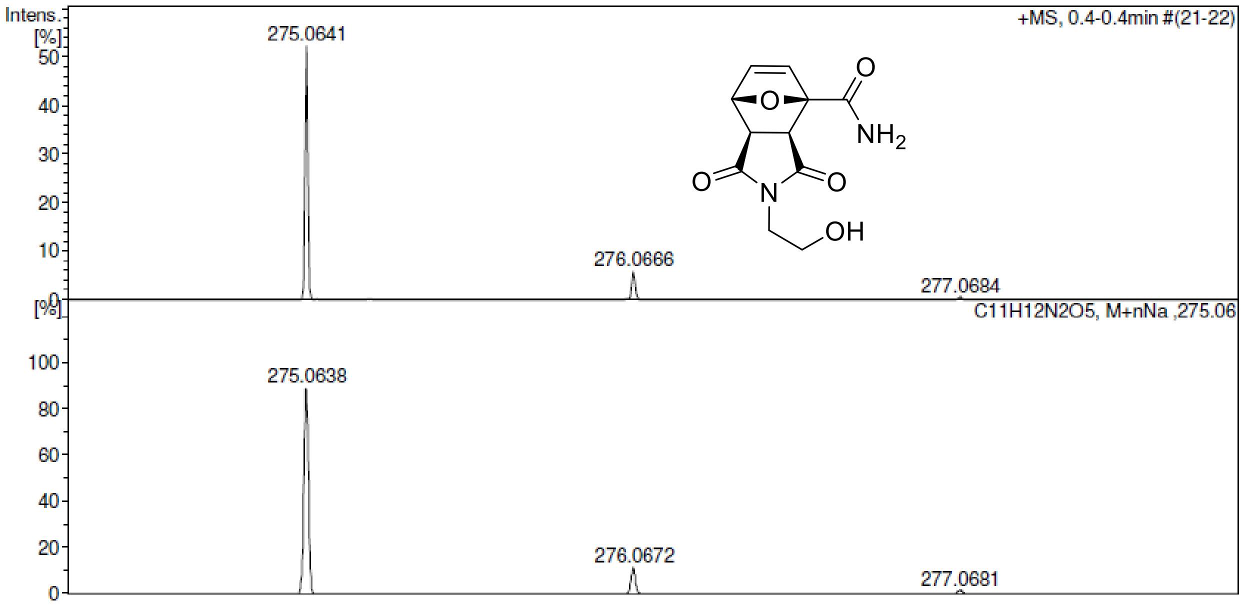

Figure S77. HRMS (ESI) spectrum of compound *exo*-**3c**.

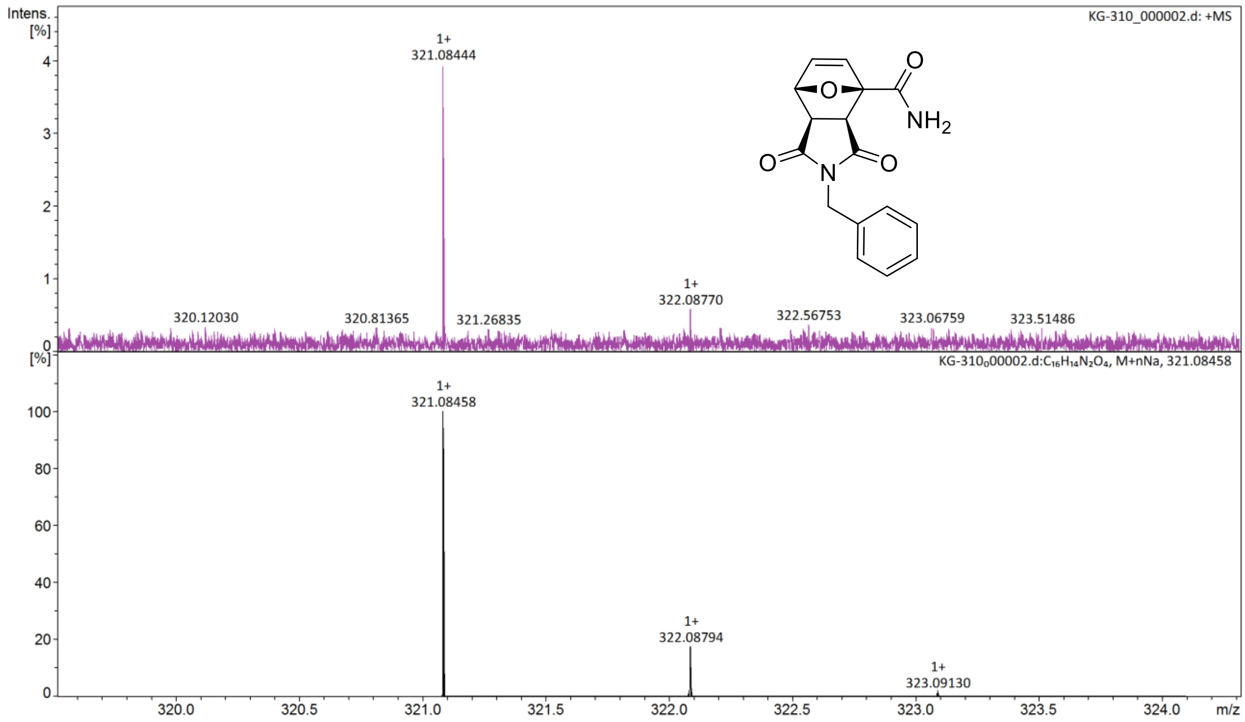

Figure S78. HRMS (ESI) spectrum of compound *exo*-**3d**.

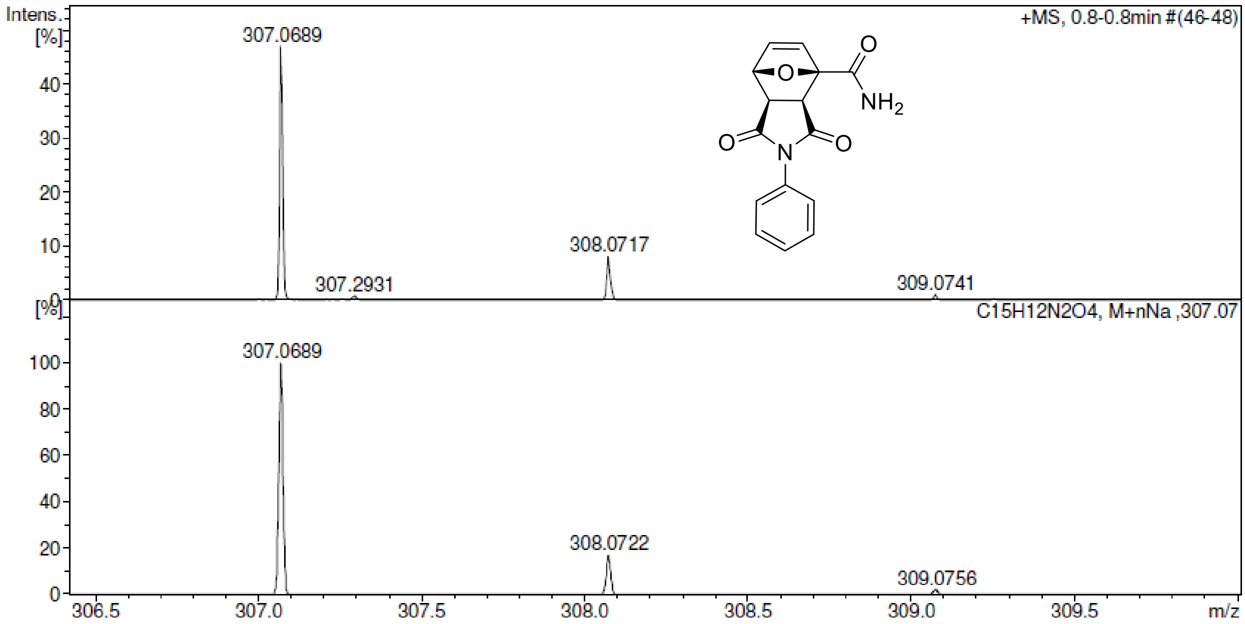

Figure S79. HRMS (ESI) spectrum of compound *exo*-**3e**.

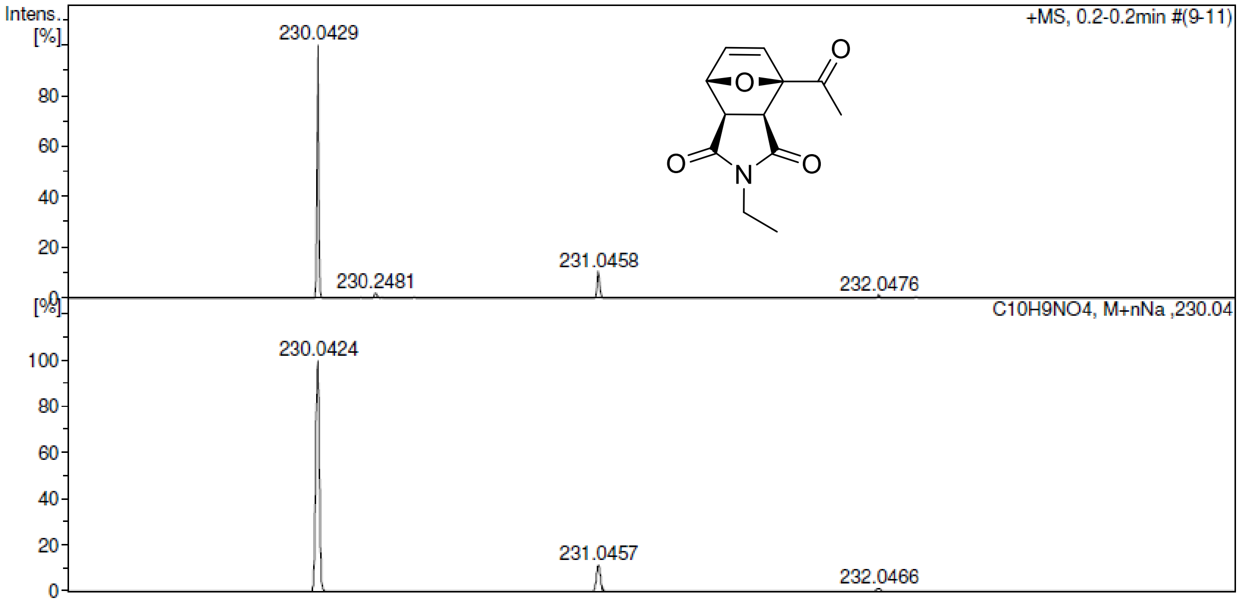

Figure S80. HRMS (ESI) spectrum of compound *exo*-**4a**.

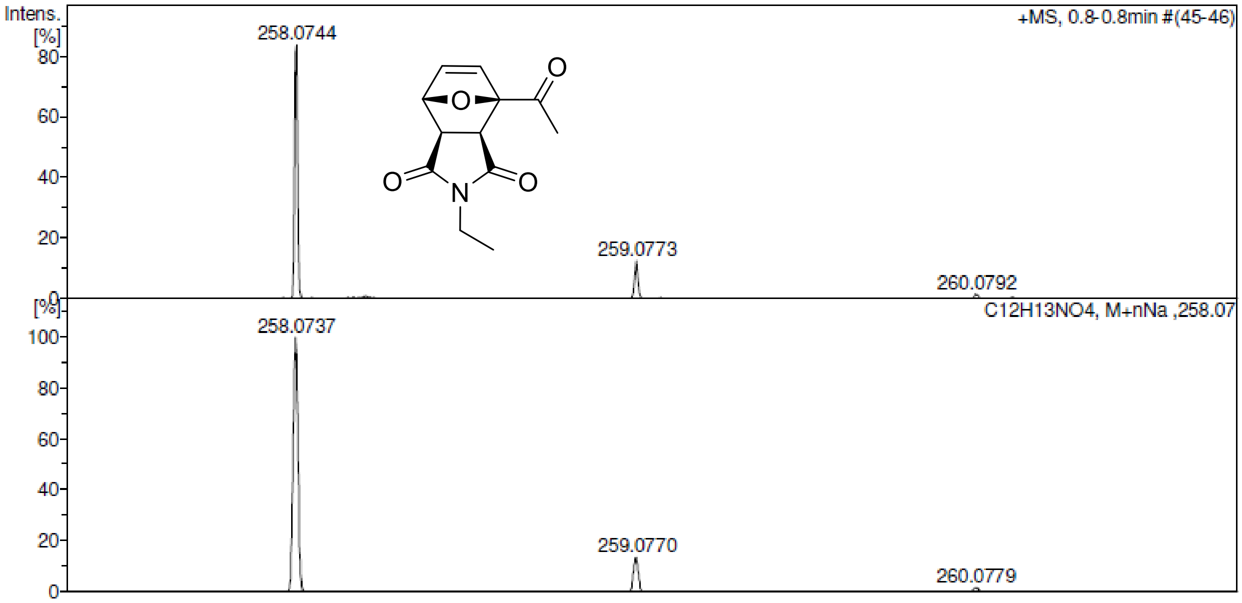

Figure S81. HRMS (ESI) spectrum of compound *exo*-**4b**.

7. Optimized xyz-Cartesian coordinates.

Methyl 2-furoate Etot = -457.853666934737

C	-0.105722000	0.054147000	-1.340612000
C	-0.105722000	-0.977249000	-0.366147000
C	-0.105722000	-0.353537000	0.846725000
O	-0.105722000	0.992225000	0.694495000
C	-0.105722000	1.236745000	-0.650930000
H	-0.105722000	-0.056141000	-2.413623000
H	-0.105722000	-2.042329000	-0.537959000
H	-0.105723000	-0.712058000	1.864719000
C	-0.105721000	2.635710000	-1.053836000
O	-0.105722000	3.579686000	-0.286105000
O	-0.105722000	2.743943000	-2.393660000
C	-0.105722000	4.095494000	-2.905446000
H	-0.998894000	4.626622000	-2.569668000
H	0.787449000	4.626623000	-2.569669000
H	-0.105723000	3.993402000	-3.988883000

2-Acetylfuran Etot = -382.626891192383

C	-0.071967000	0.051190000	-1.308744000
C	-0.143859000	-0.993857000	-0.356244000
C	-0.201644000	-0.389936000	0.867717000
O	-0.170743000	0.955026000	0.740198000
C	-0.090454000	1.228750000	-0.604559000
H	-0.013222000	-0.037965000	-2.382447000
H	-0.152108000	-2.056282000	-0.543748000

H	-0.264965000	-0.770510000	1.876016000
C	-0.042288000	2.620381000	-1.025205000
O	0.027809000	2.896469000	-2.220047000
C	-0.081471000	3.672380000	0.048056000
H	-0.996031000	3.572923000	0.640802000
H	0.758468000	3.539744000	0.737003000
H	-0.037635000	4.660905000	-0.407456000

N-Etylmaleimide Etot = -438.005185809086

C	1.938938000	0.666329000	0.259662000
C	1.938993000	-0.666135000	0.259788000
C	0.558896000	-1.138427000	-0.073602000
C	0.558774000	1.138443000	-0.073696000
O	0.163408000	-2.283486000	-0.162681000
O	0.163198000	2.283452000	-0.163026000
N	-0.212512000	-0.000042000	-0.273426000
H	2.750661000	1.352735000	0.455452000
H	2.750774000	-1.352438000	0.455697000
C	-1.637993000	-0.000123000	-0.578963000
H	-1.837949000	-0.885107000	-1.188688000
H	-1.838018000	0.884727000	-1.188861000
C	-2.496317000	-0.000037000	0.679593000
H	-2.296575000	0.888253000	1.284373000
H	-3.553945000	-0.000099000	0.404193000
H	-2.296512000	-0.888203000	1.284534000

N-Phenylmaleimide Etot = -590.404111356660

C	2.977724000	0.628019000	-0.221757000
C	2.977681000	-0.628400000	0.221025000
C	1.564955000	-1.079735000	0.385608000
C	1.565019000	1.080399000	-0.383671000
O	1.168166000	-2.153300000	0.781598000
O	1.168291000	2.153495000	-0.780991000
N	0.757143000	0.000236000	0.000641000
C	-0.662413000	0.000146000	0.000296000
C	-1.350880000	0.993520000	0.693305000
C	-1.350452000	-0.993334000	-0.692991000
C	-2.741329000	0.993554000	0.682251000
H	-0.801164000	1.753488000	1.236573000
C	-2.740906000	-0.993585000	-0.682494000
H	-0.800401000	-1.753226000	-1.236026000
C	-3.438110000	-0.000065000	-0.000266000
H	-3.279794000	1.767991000	1.217414000
H	-3.279038000	-1.768106000	-1.217870000
H	-4.522616000	-0.000144000	-0.000488000
H	3.813167000	1.273594000	-0.453099000
H	3.813084000	-1.274612000	0.450726000

Exo-**1b** Etot = -895.876355372146

C	-0.223424000	0.062276000	-2.468337000
C	0.461734000	0.765301000	-1.317024000
C	-0.776878000	-0.731383000	-0.417457000
C	-1.001141000	-0.866058000	-1.913883000

H	-0.156864000	0.361086000	-3.505350000
H	-1.732869000	-1.521630000	-2.363981000
O	0.582514000	-0.289581000	-0.337581000
C	-0.636848000	1.656016000	-0.640286000
H	-1.118059000	2.334809000	-1.344447000
C	-1.542346000	0.594220000	0.000139000
H	-2.584781000	0.579532000	-0.315557000
H	1.418238000	1.253354000	-1.491015000
C	-1.434302000	0.836722000	1.490983000
O	-1.990485000	0.208755000	2.370079000
C	-0.058102000	2.425086000	0.528658000
O	0.733401000	3.343489000	0.473864000
N	-0.565005000	1.891809000	1.704626000
C	-0.148361000	2.329774000	3.035008000
H	0.083386000	3.395005000	2.965738000
H	-1.005064000	2.201706000	3.700767000
C	1.054451000	1.535951000	3.529160000
H	1.907315000	1.668288000	2.858634000
H	0.816810000	0.470758000	3.589125000
H	1.339299000	1.883897000	4.524894000
C	-1.114604000	-1.927149000	0.433633000
O	-2.160752000	-2.523926000	0.304744000
O	-0.165357000	-2.209566000	1.318774000
C	-0.457674000	-3.311611000	2.216489000
H	-1.352998000	-3.085240000	2.798358000
H	-0.604915000	-4.229182000	1.644387000
H	0.413687000	-3.393822000	2.862298000

TS-*exo*-**1b** Etot = -895.843625342632 i445 cm-1

C	-0.066280000	0.153098000	-2.458496000
C	0.572202000	0.785580000	-1.347898000
C	-0.464008000	-0.924968000	-0.554600000
C	-0.749918000	-0.913077000	-1.944181000
H	-0.111991000	0.556275000	-3.459051000
H	-1.453879000	-1.566368000	-2.436410000
O	0.610434000	-0.123095000	-0.310192000
C	-0.971200000	1.818123000	-0.614100000
H	-1.207098000	2.438494000	-1.469086000
C	-1.829726000	0.829982000	-0.088521000
H	-2.727342000	0.445184000	-0.549585000
H	1.402364000	1.480335000	-1.374841000
C	-1.693352000	0.832949000	1.377320000
O	-2.290125000	0.161054000	2.202795000
C	-0.304082000	2.469511000	0.561988000
O	0.472036000	3.407119000	0.555184000
N	-0.722131000	1.789947000	1.695168000
C	-0.193316000	2.018149000	3.032848000
H	0.037466000	3.083683000	3.113996000
H	-0.994090000	1.786676000	3.739850000
C	1.042247000	1.171669000	3.314289000
H	1.838228000	1.399285000	2.600242000
H	0.805313000	0.107237000	3.244501000
H	1.411349000	1.380536000	4.321638000
C	-0.835159000	-1.961665000	0.423117000
O	-1.758072000	-2.727340000	0.234626000
O	-0.066819000	-1.924558000	1.511378000

C	-0.422070000	-2.863213000	2.556443000
H	-1.444872000	-2.676979000	2.888329000
H	-0.328060000	-3.886110000	2.187324000
H	0.285860000	-2.677007000	3.361264000

Exo-**1e** Etot = -1048.273740599278

C	-0.237456000	-0.010110000	-2.533253000
C	0.471264000	0.759328000	-1.440578000
C	-0.727287000	-0.697316000	-0.428987000
C	-0.990243000	-0.914243000	-1.908795000
H	-0.201094000	0.233022000	-3.586135000
H	-1.726919000	-1.599822000	-2.302465000
O	0.628079000	-0.238810000	-0.408970000
C	-0.619037000	1.676052000	-0.782392000
H	-1.124699000	2.313017000	-1.508173000
C	-1.496812000	0.644056000	-0.063852000
H	-2.546362000	0.601603000	-0.351827000
H	1.417225000	1.246491000	-1.666465000
C	-1.363124000	0.962172000	1.408314000
O	-1.912709000	0.390807000	2.324540000
C	-0.012128000	2.513156000	0.321348000
O	0.767664000	3.429144000	0.191264000
N	-0.475994000	2.029472000	1.548938000
C	-0.056924000	2.544864000	2.810420000
C	0.585157000	1.699553000	3.710599000
C	-0.291580000	3.881290000	3.118209000
C	0.992461000	2.203397000	4.940650000

C	0.128703000	4.376744000	4.347753000
C	0.767310000	3.540203000	5.259295000
H	0.764690000	0.663676000	3.444880000
H	-0.798963000	4.519760000	2.404137000
H	1.491751000	1.550477000	5.647906000
H	-0.048889000	5.417659000	4.594293000
H	1.090005000	3.930159000	6.218416000
C	-1.032189000	-1.845774000	0.497012000
O	-2.069735000	-2.465657000	0.420512000
O	-0.066748000	-2.055871000	1.384482000
C	-0.327528000	-3.103342000	2.354718000
H	-1.217399000	-2.853845000	2.935452000
H	-0.468581000	-4.057330000	1.844008000
H	0.554875000	-3.131019000	2.990027000

TS-*exo*-**1e** Etot = -1048.242708777951 i434 cm-1

C	-0.076838000	0.185849000	-2.441887000
C	0.531529000	0.874420000	-1.345076000
C	-0.397554000	-0.882521000	-0.519238000
C	-0.698017000	-0.906781000	-1.904677000
H	-0.152008000	0.569069000	-3.448467000
H	-1.368417000	-1.605982000	-2.380437000
O	0.626131000	-0.018864000	-0.292604000
C	-1.005763000	1.845721000	-0.616966000
H	-1.249921000	2.481844000	-1.458750000
C	-1.869041000	0.851275000	-0.107287000
H	-2.736917000	0.437384000	-0.598682000

H	1.336276000	1.597616000	-1.396790000
C	-1.772339000	0.838968000	1.351975000
O	-2.385293000	0.161967000	2.156865000
C	-0.360637000	2.496535000	0.572259000
O	0.394671000	3.446309000	0.579550000
N	-0.784804000	1.792498000	1.700689000
C	-0.725239000	-1.897670000	0.495962000
O	-1.586750000	-2.734412000	0.321595000
O	0.005345000	-1.751011000	1.601200000
C	-0.309972000	-2.664023000	2.681725000
H	-1.354137000	-2.542915000	2.974667000
H	-0.125917000	-3.692666000	2.366340000
H	0.355531000	-2.384319000	3.495549000
C	-0.269501000	1.977113000	3.008945000
C	-1.137452000	2.252404000	4.063393000
C	1.103740000	1.874696000	3.224950000
C	-0.622461000	2.417672000	5.344808000
C	1.609003000	2.056245000	4.507110000
C	0.748336000	2.323727000	5.568900000
H	-2.201318000	2.338522000	3.877463000
H	1.763980000	1.649669000	2.394825000
H	-1.295941000	2.629035000	6.168221000
H	2.677392000	1.979611000	4.677080000
H	1.145597000	2.459109000	6.568926000

Exo-**4b** Etot = -820.647942627948

C	-0.193146000	0.031782000	-2.433421000
C	0.482120000	0.736127000	-1.276847000

C	-0.808263000	-0.726613000	-0.381802000
C	-1.004044000	-0.869773000	-1.882503000
H	-0.098146000	0.315474000	-3.472627000
H	-1.741453000	-1.514779000	-2.338947000
O	0.562271000	-0.306869000	-0.283236000
C	-0.617090000	1.656671000	-0.639268000
H	-1.075623000	2.324048000	-1.368997000
C	-1.547246000	0.619729000	0.008019000
H	-2.587353000	0.622259000	-0.315964000
H	1.450135000	1.206042000	-1.437466000
C	-1.447978000	0.886240000	1.496269000
O	-2.009979000	0.274477000	2.383267000
C	-0.051481000	2.447997000	0.519886000
O	0.745461000	3.361424000	0.457144000
N	-0.577640000	1.942739000	1.699598000
C	-0.179303000	2.410270000	3.025532000
H	0.051873000	3.474126000	2.935537000
H	-1.044826000	2.295710000	3.682268000
C	1.017804000	1.630480000	3.554419000
H	1.878858000	1.746262000	2.891314000
H	0.779880000	0.567083000	3.638916000
H	1.290260000	2.004083000	4.544335000
C	-1.161044000	-1.925534000	0.471516000
O	-2.261286000	-2.424342000	0.332269000
C	-0.125153000	-2.429440000	1.421161000
H	0.241453000	-1.612385000	2.050150000
H	-0.532068000	-3.234925000	2.031245000
H	0.737304000	-2.786558000	0.847097000

TS-*exo*-**4b** Etot = -820.615103432217 i444 cm-1

C	-0.010296000	0.061083000	-2.382345000
C	0.620017000	0.661509000	-1.247244000
C	-0.559509000	-0.976599000	-0.488313000
C	-0.785308000	-0.950143000	-1.891559000
H	0.017527000	0.457181000	-3.386552000
H	-1.506119000	-1.563087000	-2.410552000
O	0.557478000	-0.238959000	-0.206495000
C	-0.892421000	1.797704000	-0.619078000
H	-1.074938000	2.384508000	-1.510018000
C	-1.811793000	0.875311000	-0.075172000
H	-2.720550000	0.521187000	-0.538924000
H	1.493190000	1.302347000	-1.244147000
C	-1.710042000	0.938984000	1.393397000
O	-2.360792000	0.335981000	2.231036000
C	-0.242630000	2.485808000	0.543163000
O	0.546635000	3.412079000	0.517484000
N	-0.701407000	1.864733000	1.694032000
C	-0.229342000	2.189601000	3.034004000
H	-0.197967000	3.280145000	3.119933000
H	-0.981903000	1.814923000	3.731896000
C	1.140006000	1.591540000	3.332680000
H	1.885876000	1.950358000	2.619382000
H	1.109601000	0.500398000	3.286950000
H	1.455597000	1.885157000	4.337057000
C	-1.004657000	-2.002711000	0.475681000
O	-2.004642000	-2.654043000	0.215217000
C	-0.191579000	-2.189968000	1.717431000

H	-0.125893000	-1.250710000	2.273109000
H	-0.641465000	-2.961966000	2.340265000
H	0.832516000	-2.468680000	1.446957000

Exo-**4e** Etot = -973.045271516000

C	-0.222024000	-0.036133000	-2.485382000
C	0.488975000	0.727524000	-1.389851000
C	-0.757604000	-0.696370000	-0.378636000
C	-1.006035000	-0.914060000	-1.862125000
H	-0.165547000	0.196363000	-3.539947000
H	-1.754236000	-1.585243000	-2.259322000
O	0.611350000	-0.263670000	-0.349385000
C	-0.595208000	1.672513000	-0.759154000
H	-1.084171000	2.300161000	-1.504241000
C	-1.493479000	0.665017000	-0.029776000
H	-2.544155000	0.644077000	-0.316521000
H	1.446628000	1.195368000	-1.607850000
C	-1.353658000	1.001128000	1.438926000
O	-1.898952000	0.442664000	2.365710000
C	0.008964000	2.527132000	0.331150000
O	0.794099000	3.437069000	0.189234000
N	-0.463888000	2.068413000	1.564483000
C	-1.073338000	-1.852320000	0.546186000
O	-2.174327000	-2.362313000	0.469415000
C	-0.004291000	-2.299233000	1.487238000
H	0.367505000	-1.449223000	2.067939000
H	-0.383022000	-3.080432000	2.145119000

H	0.847363000	-2.670591000	0.906295000
C	-0.054950000	2.611105000	2.817970000
C	0.590610000	1.790463000	3.737981000
C	-0.304993000	3.950789000	3.098386000
C	0.985111000	2.322233000	4.960526000
C	0.102582000	4.474499000	4.320422000
C	0.744026000	3.662428000	5.251907000
H	0.782693000	0.751719000	3.493498000
H	-0.814834000	4.569903000	2.369114000
H	1.486632000	1.688331000	5.683338000
H	-0.087300000	5.518081000	4.545591000
H	1.056499000	4.074130000	6.205309000

TS-*exo*-**4e** Etot = -973.015181958272 i425 cm-1

C	-0.095330000	0.176077000	-2.424822000
C	0.530824000	0.848367000	-1.325306000
C	-0.410800000	-0.912748000	-0.508034000
C	-0.727595000	-0.910679000	-1.891963000
H	-0.171130000	0.569904000	-3.427415000
H	-1.408316000	-1.598711000	-2.369377000
O	0.624019000	-0.056786000	-0.284293000
C	-0.977727000	1.838971000	-0.598944000
H	-1.220715000	2.468552000	-1.446293000
C	-1.856220000	0.867789000	-0.069440000
H	-2.728990000	0.453886000	-0.551800000
H	1.347153000	1.558621000	-1.380505000
C	-1.753305000	0.876865000	1.388142000

O	-2.370145000	0.222294000	2.208889000
C	-0.322800000	2.504360000	0.577487000
O	0.441962000	3.446274000	0.564881000
N	-0.752534000	1.826202000	1.718815000
C	-0.719234000	-1.934941000	0.510465000
O	-1.641691000	-2.708378000	0.305302000
C	-0.246827000	2.046690000	3.025560000
C	-1.122339000	2.349891000	4.066069000
C	1.125266000	1.953942000	3.253353000
C	-0.615959000	2.552591000	5.345520000
C	1.621793000	2.173165000	4.533000000
C	0.753549000	2.468438000	5.581115000
H	-2.185133000	2.428451000	3.871156000
H	1.791328000	1.706676000	2.434236000
H	-1.295311000	2.785486000	6.158215000
H	2.689216000	2.104006000	4.711921000
H	1.143878000	2.633026000	6.579478000
C	0.137135000	-1.967907000	1.737962000
H	-0.285015000	-2.661343000	2.464020000
H	1.144669000	-2.294885000	1.454570000
H	0.244564000	-0.971998000	2.175648000

8. References

1. Sanchez, A.; Pedroso, E.; Grandas, A. Maleimide-dimethylfuran *exo* adducts: Effective maleimide protection in the synthesis of oligonucleotide conjugates. *Org. Lett.* **2011**, *13*, 4364–4367.
2. Ma, X.-Y.; He, Y.; Lu, T.-T.; Lu, M. Conversion of aldoximes into nitriles catalyzed by simple transition metal salt of the fourth period in acetonitrile. *Tetrahedron* **2013**, *69*, 2560–2564.
3. Grimme, S.; Hansen, A.; Ehlert, S.; Mewes, J.M. r2SCAN-3c: A “Swiss army knife” composite electronic-structure method. *J. Chem. Phys.* **2021**, *154*, 064103.
4. van Wüllen, C. Molecular density functional calculations in the regular relativistic approximation: Method, application to coinage metal diatomics, hydrides, fluorides and chlorides, and comparison with first-order relativistic calculations. *J. Chem. Phys.* **1998**, *109*, 392–399.
5. Weigend, F.; Ahlrichs, R. Balanced basis sets of split valence, triple zeta valence and quadruple zeta valence quality for H to Rn: Design and assessment of accuracy. *Phys. Chem. Chem. Phys.* **2005**, *7*, 3297–3305.
6. Barone, V.; Cossi, M. Quantum calculation of molecular energies and energy gradients in solution by a conductor solvent model. *J. Phys. Chem. A* **1998**, *102*, 1995–2001.
7. Neese, F.; Wennmohs, F.; Becker, U.; Riplinger, C. The ORCA quantum chemistry program package. *J. Chem. Phys.* **2020**, *152*, 224108.
8. Sheldrick, G.M. Crystal structure refinement with SHELXL. *Acta Crystallogr. Sect. C Struct. Chem.* **2015**, *71*, 3–8.
9. Dolomanov, O.V.; Bourhis, L.J.; Gildea, R.J.; Howard, J.A.K.; Puschmann, H. OLEX2: A Complete Structure Solution, Refinement and Analysis Program. *J. Appl. Crystallogr.* **2009**, *42*, 339–341.
10. Sheldrick, G.M. A Short History of *SHELX*. *Acta Crystallogr. Sect. A Found. Crystallogr.* **2008**, *64*, 112–122.
11. Cioc, R.C.; Smak, T.J.; Crockatt, M.; van der Waal, J.C.; Bruijnincx, P.C.A. Furoic acid and derivatives as atypical dienes in Diels-Alder reactions. *Green Chem.* **2021**, *23*, 5503–5510.
